# Precursor reaction kinetics control compositional grading and size of CdSe_1–*x*_S_*x*_ nanocrystal heterostructures†
†Electronic supplementary information (ESI) available. See DOI: 10.1039/c9sc00989b


**DOI:** 10.1039/c9sc00989b

**Published:** 2019-06-05

**Authors:** Leslie S. Hamachi, Haoran Yang, Ilan Jen-La Plante, Natalie Saenz, Kevin Qian, Michael P. Campos, Gregory T. Cleveland, Iva Rreza, Aisha Oza, Willem Walravens, Emory M. Chan, Zeger Hens, Andrew C. Crowther, Jonathan S. Owen

**Affiliations:** a Department of Chemistry , Columbia University , New York , New York 10027 , USA . Email: jso2115@columbia.edu; b The Molecular Foundry , Lawrence Berkeley National Laboratory , Berkeley , CA 94720 , USA; c Department of Chemistry , Barnard College , New York , New York 10027 , USA . Email: acrowthe@barnard.edu; d Physics and Chemistry of Nanostructures Group (PCN) , Ghent University , B-9000 Ghent , Belgium; e Center of Nano and Biophotonics , Ghent University , B-9000 Ghent , Belgium

## Abstract

We report a method to control the composition and microstructure of CdSe_1–*x*_S_*x*_ nanocrystals by the simultaneous injection of sulfide and selenide precursors into a solution of cadmium oleate and oleic acid at 240 °C. Pairs of substituted thio- and selenoureas were selected from a library of compounds with conversion reaction reactivity exponents (*k*_E_) spanning 1.3 × 10^–5^ s^–1^ to 2.0 × 10^–1^ s^–1^. Depending on the relative reactivity (*k*_Se_/*k*_S_), core/shell and alloyed architectures were obtained. Growth of a thick outer CdS shell using a syringe pump method provides gram quantities of brightly photoluminescent quantum dots (PLQY = 67 to 90%) in a single reaction vessel. Kinetics simulations predict that relative precursor reactivity ratios of less than 10 result in alloyed compositions, while larger reactivity differences lead to abrupt interfaces. CdSe_1–*x*_S_*x*_ alloys (*k*_Se_/*k*_S_ = 2.4) display two longitudinal optical phonon modes with composition dependent frequencies characteristic of the alloy microstructure. When one precursor is more reactive than the other, its conversion reactivity and mole fraction control the number of nuclei, the final nanocrystal size at full conversion, and the elemental composition. The utility of controlled reactivity for adjusting alloy microstructure is discussed.

## Abstract 2

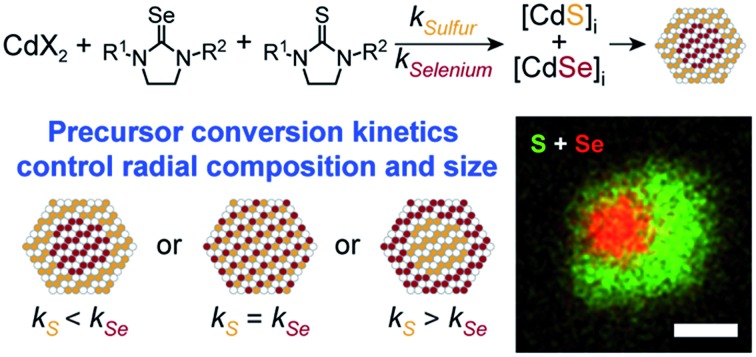
By varying precursor structure, core/shell and alloyed nanocrystal synthesis are performed in a single synthetic step.

## Introduction

Molecular precursors with tailored reactivity can precisely control the rate of solute supply and the extent of nucleation during colloidal crystallizations.^1^–^4^ Recent examples illustrate this principle using libraries of chalcogenone derivatives to synthesize CdS, CdSe, PbS, and PbSe nanocrystals.^5^–^8^ By choosing different substitution patterns, one can control the reactivity, the extent of nucleation, and the size following quantitative precursor conversion.^5^–^7^ Such a method avoids the need to terminate the precursor reaction prematurely, modify the surfactant mixture, or adjust the reaction temperature to prepare a desired size, which can inadvertently change the surface chemistry and composition of the final product.^9^–^15^

**Scheme 1:**
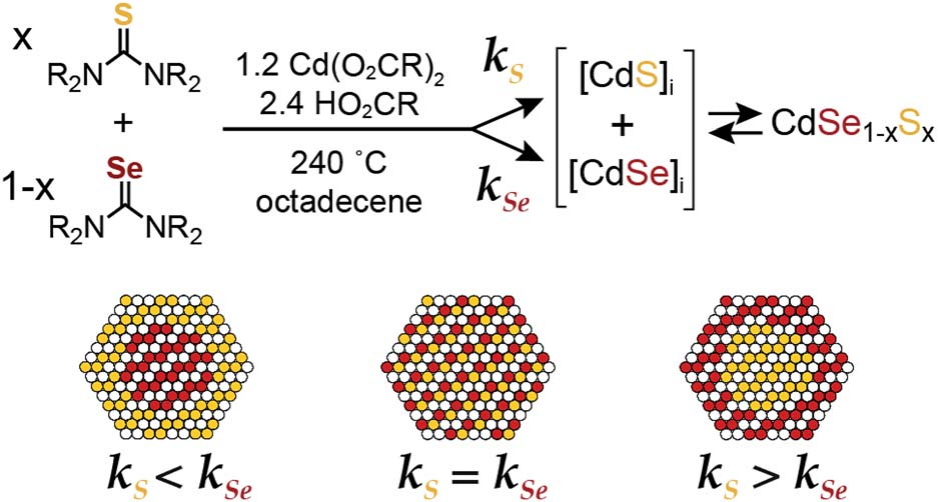


By injecting a mixture of sulfide and selenide precursors with carefully selected conversion reactivity, one can, in principle, adjust the solute evolution during growth and the resultant alloy microstructure (Scheme 1). Such an approach could reduce the number of steps required to prepare CdSe/CdS heterostructures, nanocrystals that are highly desirable luminescent downconverters for solid-state lighting, electronic displays, and biological imaging applications.^16^–^20^ In particular, grading the interface between CdSe and CdS is thought to reduce the rate of Auger recombination,^21^ a multi-exciton recombination process that is sensitive to the microstructure of quantum dots and of great importance to quantum dot lasers and down converting materials for solid state lighting.^22^–^30^ However, recent theoretical and experimental studies on the link between graded alloys and Auger kinetics do not agree on the magnitude of this effect.^21^,^27^,^30^–^32^ Greater control over the compositional grading between interfaces could therefore enable more systematic investigations of multi-exciton photophysics.

CdSe/CdS heterostructures are typically synthesized using layer-by-layer, seeded growth, or one-pot methods where a mixture of sulfur and selenium precursors are coinjected. Layer-by-layer methods provide precise control over composition by slowly injecting precursors *via* syringe pump^22^,^27^,^30^ or *via* successive ion layer adsorption and reaction (SILAR),^26^,^33^ but these methods require extensive reaction times and/or precise half monolayer additions to avoid separate nucleation of the shell material.^22^,^24^,^34^,^35^ Heterostructured and alloyed nanoparticles of CdSe_1–*x*_S_*x*_,^25^,^36^–^41^ CdTe_1–*x*_S_*x*_,^42^ CdTe_1–*x*_Se_*x*_,^43^,^44^ PbSe_1–*x*_S_*x*_, PbTe_1–*x*_S_*x*_, and PbTe_1–*x*_Se_*x*_^45^ are also prepared in a single step by coinjection of phosphine sulfide and selenides,^25^,^36^–^38^,^43^–^45^ bis(trimethylsilyl)chalcogenides,^45^ and elemental chalcogen precursors.^39^–^41^ While it is possible to adjust the luminescence and absorption properties by tuning the ratio of sulfide and selenide precursors, it is unclear how these changes influence the elemental distribution or size of the final product, nor the role of alloy microstructure on photophysical properties.

Finally, the microstructure of heterostructured and alloyed nanoparticles is difficult to probe with a resolution higher than 5 nm, which is on the order of the shell and core thickness of typical colloidal quantum dots. Scanning transmission electron microscopy coupled with energy dispersive spectroscopy (STEM-EDX) or high angle annular dark field (HAADF STEM) imaging are well suited to determining the elemental distribution in large (*d* ≥ 30 nm) particles,^27^,^46^,^47^ but elemental mapping of small particles is much more challenging,^48^ and effects from curvature, anisotropic shell growth, and intermixing are convoluted. Although powder X-ray diffraction is also used to characterize such samples,^49^ Scherrer line broadening and the presence of stacking faults makes it difficult to differentiate between compositions. Raman spectroscopy can probe the composition of CdS_1–*x*_Se_*x*_ alloys, although there are conflicting claims regarding the number of characteristic Raman modes, and it is difficult to distinguish alloy and core/shell quantum dots on the basis of Raman spectroscopy alone.^39^,^50^,^51^ X-ray photoelectron spectroscopy (XPS), photoemission spectroscopy, energy dispersive X-ray spectroscopy (EDX), Rutherford backscattering, and solid-state ^113^Cd and ^77^Se nuclear magnetic resonance (NMR) spectroscopy have all been used to monitor the radial evolution of nanocrystals during growth, but these techniques do not address effects from anisotropy.^29^,^39^,^40^,^51^–^55^ These limitations obscure the study of alloy microstructure and how it is controlled by precursor reactivity.

To gain more precise control over the microstructure and photophysical performance of luminescent CdSe/CdS quantum dots, we synthesized new chalcogenourea precursors, measured their relative reactivities, and explored the one-pot synthesis of alloys and heterostructures. Our results demonstrate how the relative reactivity and the mole fraction of added precursor systematically influence the elemental distribution of CdSe/CdS heterostructures and alloys, as well as their final crystal size.

## Results

**Scheme 2:**
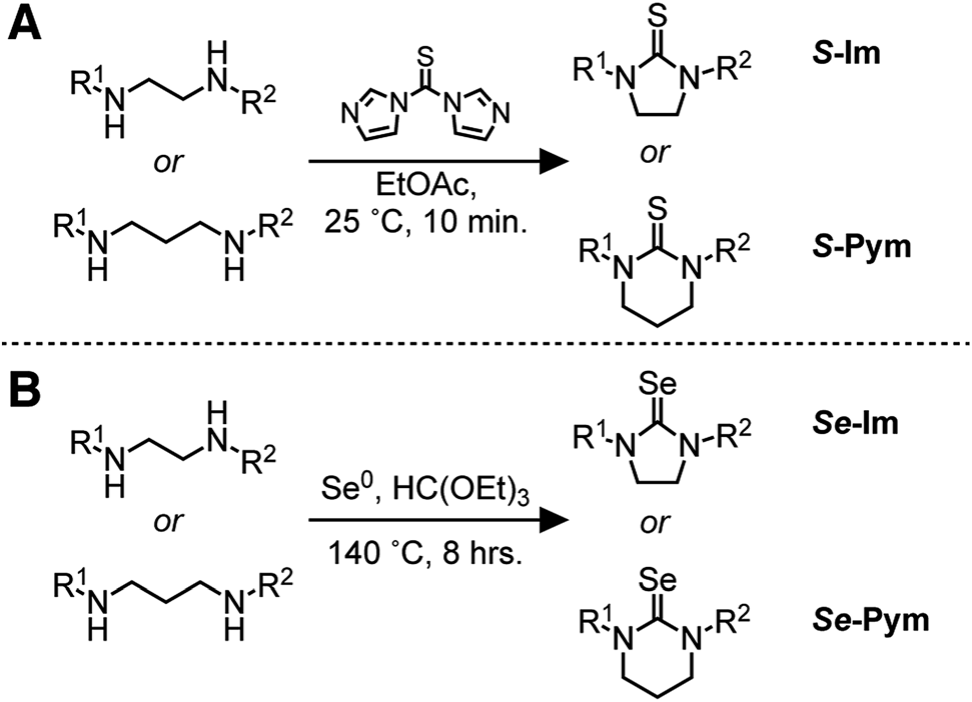


**Fig. 1:**
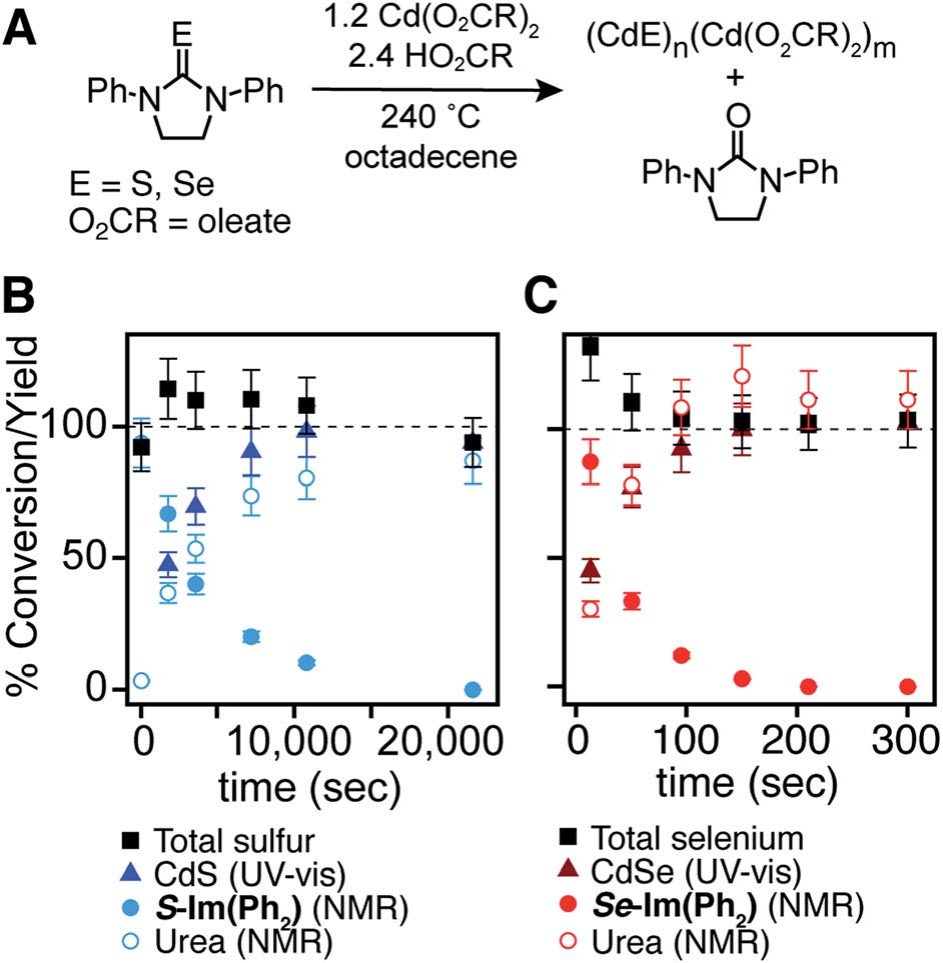
(A) Synthesis of cadmium chalcogenide nanocrystals from (B) **S-Im(Ph_2_)** and (C) **Se-Im(Ph_2_)**. Comparison of mass balance and kinetics as measured by UV-vis absorption and ^1^H NMR spectroscopies. Error bars correspond to 10% error in NMR measurement.

*N*-Monosubstituted and *N*,*N*′-disubstituted imidazolidine selones (***Se*-Im**(R^1^,R^2^) R = H, Me, Et, iPr, *t*-Bu, Ph) and pyrimidine selones (***Se*-Pym**(R^1^,R^2^) R = H, Me, Et, iPr) were used to synthesize CdSe nanocrystals (Scheme 2, Fig. 1 and S1†). ***Se*-Im** and ***Se*-Pym** can be prepared in a single synthetic step by refluxing diamines with triethyl orthoformate and elemental selenium.^56^ With the exception of *N*,*N*′-di-*tert*-butyl derivatives and ***Se*-Pym**(R^1^,R^2^) compounds, > 50% yields of analytically pure material can be isolated following recrystallization from ethyl acetate or acetonitrile.

The corresponding thiones (***S*-Im** and ***S*-Pym**) are synthesized from the *N*,*N*′-substituted diamines and carbon disulfide, thiophosgene,^57^–^59^ or thiocarbonyldiimidazole.^59^,^60^ To avoid toxicity concerns, all thiones in this study were prepared from thiocarbonyldiimidazole. ^1^H NMR studies show that the second C–N bond forming step is inhibited by *tert*-butyl substituents, which can lead to oligomerization rather than cyclization. Similarly, syntheses of ***S*-Pym** from *N*,*N*′-disubstituted-1,3-diaminopropanes provided lower yields and must be conducted under dilute conditions to prevent oligomerization.^58^

To improve the reproducibility and convenience of the nanocrystal synthesis, 50 gram quantities of cadmium oleate were isolated from cadmium trifluoroacetate, triethylamine, and oleic acid in acetonitrile and stored in a nitrogen filled glove box prior to use. In this way, water and/or acetic acid generated during the synthesis of cadmium carboxylates from cadmium oxide or cadmium acetate can be avoided.^61^ Tetraglyme solutions of chalcogenoureas were injected into a solution of oleic acid and cadmium oleate at 240 °C, producing CdS and CdSe nanocrystals over the course of minutes to hours depending on the precursor structure (Fig. 1).

Reaction kinetics are monitored by diluting aliquots to a known concentration before analysis with UV-visible absorption and NMR spectroscopy. The disappearance of the precursors and the formation of the corresponding urea and carboxylic anhydride coproducts^4^–^7^ match the yield of CdSe^62^ and CdS^7^ measured with absorption spectroscopy (Fig. 1). This observation allows the precursor conversion kinetics to be estimated by monitoring nanoparticle formation using UV-vis spectroscopy. A similar strategy has been used in our previous studies of CdS,^7^ CdSe,^4^ PbS,^5^ and PbSe.^6^

A single exponential fit of the conversion and yield are used to extract a reactivity exponent (*k*_E_) for each precursor (s^–1^) (Table 1). These are distinct from a first order conversion reaction rate constant because the kinetics experiments are not run under pseudo first order conditions and appear to follow higher order kinetics. Regardless, the exponents provide a way to conveniently order the relative conversion reactivity over several orders of magnitude under a standard set of conditions. Five orders of magnitude in reactivity are apparent, with many pairs of sulfur and selenium precursors within an order of magnitude of each other. A few of the selenourea precursors produce a partial yield of CdSe, and an initial rate was used to estimate the relative reactivity (see ESI†). These include ***Se*-Im(H,Ph)**, ***Se*-Im(H,Et)**, ***Se*-Im(H,iPr)**, ***Se*-Im(Me_2_)**, ***Se*-Im(*t*-Bu_2_)**, ***Se*-Pym(H,Me)**, ***Se*-Pym(Me_2_)**, and ***Se*-Pym(Et_2_)**, which typically produce between 50–75% yields. An explanation for the low yield was not pursued, and with the exception of ***Se*-Im(*t*-Bu_2_)** these selenoureas are not used further below.

**Table 1:** Chalcogenourea precursors and their reactivity

Compound	E	R_1_	R_2_	*k*_E_ ^*a*^ (s^–1^)	Initial rate^*b*^ (mM s^–1^)	% Yield^*a*^ [CdE]
***S*-Im(H,Ph)**	S	H	Ph	2.5 × 10^–3^	2.1 × 10^–2^	93
***S*-Im(H,Me)**	S	H	Me	1.2 × 10^–3^	1.3 × 10^–2^	100
***S*-Im(H,Et)**	S	H	Et	6.6 × 10^–4^	5.6 × 10^–3^	100
***S*-Im(Ph_2_)**	S	Ph	Ph	2.2 × 10^–4^	3.7 × 10^–3^	98
***S*-Pym(H,Me)**	S	H	Me	1.6 × 10^–4^	2.1 × 10^–3^	100
***S*-Im(H,iPr)**	S	H	iPr	1.3 × 10^–4^	2.0 × 10^–3^	100
***S*-Im(Me_2_)**	S	Me	Me	6.5 × 10^–5^	3.9 × 10^–4^	100
***S*-Pym(Me_2_)**	S	Me	Me	5.5 × 10^–5^	9.9 × 10^–4^	100
***S*-Im(Et_2_)**	S	Et	Et	2.2 × 10^–5^	2.9 × 10^–4^	100
***S*-Pym(Et_2_)**	S	Et	Et	1.8 × 10^–5^	3.4 × 10^–4^	100
***S*-Pym(H,iPr)**	S	H	iPr	1.6 × 10^–5^	6.0 × 10^–4^	96
***S*-Im(iPr_2_)**	S	iPr	iPr	1.5 × 10^–5^	6.6 × 10^–4^	100
***S*-Pym(iPr_2_)**	S	iPr	iPr	1.3 × 10^–5^	1.5 × 10^–4^	100
***Se*-Im(H,Ph)**	Se	H	Ph	2.0 × 10^–1^	3.7 × 10^–1^	44
***Se*-Im(H,Et)**	Se	H	Et	6.3 × 10^–2^	3.5 × 10^–1^	79
***Se*-Im(H,iPr)**	Se	H	iPr	4.0 × 10^–2^	1.8 × 10^–1^	64
***Se*-Im(Ph_2_)**	Se	Ph	Ph	2.0 × 10^–2^	2.9 × 10^–1^	98
***Se*-Pym(H,Me)**	Se	H	Me	1.5 × 10^–2^	8.0 × 10^–2^	75
***Se*-Im(*t*-Bu_2_)**	Se	*t*-Bu	*t*-Bu	7.4 × 10^–3^	4.1 × 10^–2^	53
***Se*-Im(Me_2_)**	Se	Me	Me	3.3 × 10^–3^	2.6 × 10^–2^	82
***Se*-Pym(Me_2_)**	Se	Me	Me	2.2 × 10^–3^	3.3 × 10^–2^	81
***Se*-Im(Et_2_)**	Se	Et	Et	1.3 × 10^–3^	1.1 × 10^–2^	77
***Se*-Pym(Et_2_)**	Se	Et	Et	1.0 × 10^–3^	1.1 × 10^–2^	70
***Se*-Pym(iPr_2_)**	Se	iPr	iPr	9.5 × 10^–4^	1.2 × 10^–2^	98
***Se*-Im(iPr_2_)**	Se	iPr	iPr	2.1 × 10^–4^	4.0 × 10^–3^	98

^*a*^Kinetics measured by UV-Vis absorption under standard reaction conditions: chalcogenourea (10 mM), cadmium oleate (12 mM), oleic acid (24 mM) at 240 °C. Uncertainty (25%) in the rate constant is estimated from the uncertainty in the extinction coefficient (10%) described in the ESI.

^*b*^Initial rate estimated from the slope of the first two UV-vis absorption aliquots (see ESI for examples).

Several reactivity trends emerge from the range of *k*_E_. Cyclic precursors are substantially less reactive than acyclic structures with the same substituents. For example, ***S*-Im(Me_2_)** is less reactive than tetramethylthiourea by an order of magnitude. Sterically encumbered precursors are also less reactive; replacing methyl with isopropyl causes an order of magnitude reduction in reactivity. An exception is ***Se*-Im(*t*-Bu_2_)**, which is anomalously reactive and likely follows a different conversion mechanism. In many cases, *N*-alkyl-substituted precursors are less reactive than *N*-aryl-substituted precursors. Pyrimidine chalcogenones are typically less reactive than the imidazolidine chalcogenones, perhaps because their substituents are more directed toward the chalcogen atom and have a greater steric influence. All of these substituent effects can be explained by a mechanism involving Lewis acidic activation of the chalcogenourea prior to cleavage of the C=E bond, a mechanism analogous to the one proposed for phosphine chalcogenides and thiocarbonates.^7^,^63^

If the C=E bond cleavage reaction is preceded by a fast preequilibrium Lewis acid activation, one precursor could, in principle, reduce the available cadmium and the conversion kinetics of the other. In one example studied here the conversion kinetics of a mixed precursor synthesis was monitored using ^1^H NMR spectroscopy and compared to the kinetics of the single component reactions (see ESI†). In that case, the conversion reactivity of the chalcogenoureas appears orthogonal, *i.e.* it is not altered by the presence of a second precursor. A complete analysis of these effects requires a detailed study beyond the scope of this report, and the role of the conversion reaction mechanism or the orthogonality of the precursor reactions is not considered further below.

**Fig. 2:**
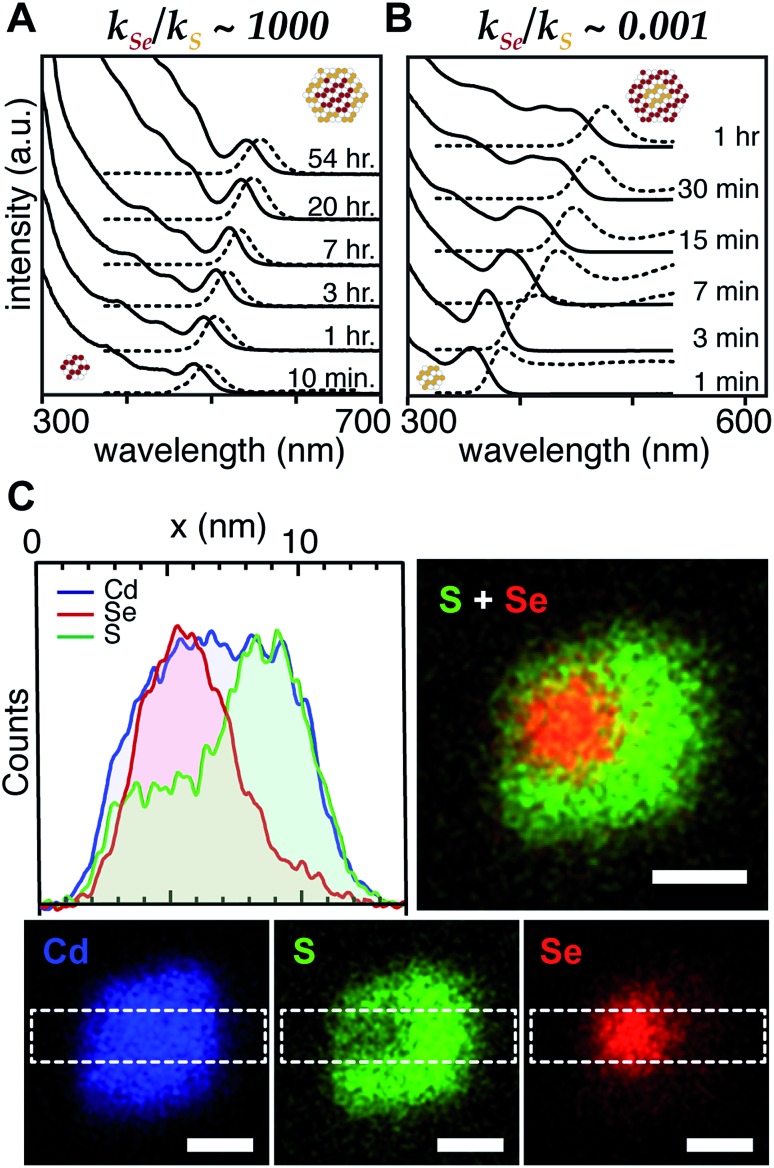
Example UV-vis and photoluminescence spectra of (A) CdSe/CdS core/shell and (B) CdS/CdSe core/shell nanocrystals synthesized from (A) *N*-butyl-*N*′-pyrrolidine selenourea and ***S*-Im(Me_2_)** and (B) *N*-dodecyl-*N*′-hexylthiourea and ***Se*-Im(Et_2_)**. (C) STEM EDX elemental maps of nanocrystals synthesized from ***Se*-Im(*t*-Bu_2_)** and ***S*-Im(Me_2_)** (blue = cadmium, red = selenium, green = sulfur.) STEM EDX line scan collected in area denoted by dashed white box. Scale bars are 4 nm in length.

With a range of precursor reactivity in hand, we explored the synthesis of CdSe_1–*x*_S_*x*_ nanocrystals using a mixture of two precursors with appropriately matched reactivity. We sought to evaluate whether the relative reactivity could be used to dictate the microstructure according to Scheme 1. When a fast selenium precursor and a much slower sulfur precursor (*N*-butyl-*N*′-pyrrolidineselenourea and ***S*-Im(Me_2_)**, *k*_Se_/*k*_S_ ∼ 1000) are coinjected, a CdSe nanocrystal nucleates and grows to 2.2 nm, as can be observed in the UV-vis absorption spectra of timed aliquots. After growth of the CdSe core, CdS deposition increases the absorbance at high energy (<512 nm) and red shifts the absorption onset (Fig. 2A). At the same time, the photoluminescence quantum yield (PLQY) increases, signaling that CdS is depositing on the CdSe core. No evidence for the nucleation of pure phase CdS was obtained under these conditions.

The inverse architecture is obtained when a reactive sulfur precursor is paired with a slower selenium precursor (*N*-dodecyl-*N*′-hexylthiourea and ***Se*-Im(Et_2_)**, *k*_Se_/*k*_S_ ∼ 0.001). CdS rapidly nucleates upon which CdSe deposits over an hour, shifting the absorption edge into the visible. The expected CdS/CdSe core/shell heterostructure is supported by the evolution of the spectrum during growth (Fig. 2B), and a long photoluminescence decay time and low PLQY (ESI†) that is consistent with a quasi-Type II band structure. Unfortunately, in these examples, the nanocrystals were too small to characterize using STEM EDX.

**Scheme 3:**
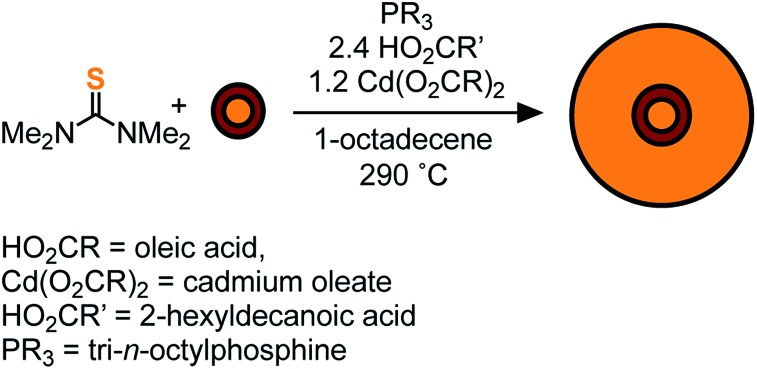


In order to synthesize larger CdSe/CdS nanocrystals suitable for elemental mapping, ***Se*-Im(*t*-Bu_2_)** and ***S*-Im(Me_2_)** (*k*_Se_/*k*_S_ ∼ 100) are co-injected (see ESI†). The spectral evolution of aliquots, and the photoluminescence quantum yield (60%) and photoluminescence lifetime (23 ns) are characteristic of a CdSe/CdS core/shell architecture. Scanning transmission electron microscopy and energy dispersive X-ray spectroscopy (STEM EDX) support the expected CdSe/CdS microstructure although the CdS shell and the CdSe core regions are anisotropically distributed (Fig. 2C). Despite the anisotropy of the shell growth, the observed microstructure supports the claim that the more reactive precursor forms the nanocrystal core.

**Fig. 3:**
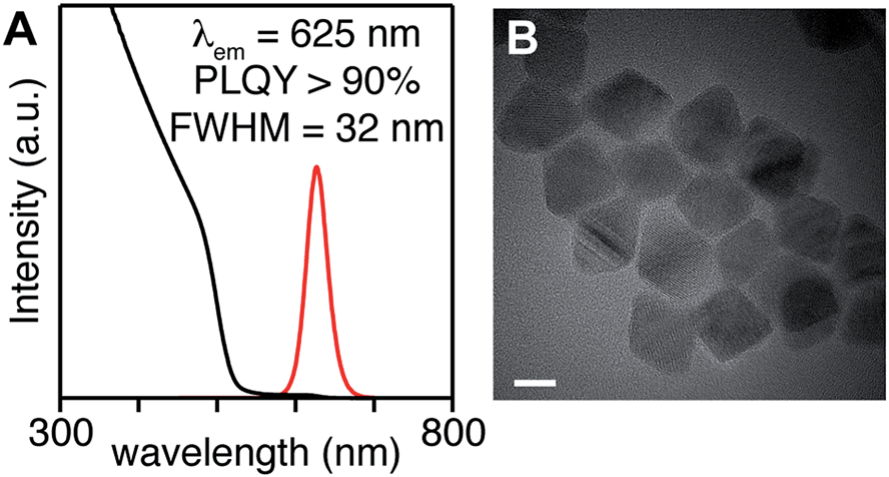
(A) UV-vis and photoluminescence spectra, (B) TEM of CdS/CdSe/CdS spherical quantum wells whose synthesis is described in the ESI.† Scale bar is 10 nm in length.

To access heterostructures desirable for photoluminescence applications, a method was developed to shell the CdSe_1–*x*_S_*x*_ nanocrystals described above with CdS. The low reactivity of tetraalkylthioureas toward cadmium oleate at room temperature allows their mixture to be slowly added to a solution of cores using a syringe pump. Dropwise addition at 240 °C produces nanocrystals up to 6 nm in diameter. Attempts to grow the nanocrystals beyond this diameter caused precipitation during growth. However, upon adding tri-*n*-octylphosphine and 2-hexyldecanoic acid to the shelling solution and increasing the temperature to 290 °C, even thicker shells could be grown without precipitation of the nanocrystals. Using this method, CdS/CdSe/CdS spherical quantum wells^24^ with PLQY up to 90% could be grown in a single pot (Scheme 1) (Fig. 3). Photoluminescence decay times (30–90 ns) increase as the CdS shell thickens. Much like the CdSe/CdS core/shell structures above, the final shape appears to be bipyramidal with the selenium off-center.^64^–^66^ The final nanocrystals are primarily composed of CdS in the outer shell, which combined with the ease of scaling the syringe pump addition, greatly facilitates the preparation of multi-gram quantities of brightly luminescent materials.

**Fig. 4:**
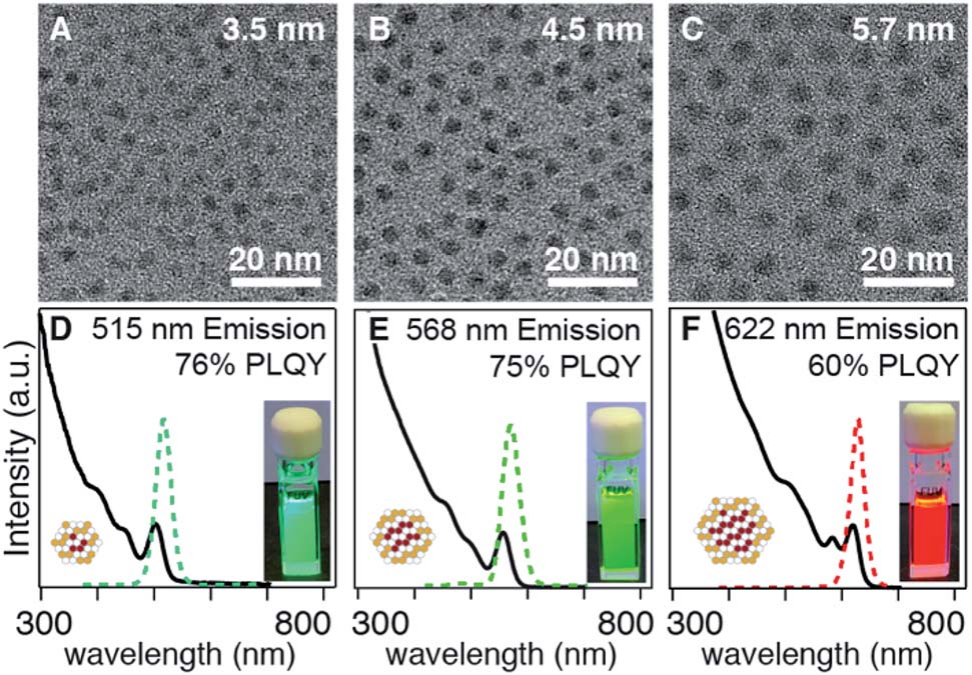
Size and photoluminescence wavelength tunability of CdSe/CdS core/shell nanocrystals made using *N*-butyl-*N*′-pyrrolidine selenourea (A, D), ***Se*-Im(Ph_2_)** (B, E), and ***Se*-Im(*t*-Bu_2_)** (C, F) paired with a slower sulfur precursor ***S*-Im(Me_2_)**. Note that the yield of ***Se*-Im(*t*-Bu_2_)** reaches 50%, an effect that decreases the Se : S ratio of the final structure, and will increase the thickness of the CdS shell. These two effects have opposite effects on the band gap. Note that the reaction with N-butyl-N′-pyrrolidine is done at a lower concentration to avoid mixing limited kinetics.

In the examples above, the flux of solute during nucleation is dominated by the more reactive precursor. Provided the precursors are orthogonal to the crystallization, the solute supplied by the more reactive precursor will dictate the number of nuclei and the final size. For example, when several different selenium precursors are used together with a less reactive thiourea coreactant (*k*_E_ = 10^–1^ to 10^–3^ s^–1^: *N*-butyl-*N*′-pyrrolidine selenourea, ***Se*-Im(Ph_2_)**, or ***Se*-Im(*t*-Bu_2_)**; *vs. k*_E_ = 10^–4^ to 10^–5^ s^–1^: tetramethylthiourea or ***S*-Im(Me_2_)**) nanocrystals with a range of emission wavelengths are obtained (*λ*_max_ = 515–622 nm) including blue-green emitters that are otherwise challenging to synthesize (Fig. 4). TEM analysis shows that the nanocrystal size (*d* = 3.5–5.7 nm) tracks inversely with the reactivity of the selenium precursor. Given that the mole fraction of the precursors is fixed across these experiments, (33 : 66), we conclude that the shift in wavelength is a consequence of the change in size. The difference in final volume corresponds to a ∼4-fold change in the number of nuclei, behavior that is consistent with the extent of nucleation of pure phase CdSe cores discussed below (see below).

**Fig. 5:**
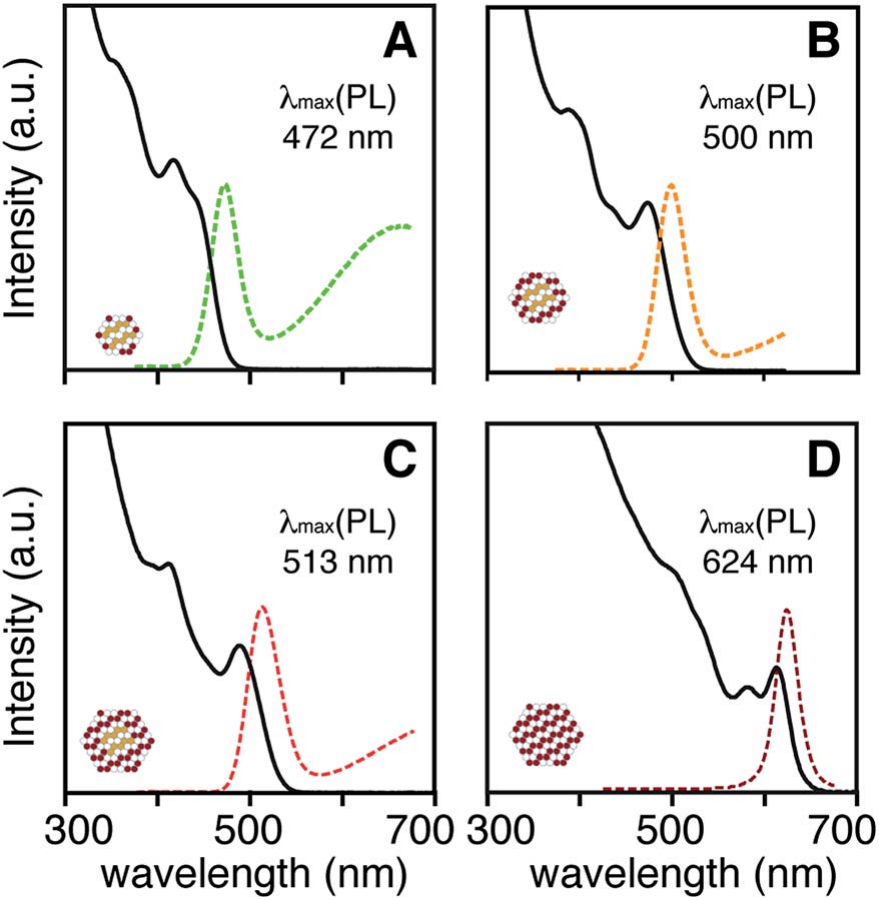
Absorption (solid line) and photoluminescence (dashed line) of CdS/CdSe core/shell nanocrystals synthesized by adjusting ratio of *N*-hexyl-*N*′-dodecyl thiourea and ***Se*-Im(Et_2_)** (A) S : Se = 0.5 : 0.5, (B) S : Se = 0.25 : 0.75, (C) S : Se = 0.2 : 0.8, (D) S : Se = 0.0 : 1.0. The broad fluorescence feature shown in A–C arises from trap emission.

Adjusting the ratio of sulfur and selenium precursors to tune the composition and emission wavelength can also influence the rate of solute generation and the extent of nucleation. For example, the emission wavelength of CdS/CdSe core/shell nanocrystals could be tuned from *λ*_max_ = 472 to 513 nm by reducing the amount of *N*-hexyl-*N*′-dodecylthiourea co-injected with ***Se*-Im(Et_2_)** (*k*_Se_/*k*_S_ ∼ 0.001) from 0.5–0.2 equivalents (Fig. 5). Consistent with the compositional variation, the luminescence color red-shifts with the increased mole fraction of selenium. However, the shift can also be affected by the final size, which is unfortunately too small to accurately measure in the examples shown in Fig. 5.

To understand how adjusting the amount of the more reactive precursor dictates the extent of nucleation and the final size, pure phase CdS and CdSe nanocrystals were prepared by adjusting the amount of added chalcogenourea (0.1–1 equiv.: *N*-hexyl-*N*′-dodecylthiourea, *N*-hexyl-*N*′,*N*′-dibutylthiourea) or ***Se*-Im(Ph_2_)** under otherwise identical conditions (see ESI†). As the amount of injected chalcogenourea increases, the number of nuclei increases linearly, a change that will increase the number of core nanocrystals on which to grow shell material. Interestingly, nucleation is not observed below a threshold concentration of injected precursor (thiourea ∼1 mM, selenourea ∼0.1 mM), which may reflect a solubility limit of the monomers that is greater for CdS than CdSe. In the synthesis of CdS/CdSe core/shell nanocrystals shown in Fig. 5, increasing the amount of *N*-hexyl-*N*′-dodecylthiourea should therefore reduce the final size and enhance the blue shift for quantum confinement reasons.

**Fig. 6:**
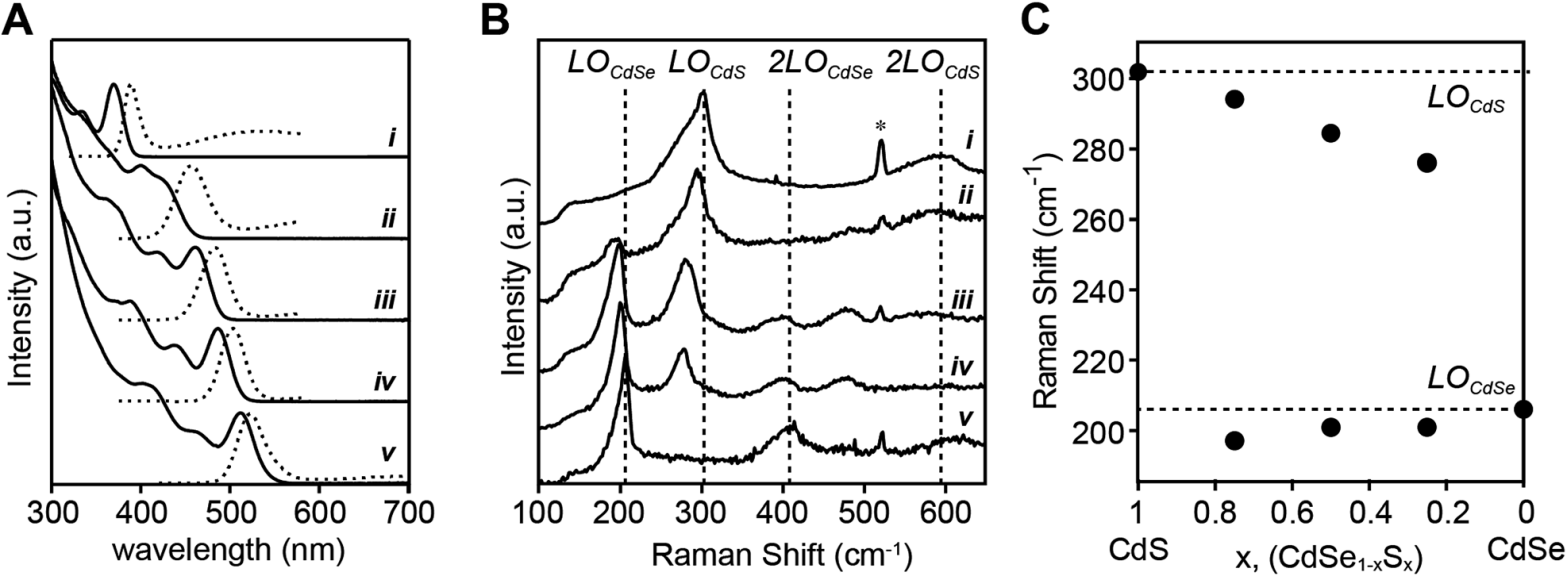
(A) UV-vis and photoluminescence spectra of CdS (i), CdSe_0.25_S_0.75_ (ii), CdSe_0.5_S_0.5_ (iii), CdSe_0.75_S_0.25_ (iv), and CdSe (v) nanocrystals. (B) 77 K Raman spectra (*λ*_excite_ = 406 nm) of same alloys showing 2-mode behavior. (C) Peak frequencies of Raman modes *vs.* the mole fraction of sulfur.

Alloys were prepared using a pair of precursors whose conversion reactivity is closely matched (*N*-methyl-*N*,*N*′-diphenyl thiourea and ***Se*-Im(Ph_2_)**, *k*_Se_/*k*_S_ ∼ 2.4) (Fig. 6A). With nearly matched reactivity, both the sulfide and selenide solute are produced at rates that reflect the starting ratio of precursors throughout the reaction and can allow substantial intermixing of the phases. The emission wavelengths of the alloys span the blue/green region of the spectrum with the endpoints defined by pure phase CdS (*d* = 2.1 nm) and CdSe (*d* = 3.0 nm) materials prepared from the constituent precursors. Although the final size of the alloys is too small to be measured accurately with TEM, we were able to determine the size using X-ray pair distribution function analysis (these methods will be reported elsewhere). The sizes and lattice constants of these materials smoothly span the range of sizes produced in single component reactions, and follow Vegard's law (*e.g.* CdS: *d* = 2.1 nm; *a* = 5.81 Å, and CdSe: *d* = 3.0 nm; *a* = 6.09 Å). These data are consistent with the formation of an alloyed composition, and indicate that the extent of nucleation from the mixed solute is similar to behavior of a pure phase synthesis. We conclude that the extent of nucleation is similar to the pure phase and core–shell nanocrystals and is controlled by the rate of solute generation during nucleation.

The closely matched precursor reactivity intermixes the CdS and CdSe as can be seen in Raman spectra acquired at 77 K (Fig. 6B). Two vibrational bands at ∼200 cm^–1^ and ∼300 cm^–1^ are assigned to CdSe and CdS phonon modes, respectively. Each band is fit to two peaks, a more intense longitudinal optical (LO) phonon with a lower energy surface optical (SO) phonon shoulder (see ESI† for more detail on fitting methodology and peak assignments). The LO peaks red-shift from the frequency of pure CdSe or CdS as its mole fraction is reduced, consistent with the literature on bulk^67^ and nanoscale^51^ CdSe_1–*x*_S_*x*_ alloys. The presence of two Raman LO peaks is characteristic of a CdSe_1–*x*_S_*x*_ alloy because the mass of sulfur is less than the reduced mass of CdSe (*m*_S_ < *μ*_CdSe_) which meets a criterion for two-mode behavior.^67^ In contrast, recent studies report single mode Raman spectra of alloyed CdSe_1–*x*_S_*x*_ nanocrystals at a Se : S ratio near 50 : 50.^39^,^50^ We, and others,^51^ have been unable to reproduce this result, making it unclear whether the observed single mode behavior is anomalous or an aspect of a different microstructure (see ESI†). We conclude that the frequency dependence of the Raman bands on the mole fraction of sulfur and selenium is consistent with an alloyed material.

## Discussion

### Compositional grading

**Fig. 7:**
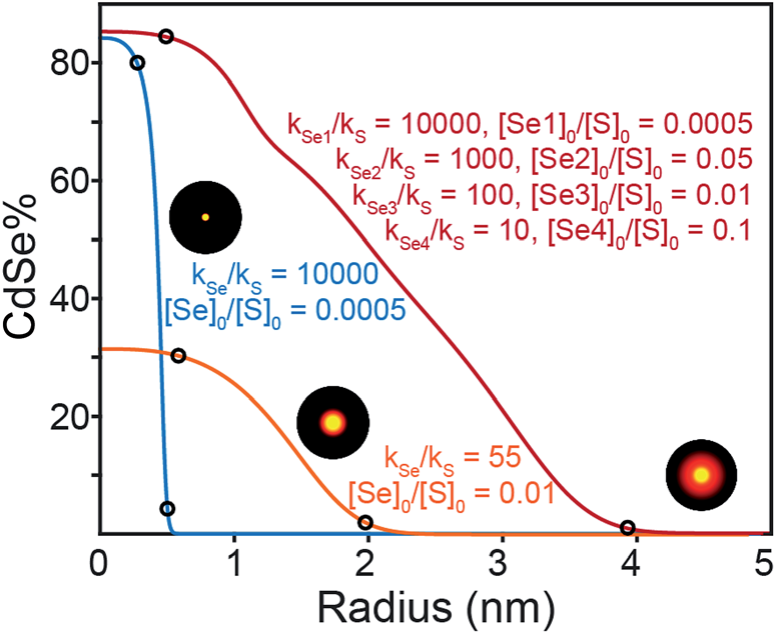
Simulated radial elemental distributions from the first order rate constants shown. [Se]_0_ and [S]_0_ denote the initial concentrations of the Se and S precursors, respectively. [Se1], [Se2], [Se3], and [Se4] indicate Se precursors with different reactivities. The final nanocrystal size is chosen to be 5 nm in all cases.

To understand the influence of the precursor reactivity on the final composition, the elemental distribution was simulated using a rate equation model based on several simplifying assumptions: (1) the rate at which sulfide or selenide deposit on the nanocrystal surface is equal to the instantaneous solute generation rate (M s^–1^), (2) solute generation exhibits first order kinetics in the chalcogen precursor, *i.e.* the rate of solute generation is the product of the chalcogenourea concentration and the reactivity exponent (*k*_E_[E]), (3) the nanocrystals are spherical and grow isotropically without preference for one chalcogenide or the other. Many complicating scenarios could lead to deviations from this simplified behavior including: a surface reaction limited growth mechanism that selectively favors sulfide or selenide deposition, or causes anisotropic growth, or the finite size of the nucleus, which could skew the composition of the core. Regardless, using these inputs, we simulated the radial profile of nanocrystals produced from a wide range of Se : S precursor ratios and relative conversion reactivity (Fig. 7).

A number of useful observations can be made from these simulations. (1) Solute supply kinetics must be within an order of magnitude to substantially alloy the two components, while precursors with >10× difference in reactivity provide abrupt (>90% pure) interfaces. Thus to obtain a homogeneously distributed alloy by injecting a pair of sulfide and selenide partners requires precisely matched reactivity. (2) Heterostructures with thick outer shells require a large amount of shell precursor. The large amount of shell precursor increases the rate at which the shell material is generated during nucleation. This can cause alloying of the core and shell unless the two precursors have very different reactivity. For example, a heterostructure with a 4 nm CdSe core and a 3 nm thick CdS shell is ∼90% CdS by volume and requires ∼10× the amount of sulfide precursor. To prepare such a heterostructure with core and shell regions that are >90% phase pure, the reactivity of the precursor that nucleates the core must be at least 100× greater than the shell precursor. A further complication is the threshold amount of precursor required to cause nucleation, which in the case of CdSe (∼0.2 mM) is much smaller than CdS (∼0.9 mM). The difference suggests that pure phase CdSe nuclei may be more likely to form from a mixed solute composition. More detailed work is required to understand the influence of solute that is below the nucleation threshold. These factors limit the range of compositions that can be tuned from a single injection. Thus, a single injection synthesis of the kind presented above requires precise control over both the reactivity and the mole fraction of the precursors, especially if the size of the final nanocrystal is to be controlled.

Synthesizing higher fidelity structures (*e.g.* with phase pure cores, a thick graded interface, and a phase pure shell) could, in principle, be achieved with precursors that have “laddered” reactivity (Fig. 7, red trace). However, as the nanocrystals grow to large sizes and the outer layers make up a greater fraction of the whole, the range of reactivity that is required to achieve precisely composed and distinct regions leads to impractically long reaction times. We estimate that several days of reaction time are required to produce pure phase core/graded interface/pure phase shell structures with radii >10 nm from a single injection of precursors (see ESI†). Although a variable temperature profile can further optimize the timing, limitations on the range of useful concentration, reaction volume, time, temperature, and reagent stability will also limit the utility of more complex precursor mixtures.

### Size control

Adjusting the reactivity and the mole fraction of each precursor also influences the extent of nucleation and the final nanocrystal size. This is clearly observed in Fig. 4 and 5 above, where increasing the reactivity or the mole fraction of the precursor that causes nucleation reduces the final size and blueshifts the emission wavelength. Similar behavior is observed when synthesizing alloys using sulfide and selenide precursors with matched conversion reactivities. The extent of nucleation is similar to a linear combination of the number of pure phase nanocrystals, weighted by the mole fraction of each precursor. The result is surprising given that the nucleation barrier can be expected to depend on the composition of the nucleus. While the influence of the solute composition on nucleation is deserving of additional study, it is clear that the precursor reactivity remains a powerful tool to tailor the size of heterostructures and alloys.

**Fig. 8:**
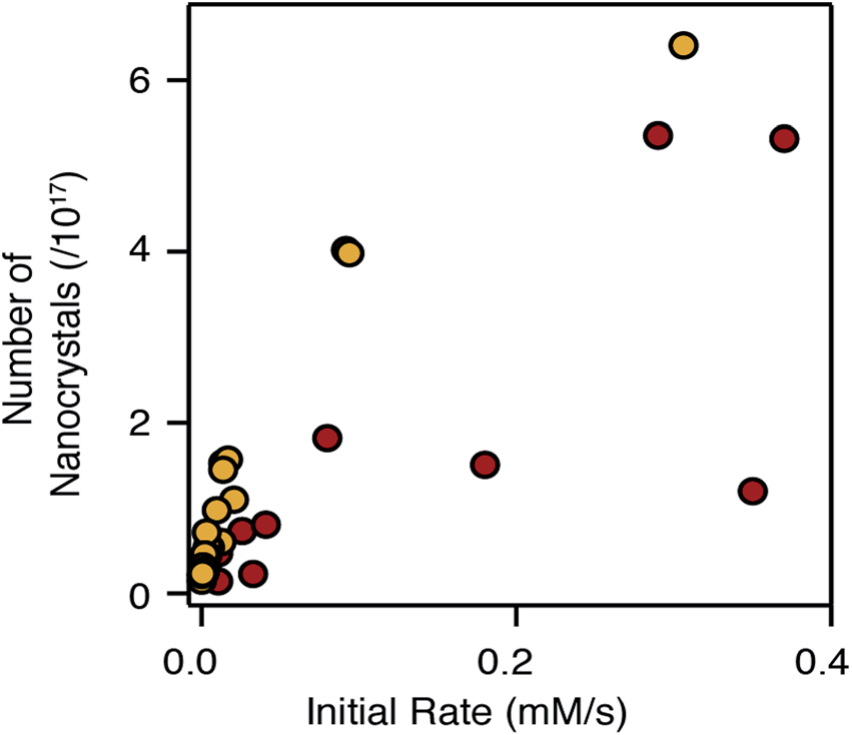
Number of nanocrystals *vs.* initial rate of reaction; CdS (yellow) and CdSe (red).

The number of pure phase CdSe and CdS nuclei produced from the chalcogenoureas is plotted *versus* the initial precursor reaction rate in Fig. 8. Faster conversion reactivity nucleates greater numbers of nanocrystals as has been described earlier.^2^,^4^–^7^,^68^ Interestingly, the nucleation of CdS and CdSe exhibit different dependences on the conversion reactivity, that may arise from differences in the growth kinetics and/or nucleus size.^1^ Compared to CdSe, greater numbers of CdS nanocrystals nucleate at a given solute supply rate, and in several cases, nucleation of CdSe nanocrystals does not follow a simple trend.

Changing the S : Se ratio, as is often used to change the composition of nanocrystals in an alloy synthesis,^39^,^43^,^52^ can have a complex influence over the emission wavelength of the final nanocrystal. While increasing the S : Se ratio generally blue shifts the optical spectrum, the change will also adjust the number of particles nucleated, particularly if the precursors have very disparate reactivity. Whether the number of nanocrystals increases or decreases depends on which precursor is more reactive. When *k*_S_ ≪ *k*_Se_, increasing the S : Se reduces the amount of selenium precursor that causes nucleation, increases the nanocrystal size and reduces the confinement; these effects counteract the blue shift. On the other hand, when *k*_S_ ≫ *k*_Se_, an increase in the S : Se ratio decreases the size and further blue shifts the emission; a cooperative effect on the emission wavelength. Moreover, Fig. 8 suggests these effects will be greater when nucleating CdS, which is more sensitive to the conversion reactivity.

The appearance of multiple nanocrystal populations occurred in a significant fraction of reactions studied over the course of an extensive synthetic investigation (see ESI†). Heterogeneity during the nucleation of pure phase CdSe and the homogeneous nucleation of shell material, especially during the growth of larger heterostructures, were both observed. In several cases, the homogeneity could be improved by adding oleic acid or adjusting the total reaction concentration, while in others these changes were not effective. Although the origins of this behavior are unclear, the incomplete yield produced by several of the selenoureas, the bimodal distributions observed during the synthesis of pure phase CdSe, and the variations in the number of CdSe nanocrystals across the range of reactivity in Fig. 8, suggest that the reactivity of the selenourea precursors may not be orthogonal to the crystallization in all cases.

On the other hand, reactions that first nucleate CdS reliably produce single populations of emitters with a narrow FWHM across a range of thiourea and oleic acid concentrations (0.1–1 equiv. of thiourea and 0–10 equiv. of oleic acid).^7^ Thus, CdS/CdSe/CdS spherical quantum well microstructures were more reliably prepared with narrow photoluminescence than the more traditional CdSe/CdS architecture. Although the origins of the multiple populations proved too complex to rationalize in all cases, the results described herein are reproducible and appear to be a meaningful consequence of the growth mechanism rather than a signature of irreproducible reactivity. Moreover, we were able to optimize one pot syntheses of several desirable architectures that are described in the ESI,† including narrow-band cyan emitters that are challenging to produce by conventional methods and thick shell spherical quantum wells with PLQY near 90%.^25^,^39^,^52^

## Conclusion

We report the synthesis of new cyclic thione and selone precursors whose reactivity with cadmium oleate at 240 °C can be used to control the composition of CdSe_1–*x*_S_*x*_ heterostructures and alloys. These precursors are the least reactive among a library of chalcogenoureas that can be used to synthesize metal chalcogenide nanocrystals, extending the range of reactivity by several orders of magnitude. By coinjecting a pair of thione and selone precursors, the extent of nucleation and the intermixing of selenide and sulfide regions can be controlled to form heterostructures or alloys. The number of nanocrystals that nucleate depends on the relative reactivity and the molar ratio of the co-injected precursors, in a manner that enables precise control of the final architecture. The dependence of the size and composition on the mole ratio and reactivity ratio underscores the importance of precursor design and controlled and reproducible reactivity. Advances along these lines can lead to economical methods to manufacture complex heterostructures in fewer synthetic steps.

## Experimental

### General considerations

All manipulations were performed in air unless otherwise indicated. Toluene (99.5%), methyl acetate (99%), ethyl acetate (≥99.8%), benzene (99.8%), hexanes (98.5%), methanol (99.8%), ethanol (≥99.8%), dichloromethane (≥99.5%), chloroform (≥99.8%), acetone (≥99.8%), acetonitrile (99.5%), cadmium nitrate tetrahydrate (98%), sodium hydroxide (≥98%), sodium bicarbonate (≥99.7%), hydrochloric acid (37%), sodium chloride (≥99%), sodium sulfate (≥99%), tetramethylthiourea (98%), phenyl isothiocyanate (98%), pyrrolidine (99%), dimethyl terephthalate (≥99.0%), triethyl orthoformate (98%), trifluoroacetic anhydride (≥99%), trifluoroacetic acid (99%), selenium powder ∼100 mesh (99.99%), and trioctylphosphine (97%) were obtained from Sigma Aldrich and used without further purification. Oleic acid (99%) and 1,1′-thiocarbonyldiimidazole (≥95.0% or 90%) were obtained from either Sigma Aldrich or Alfa Aesar and used without further purification. Diphenyl ether (99%), 1-octadecene (90%), hexadecane (99%), and tetraethylene glycol dimethyl ether (“tetraglyme” ≥99%) were obtained from Sigma Aldrich, stirred with calcium hydride overnight, and distilled prior to use. 2-Hexyldecanoic acid (96%) was obtained from Sigma Aldrich, stirred with sodium sulfate overnight, and distilled prior to use. Chloroform-*d* (99.8%), benzene-*d*_6_ (99.5%), and methylene chloride-*d*_2_ (99.8%) were obtained from Cambridge Isotopes and used without further purification. Cadmium oxide (99.99%) was obtained from Strem and used without further purification. *N*-Methylethylenediamine (95%), *N*-isopropylethylenediamine (98%), *N*,*N*′-dimethylethylenediamine (99%), *N*,*N*′-diphenyl ethylenediamine (98%), *N*,*N*′-diisopropylethylenediamine (99%), *N*-methyl-1,3-propanediamine (98%), *N*-isopropyl-1,3-propanediamine (95%), *N*,*N*′-dimethyl-1,3-propanediamine (97%), *N*,*N*′-diethyl-1,3-propanediamine (97%), and *N*,*N*′-diisopropyl-1,3-propanediamine (96%) were obtained from Sigma Aldrich and used without further purification. *N*-Ethylethylenediamine (98%), and *N*,*N*′-diethylethylenediamine (96%) were obtained from Alfa Aesar and used without further purification. *N*-Phenylethylenediamine (98% or 99%) was obtained from either Sigma Aldrich or Acros Organics and used without further purification. Column chromatography was performed with 40–63 μm silica gel (230–400 mesh).

Tetrasubstituted, trisubstituted, disubstituted thioureas, selenoureas, and thiocarbonates were prepared as described previously. *N*-Hexyl-*N*′-dodecylthiourea,^5^*N*-hexyl-*N*′*N*′-dibutylthiourea,^5^*N*-methyl-*N*,*N*′-diphenylthiourea,^7^ dipyrrolidinethiourea,^7^ and *N*-butyl-*N*′-pyrrolidineselenourea.^6^ All thiocarbonates described in the particle number *vs.* rate plot were synthesized as previously described.^7^

### Instrumentation

UV-visible absorbance spectra were obtained using a PerkinElmer Lambda 950 spectrophotometer equipped with deuterium and halogen lamps. Photoluminescence measurements were performed using a Fluoromax 4 from Horiba Scientific, and photoluminescence quantum yields were determined using a quanta-phi integrating sphere accessory according to a previously described procedure.^69^ Powder X-ray diffraction (XRD) was measured on a PANalytical X'Pert Powder X-ray diffractometer. Transmission electron microscopy (TEM) was performed on a FEI T12 BioTWIN and a FEI Talos F200X. Fourier transform infrared (FT-IR) spectra were obtained using a PerkinElmer Spectrum One FT-IR Spectrometer operating with an attenuated total reflectance (ATR) accessory.

### STEM-EDX

Elemental maps were recorded on a FEI Talos F200X operated at 80 kV and equipped with a SuperX detector. Samples were drop cast and heated to 50 °C in a vacuum oven for 1 week prior to imaging in order to assist in removal of volatile organics. The X-ray signals of a 15.8 × 15.7 nm map consisting of 233 × 231 pixels were recorded over a period of 105 minutes, during which the sample remained stable. At each pixel, a 0–20 keV spectrum was recorded over 2048 channels. From this data cube, the following slices were taken to obtain individual elemental maps: S_K_α__: 2.23–2.48 keV; Se_L_α,β__: 1.16–1.60 keV; Se_K_α__: 11.00–11.40 keV; Se_K_β__: 12.36–12.68 keV; Cd_L_β__: 3.00–3.65 keV.

### Raman spectroscopy

Nanocrystal sample thin films for Raman spectroscopy measurements are prepared by drop-casting concentrated nanocrystal solutions in hexane onto 5 × 5 mm silicon wafers. The samples are loaded into a Cryo Industries cryostat which is left under vacuum overnight before cooling the sample to 77 K over approximately an hour using a LakeShore 325 temperature controller.

The Raman spectra are measured on a home-built micro-Raman spectrometer. These measurements use 0.5–1 mW of light from an Ondax diode laser with *λ* = 406 nm. This laser light diffracts off an Ondax ASE filter to remove spontaneous emission from the laser, enters a Nikon Eclipse Ti/U inverted microscope, and is focused by a 40× /0.6 N.A. objective to a ∼1 μm diameter spot on the sample in the cryostat chamber. The backscattered light is collected by the same objective and passes through a 50 μm confocal pinhole. The light passes through two polarizers and an angle-tuned Semrock 407 nm RazorEdge long pass filter. The polarizers transmit P-polarized light with respect to the long pass filter angle, which permits measurement of low frequency vibrational modes (<300 cm^–1^). The light enters a 300 mm Acton SP2300 spectrometer with an 1800 g mm^–1^ grating before reaching a Pixis 400 CCD imaging detector. We typically measure multiple consecutive spectra using 300 to 450 second exposure times. These exposure times prevent detector saturation from fluorescence or Rayleigh scattering, which slightly transmits through the angle-tuned long pass filter. Measurements are performed on multiple spots on a sample, and consecutive spectra are measured on each spot to ensure that no sample degradation occurred. Total exposure time for a given sample is 20 to 90 minutes. We calibrate the spectra using an argon lamp at the beginning and end of a set of measurements for a given sample. Typical spectral resolution is 5 cm^–1^ based on the FWHM of the *λ* = 415.86 nm peak in the argon spectrum.

### Synthesis of cadmium oleate

Cadmium oxide (99.99%) (9.5 g, 74 mmol, 1 eq.) and acetonitrile (95 mL) are stirred at room temperature. Trifluoroacetic acid (1.6 mL, 20.9 mmol, 0.28 eq.) and trifluoroacetic anhydride (11 mL, 79 mmol, 1.1 eq.) are added slowly and stirred for one hour. The cadmium oxide fully dissolves, yielding a clear colorless solution. To a 4 L Erlenmeyer flask, oleic acid (46.7 mL, 148 mmol, 2 eq.), dichloromethane (740 mL), and triethylamine (26.3 mL, 188.7 mmol, 2.55 eq.) are added. The cadmium trifluoroacetate solution is then added dropwise to the oleic acid solution with stirring. An additional 600 mL of acetonitrile are added resulting in the formation of a white precipitate. The mixture is heated to 60 °C in order to dissolve the precipitate, and the flask is slowly cooled to room temperature and then put in a –22 °C freezer. The resulting white powder is isolated by vacuum filtration and washing with 1 L acetonitrile, being careful to thoroughly stir the slurry and break up large chunks. The product is dried under vacuum to yield a fine, fluffy white powder. Typical yields are 49 g (98%). ^1^H NMR (CDCl_3_, 400 MHz) *δ* = 0.90 (t, 6H, CH_3_), 1.23–1.40 (m, 40H, (CH_2_)_6_ and (CH_2_)_4_), 1.59 (m, 4H, COCH_2_C*H*_2_), 2.03 (m, 8H, =CHC*H*_2_), 2.33 (t, 4H, COCH_2_), 5.36 (m, 4H, =CH–); ^13^C{^1^H} NMR (CDCl_3_, 125 MHz) *δ* = 14.26 (CH_3_), 22.85 (*C*H_2_CH_3_), 26.15 (COCH_2_*C*H_2_), 27.44 (=CH*C*H_2_–), 27.46 (=CH*C*H_2_–), 29.50 (CH_2_), 29.53 (CH_2_), 29.57 (CH_2_), 29.74 (CH_2_), 29.97 (CH_2_), 30.07 (CH_2_), 32.09 (CH_2_), 35.73 (CO*C*H_2_), 129.80 (=CH–),130.10 (=CH–),184.15 (OOC); IR (liquid cell in tetrachloroethylene): 1317, 1405, 1437, 1468, 1530, 2850, 2873, 2918, 2955, 3007 cm^–1^; Anal. calcd For CdO_4_C_36_H_66_: C, 64.03; H, 9.85. Found: C, 63.72; H, 9.62.

### Synthesis of cyclic thioureas *S*-Im(H,R), *S*-Im(R_2_), *S*-Pym(H,R), and *S*-Pym(R_2_)

#### 1-Phenylimidazolidine-2-thione (***S*-Im(H,Ph)**)

1,1′-Thiocarbonyldiimidazole (1.09 g, 1.1 eq., 6.14 mmol) was dissolved in 10 mL of ethyl acetate. *N*-Phenylethylenediamine (0.760 g, 1 eq., 5.58 mmol) was added dropwise to the solution. The solution was heated to 70 °C and stirred for 1 hour. After cooling, the resulting precipitate is collected *via* filtration and washed with hexanes to yield 0.905 g (91%) of ***S*-Im(H,Ph)**. The product was further purified by recrystallization from ethyl acetate and hexanes to produce off-white crystals. Yield: 0.835 g (84%). ^1^H NMR (400 MHz, CD_2_Cl_2_): *δ* = 3.69–3.78 (m, 2H, –CH_2_), 4.14–4.24 (m, 2H, –CH_2_), 6.35 (br, 1H, NH), 7.24–7.32 (m, 1H, *p*-CH), 7.39–7.48 (m, 2H, *m*-CH), 7.58–7.65 (m, 2H, *o*-C); ^13^C{^1^H} (125 MHz, CDCl_3_): *δ* = 41.71 (5-CH_2_), 52.16 (4-CH_2_), 124.56 (*p*-CH), 126.44 (*m*-C), 128.86 (*o*-C), 140.02 (*i*-C), 182.75 (C(S)); Anal. calcd for C_9_H_10_N_2_S: C, 60.64; H, 5.65; N, 15.72. Found: C, 60.41; H, 5.41; N, 15.67. MS (ASAP) *m/z* calcd for [C_9_H_10_N_2_S + H^+^]: 179.06. Found: 179.06.

#### 1-Methylimidazolidine-2-thione (***S*-Im(H,Me)**)

*N*-Methyl-ethylenediamine (1.22 g, 1 eq., 16.4 mmol) was dissolved in 100 mL of ethyl acetate. A suspension of 1,1′-thiocarbonyldiimidazole (3.23 g, 1.1 eq., 18.1 mmol) in 100 mL of ethyl acetate was added to the diamine solution slowly over the course of 10 minutes. The mixture was allowed to stir at room temperature for 1 hour. The reaction mixture was gravity filtered to remove a red precipitate, and the filtrate was evaporated to produce an orange solid. This solid was dissolved in a minimal amount of dichloromethane, and was loaded onto a silica gel column, and eluted with ethyl acetate. The fractions were consolidated and evaporated to yield ***S*-Im(H,Me)** as white crystals. Yield: 1.346 g (71%). ^1^H NMR (400 MHz, CDCl_3_): *δ* = 3.13 (s, 3H, –CH_3_), 3.56 (t, 2H, –CH_2_), 3.71 (t, 2H, –CH_2_) 5.88 (br, 1H, NH); ^13^C{^1^H} (100 MHz, CDCl_3_): *δ* = 34.30 (–CH_3_), 41.17 (4-CH_2_), 51.19 (5-CH_2_), 184.07 (C(S)); Anal. calcd for C_4_H_8_N_2_S: C, 41.35; H, 6.94; N, 24.11. Found: C, 41.88; H, 6.88; N, 24.05. MS (ASAP) *m/z* calcd for [C_4_H_8_N_2_S + H^+^]: 117.05. Found: 117.05.

#### 1-Ethylimidazolidine-2-thione (***S*-Im(H,Et)**)

Synthesized following the same procedure as ***S*-Im(H,Me)**, instead using *N*-ethylethylenediamine. White crystals. Yield: 1.56 g (73%). ^1^H NMR (400 MHz, CDCl_3_): *δ* = 1.19 (t, 3H, –CH_3_), 3.66 (m, 6H, –CH_2_ & –*CH*_2_CH_3_), 5.83 (br, 1H, NH); ^13^C{^1^H} (100 MHz, CDCl_3_): *δ* = 12.13 (–CH_3_), 41.31 (4-CH_2_), 41.57 (*CH*_2_CH_3_), 47.90 (5-CH_2_), 182.90 (C(S)); Anal. Calcd for C_5_H_10_N_2_S: C, 46.12; H, 7.74; N, 21.51. Found: C, 45.86; H, 7.57; N, 21.29. MS (ASAP) *m*/*z* calcd for [C_5_H_10_N_2_S + H^+^]: 131.06. Found: 131.06.

#### 1-Isopropylimidazolidine-2-thione (***S*-Im(H,iPr)**)

Synthesized following the same procedure as ***S*-Im(H,Me)**, instead using *N*-isopropylethylenediamine. White crystals. Yield: 1.05 g (74%). ^1^H NMR (400 MHz, CDCl_3_): *δ* = 1.17 (d, 6H, –CH_3_), 3.59 (m, 4H, 4-CH_2_ & 5-*CH*_2_), 4.78 (m, 1H, –CH), 5.91 (br, 1H, NH); ^13^C{^1^H} (100 MHz, CDCl_3_): *δ* = 19.36 (–CH_3_), 41.52 (4-CH_2_), 42.88 (5-CH_2_), 46.92 (–CH) 182.54 (C(S)); Anal. calcd for C_6_H_12_N_2_S: C, 49.96; H, 8.39; N, 19.42. Found: C, 50.25; H, 8.18; N, 19.36. MS (ASAP) *m*/*z* Calcd for [C_6_H_12_N_2_S + H^+^]: 145.08. Found: 145.08.

#### 1,3-Diphenylimidazolidine-2-thione (***S*-Im(Ph_2_)**)

1,1′-Thiocarbonyldiimidazole (1.09 g, 1.1 eq., 6.14 mmol) was dissolved in 10 mL of ethyl acetate. *N*,*N*′-Diphenylethylenediamine (1.19 g, 1 eq., 5.58 mmol) was added dropwise to the solution. The solution was heated to 70 °C and stirred for 1 hour. After cooling, the resulting precipitate is collected *via* filtration and washed with hexanes to yield 1.20 g (85%) of ***S*-Im(Ph_2_)**. The product was further purified by recrystallization from ethyl acetate and hexanes to produce pale off-white crystals. Yield: 1.10 g (78%). ^1^H NMR (400 MHz, C_6_D_6_): *δ* = 3.07 (s, 4H, –CH_2_), 6.97–7.03 (m, 2H, *p*-CH), 7.14–7.23 (m, 4H, *m*-CH), 7.56–7.62 (m, 4H, *o*-CH); ^13^C{^1^H} (125 MHz, CDCl_3_): *δ* = 49.41 (–CH_2_), 125.47 (*m*-CH), 126.67 (*p*-CH), 128.89 (*o*-CH), 140.85 (*i*-CH), 181.27 (C(S)); Anal. calcd for C_15_H_14_N_2_S: C, 70.83; H, 5.55; N, 11.01. Found: C, 70.77; H, 5.57; N, 10.26. MS (ASAP) *m*/*z* calcd for [C_15_H_14_N_2_S + H^+^]: 255.10. Found: 255.10.

#### 1,3-Dimethylimidazolidine-2-thione (***S*-Im(Me_2_)**)

1,1′-Thiocarbonyldiimidazole (1.09 g, 1.1 eq., 6.14 mmol) was dissolved in 10 mL of ethyl acetate. *N*,*N*′-Dimethylethylenediamine (0.492 g, 1 eq., 5.58 mmol) was added dropwise to the solution, producing heat. The reaction mixture was extracted with 10% HCl (10 mL), washed with saturated NaHCO_3_ (10 mL), and then dried over Na_2_SO_4_. The resulting product was isolated and recrystallized from ethyl acetate and hexanes to produce colorless crystals of ***S*-Im(Me_2_)**. Yield 0.436 g (60%). ^1^H NMR (400 MHz, C_6_D_6_): *δ* = 2.38 (s, 4H, –CH_2_), 2.81 (s, 6H, –CH_3_); ^13^C{^1^H} (100 MHz, C_6_D_6_): *δ* = 24.51 (–CH_3_), 47.27 (–CH_2_), 184.25 (C(S)); Anal. calcd for C_5_H_10_N_2_S: C, 46.12; H, 7.74; N, 21.51. Found: C, 46.25; H, 7.73; N, 21.45. MS (ASAP) *m*/*z* calcd for [C_5_H_10_N_2_S + H^+^]: 131.06. Found: 131.06.

#### 1,3-Diethylimidazolidine-2-thione (***S*-Im(Et_2_)**)

Synthesized following the same procedure as **S-Im(Me_2_)**, instead using *N*,*N*′-diethylethylenediamine. Yield 0.517 g (59%). ^1^H NMR (500 MHz, C_6_D_6_): *δ* = 0.87 (t, 6H, –CH_3_), 2.54 (s, 4H, –CH_2_), 3.52 (q, 4H, –*CH*_2_CH_3_); ^13^C{^1^H} (125 MHz, CDCl_3_): *δ* = 12.06 (–CH_3_), 42.20 (–*CH*_2_CH_3_), 45.35 (–CH_2_), 181.82 (C(S)); Anal. calcd for C_7_H_14_N_2_S: C, 53.12; H, 8.92; N, 17.70. Found: C, 53.25; H, 8.75; N, 17.56. MS (ASAP) *m*/*z* calcd for [C_7_H_14_N_2_S + H^+^]: 159.10. Found: 159.10.

#### 1,3-Diisopropylimidazolidine-2-thione (***S*-Im(iPr_2_)**)

Synthesized following the same procedure as ***S*-Im(Me_2_)**, instead using *N*,*N*′-diisopropylethylenediamine. Yield 0.690 g (66%). ^1^H NMR (400 MHz, C_6_D_6_): *δ* = 0.89 (d, 12H, –CH_3_), 2.65 (s, 4H, –CH_2_), 5.19 (m, 2H, –CH); ^13^C{^1^H} (100 MHz, C_6_D_6_): *δ* = 18.64 (–CH_3_), 39.88 (–CH_2_), 46.35 (–CH), 182.06 (C(S)); Anal. calcd for C_9_H_18_N_2_S: C, 58.02; H, 9.74; N, 15.04. Found: C, 58.12; H, 9.52; N, 15.02. MS (ASAP) *m*/*z* calcd for [C_9_H_18_N_2_S + H^+^]: 187.13. Found: 187.13.

#### 1-Methyltetrahydropyrimidine-2(1*H*)-thione (***S*-Pym(H,Me)**)

Synthesized following the same procedure as ***S*-Im(H,Me)**, instead using *N*-methyl-1,3-propanediamine. Yield: 1.16 g (54%). ^1^H NMR (400 MHz, CDCl_3_): *δ* = 2.01 (m, 2H, –CH_2_), 3.28 (t, 2H, –CH_2_), 3.35 (t, 2H, –CH_2_), 3.39 (s, 3H, –CH_3_), 6.35 (br, 1H, NH); ^13^C{^1^H} (100 MHz, CDCl_3_): *δ* = 21.12 (–CH_2_), 40.75 (–CH_2_), 42.12 (–CH_2_), 48.35 (–CH_3_), 178.04 (C(S)); Anal. calcd for C_5_H_10_N_2_S: C, 46.12; H, 7.74; N, 21.51. Found: C, 47.37; H, 7.62; N, 20.96. MS (ASAP) *m*/*z* calcd for [C_5_H_10_N_2_S + H^+^]: 131.06. Found: 131.06.

#### 1-Isopropyltetrahydropyrimidine-2(1*H*)-thione (***S*-Pym(H,iPr)**)

Synthesized following the same procedure as ***S*-Im(H,Me)**, instead using *N*-isopropyl-1,3-propanediamine and 0.36 mmol scale. Yield: 0.0263 g (46%). ^1^H NMR (400 MHz, CDCl_3_): *δ* = 1.16 (d, 6H, CH_3_), 1.96 (m, 2H, CH_2_), 3.24 (m, 4H, CH_2_), 5.64 (m, 1H, CH), 6.25 (br, 1H, NH); ^13^C{^1^H} (100 MHz, CDCl_3_): *δ* = 19.07 (–CH_3_), 21.11 (–CH_2_), 38.99 (–CH_2_), 40.85 (–CH_2_), 51.87 (–CH), 177.05 (C(S)); Anal. calcd for C_7_H_14_N_2_S: C, 53.12; H, 8.92; N, 17.70. Found: C, 53.32; H, 8.76; N, 17.72. MS (ASAP) *m*/*z* calcd for [C_7_H_14_N_2_S + H^+^]: 159.10. Found: 159.10.

#### 1,3-Dimethyltetrahydropyrimidine-2(1*H*)-thione (***S*-Pym(Me_2_)**)

Synthesized following the same procedure as ***S*-Im(H,Me)**, instead using *N*,*N*′-dimethyl-1,3-propanediamine and a 3.6 mmol scale. Yield: 0.373 g (72%). ^1^H NMR (400 MHz, CDCl_3_): *δ* = 2.04 (m, 2H, –CH_2_), 3.38 (t, 4H, –CH_2_), 3.43 (s, 6H, –CH_3_); ^13^C{^1^H} (100 MHz, CDCl_3_): *δ* = 21.15 (–CH_2_), 43.46 (–CH_2_), 48.87 (–CH_3_) 179.38 (C(S)); Anal. calcd for C_6_H_12_N_2_S: C, 49.96; H, 8.39; N, 19.42. Found: C, 50.73; H, 8.27; N, 19.32. MS (ASAP) *m*/*z* calcd for [C_6_H_12_N_2_S + H^+^]: 145.08. Found: 145.08.

#### 1,3-Diethyltetrahydropyrimidine-2(1*H*)-thione (***S*-Pym(Et_2_)**)

Synthesized following the same procedure as ***S*-Im(H,Me)**, instead using *N*,*N*′-diethyl-1,3-propanediamine. Yield: 2.09 g (74%). ^1^H NMR (400 MHz, CDCl_3_): *δ* = 1.23 (t, 6H, –CH_3_), 1.99 (m, 2H, –CH_2_), 3.33 (t, 4H, –CH_2_), 3.97 (q, 4H, –*CH*_2_CH_3_); ^13^C{^1^H} (100 MHz, CDCl_3_): *δ* = 12.13 (–CH_3_), 21.38 (–CH_2_), 46.08 (–CH_2_), 49.86 (–*CH*_2_CH_3_), 177.59 (C(S)); Anal. calcd for C_8_H_16_N_2_S: C, 55.77; H, 9.36; N, 16.26. Found: C, 56.03; H, 9.22; N, 16.29. MS (ASAP) *m*/*z* calcd for [C_8_H_16_N_2_S + H^+^]: 173.11. Found: 173.11.

#### 1,3-Diisopropyltetrahydropyrimidine-2(1*H*)-thione (***S*-Pym(iPr_2_)**)

Synthesized following the same procedure as ***S*-Im(H,Me)**, instead using *N*,*N*′-diethyl-1,3-propanediamine and 7.64 mmol scale. Yield: 1.16 g (76%). ^1^H NMR (400 MHz, CDCl_3_): *δ* = 1.16 (d, 12H, –CH_3_), 1.91 (m, 2H, –CH_2_), 3.18 (t, 4H, –CH_2_), 5.91 (m, 2H, –CH); ^13^C{^1^H} (100 MHz, CDCl_3_): *δ* = 19.23 (–CH_3_), 21.76 (–CH_2_), 39.59 (–CH_2_), 52.19 (–CH), 177.88 (C(S)); Anal. calcd for C_10_H_20_N_2_S: C, 59.95; H, 10.06; N, 13.98. Found: C, 61.14; H, 10.11; N, 12.79. MS (ASAP) *m*/*z* calcd for [C_10_H_20_N_2_S + H^+^]: 201.14. Found: 201.14.

### Synthesis of cyclic selenoureas *Se*-Im(H,R), *Se*-Im(R_2_), *Se*-Pym(H,R), and *Se*-Pym(R_2_)

Adapting a procedure from Zhou and Denk,^56^ selenium (97.5 mmol), triethyl orthoformate (195 mmol), and the appropriate diamine (97.5 mmol) were added to a PTFE-sealable Schlenk flask equipped with a distillation apparatus. The reaction mixture was degassed by the freeze–pump–thaw method, placed under an argon atmosphere, and heated to 130 °C with stirring for 8 hours. Over this period, the selenium dissolved and a small amount of liquid condensed in the receiving flask. The reaction mixture was then allowed to cool to room temperature and the triethyl orthoformate was removed by distillation under reduced pressure. The flask was opened to air and the remaining solid residue was dissolved in dichloromethane, filtered through Celite, and recrystallized once outside of the glovebox. **Caution:** The contained should be handled in an efficient fumehood to avoid exposure to a strong odor. The resulting solid residue was brought into a nitrogen-filled glove box, where it was dissolved in acetonitrile, syringe filtered (PTFE, 0.2 μm), and then purified by recrystallization from acetonitrile in a –40 °C freezer for >2 hours. The resulting solid was isolated by suction filtration using a fritted glass funnel, and washed thoroughly with pentane, and then dried under vacuum for >6 hours.

#### 1,3-Diethylimidazolidine-2-selenone (***Se*-Im(Et_2_)**)

1,3-Diethylimidazolidine-2-selenone was prepared according to the general procedure above using *N*,*N*′-diethylethylenediamine (11.330 g, 13.97 mL, 97.5 mmol), selenium (7.699 g, 97.5 mmol), and triethyl orthoformate (28.899 g, 32.43 mL, 32.43 mmol). It is recrystallized by addition of pentane to a saturated toluene solution of the crude product. Yield: 15.3 g (76.5%) ^1^H NMR (400 MHz, CDCl_3_): *δ* = 1.17 (t, 6H, –CH_3_), 3.55 (s, 4H, –CH_2_), 3.75 (q, 4H, –*CH*_2_CH_3_); ^13^C{^1^H} (101 MHz, CDCl_3_): *δ* = 12.28 (–CH_3_), 44.16 (–*CH*_2_CH_3_), 46.37 (–CH_2_), 179.86 (C(Se)); ^77^Se {^1^H} (76 MHz, CDCl_3_): *δ* = 62.14; Anal. calcd for C_7_H_14_N_2_Se: C, 40.98; H, 6.88; N, 13.65. Found: C, 41.19; H, 6.57; N, 13.70. MS (ASAP) *m*/*z* Calcd for [C_7_H_14_N_2_Se + H^+^]: 207.04. Found: 207.04.

#### 1-Phenylimidazolidine-2-selenone (***Se*-Im(H,Ph)**)

This synthesis was performed on a 120 mmol scale. Yield: 6.85 g (25%). ^1^H NMR (400 MHz, CD_2_Cl_2_): *δ* = 3.76 (t, 2H, –CH_2_), 4.18 (t, 2H, –CH_2_), 6.66 (br, 1H, NH), 7.34 (t, 1H, *p*-CH), 7.46 (t, 2H, *m*-CH), 7.61 (d, 2H, *o*-CH); ^13^C{^1^H} (101 MHz, CD_2_Cl_2_): *δ* = 43.16 (–CH_2_), 52.91 (–CH_2_), 125.36 (*m*-CH), 126.83 (*p*-CH), 128.67 (*o*-CH), 140.64 (*i*-C), 180.32 (C(Se)); ^77^Se {^1^H} (76 MHz, CD_2_Cl_2_): *δ* = 133.52; Anal. calcd for C_9_H_10_N_2_Se: C, 48.01; H, 4.48; N, 12.44. Found: C, 47.90; H, 4.49; N, 12.37. MS (ASAP) *m*/*z* calcd for [C_9_H_10_N_2_Se + H^+^]: 227.01. Found: 227.01.

#### 1-Ethylimidazolidine-2-selenone (***Se*-Im(H,Et)**)

This synthesis was performed on a 30 mmol scale. Yield: 2.01 g (38%). ^1^H NMR (400 MHz, CDCl_3_): *δ* = 1.17 (t, 3H, –CH_3_), 3.58 (m, 2H, –CH_2_), 3.67 (m, 4H, –CH_2_), 6.83 (br, 1H, NH); ^13^C{^1^H} (101 MHz, CDCl_3_): *δ* = 12.24 (–CH_3_), 42.66 (–CH_2_), 43.51 (–CH_2_), 48.08 (–CH_2_), 179.09 (C(Se)); ^77^Se {^1^H} (76 MHz, CD_2_Cl_2_): *δ* = 67.70; Anal. calcd for C_5_H_10_N_2_Se: C, 33.91; H, 5.69; N, 15.82. Found: C, 33.98; H, 5.49; N, 15.75. MS (ASAP) *m*/*z* calcd for [C_5_H_10_N_2_Se + H^+^]: 179.01. Found: 179.01.

#### 1-Isopropylimidazolidine-2-selenone (***Se*-Im(H,iPr)**)

This synthesis was performed on a 21 mmol scale. Yield: 0.84 g (21%). ^1^H NMR (400 MHz, CDCl_3_): *δ* = 1.16 (d, 6H, –CH_3_), 3.56 (m, 4H, –CH_2_), 4.85 (m, 1H, –CH), 6.68 (br, 1H, NH); ^13^C{^1^H} (101 MHz, CDCl_3_): *δ* = 19.53 (–CH_3_), 42.79 (–CH_2_), 43.07 (–CH_2_), 49.08 (–CH), 178.60 (C(Se)); ^77^Se {^1^H} (76 MHz, CD_2_Cl_2_): *δ* = 70.24; Anal. calcd for C_6_H_12_N_2_Se: C, 37.70; H, 6.33; N, 14.66. Found: C, 37.74; H, 6.12; N, 14.64. MS (ASAP) *m*/*z* calcd for [C_6_H_12_N_2_Se + H^+^]: 193.02. Found: 193.02.

#### 1,3-Diphenylimidazolidine-2-selenone (***Se*-Im(Ph_2_)**)

This synthesis was performed on a 148 mmol scale. Yield: 19.67 g (44%). ^1^H NMR (400 MHz, CD_2_Cl_2_): *δ* = 4.15 (s, 4H, –CH_2_), 7.30–7.36 (m, 2H, *p*-CH), 7.41–7.49 (m, 4H, *m*-CH), 7.53–7.59 (m, 4H, *o*-CH); ^13^C{^1^H} (101 MHz, CD_2_Cl_2_): *δ* = 51.44 (–CH_2_), 126.96 (*m*-CH), 127.50 (*p*-CH), 129.20 (*o*-CH), 142.08 (*i*-C), 181.38 (C(Se)); ^77^Se {^1^H} (76 MHz, CD_2_Cl_2_): *δ* = 174.28; Anal. calcd for C_15_H_14_N_2_Se: C, 59.81; H, 4.68; N, 9.30. Found: C, 59.68; H, 4.58; N, 9.24. MS (ASAP) *m*/*z* calcd for [C_15_H_14_N_2_Se + H^+^]: 303.04. Found: 303.04.

#### 1,3-Di*tert*butylimidazolidine-2-selenone (***Se*-Im(*t*-Bu_2_)**)

This synthesis was performed on a 103 mmol scale. Yield: 0.81 g (3%). ^1^H NMR (400 MHz, CDCl_3_): *δ* = 1.69 (s, 18H, –CH_3_), 3.47 (s, 4H, –CH_2_); ^13^C{^1^H} (101 MHz, CDCl_3_): *δ* = 28.92 (–CH_3_), 45.71 (–CH_2_), 58.07 (–*C*(CH_3_)_3_), 179.09 (C(Se)); ^77^Se {^1^H} (76 MHz, CDCl_3_): *δ* = 274.39; Anal. calcd for C_11_H_22_N_2_Se: C, 50.57; H, 8.49; N, 10.72. Found: C, 50.66; H, 8.10; N, 10.79. MS (ASAP) *m*/*z* calcd for [C_11_H_22_N_2_Se + H^+^]: 263.10. Found: 263.10.

#### 1,3-Dimethylimidazolidine-2-selenone (***Se*-Im(Me_2_)**)

This synthesis was performed on a 168 mmol scale. Yield: 15.01 g (50%). ^1^H NMR (400 MHz, C_6_D_6_): *δ* = 2.38 (s, 4H, –CH_2_), 2.89 (s, 6H, –CH_3_); ^13^C{^1^H} (101 MHz, C_6_D_6_): *δ* = 36.62 (–CH_3_), 48.50 (–CH_2_), 183.46 (C(Se)); ^77^Se {^1^H} (76 MHz, C_6_D_6_): *δ* = 110.90; Anal. calcd for C_5_H_10_N_2_Se: C, 33.91; H, 5.69; N, 15.82. Found: C, 33.93; H, 5.47; N, 15.77. MS (ASAP) *m*/*z* calcd for [C_5_H_10_N_2_Se + H^+^]: 179.01. Found: 179.01.

#### 1,3-Diisopropylimidazolidine-2-selenone (***Se*-Im(iPr_2_)**)

This synthesis was performed on a 86 mmol scale. Yield: 11.17 g (55.9%). ^1^H NMR (400 MHz, C_6_D_6_): *δ* = 0.88 (d, 12H, –CH_3_), 2.63 (s, 4H, –CH_2_), 5.33 (m, 2H, –CH); ^13^C{^1^H} (101 MHz, C_6_D_6_): *δ* = 19.11 (–CH_3_), 41.05 (–CH_2_), 48.70 (–CH), 181.30 (C(Se)); ^77^Se {^1^H} (76 MHz, C_6_D_6_): *δ* = 107.23; Anal. calcd for C_9_H_18_N_2_Se: C, 46.35; H, 7.78; N, 12.01. Found: C, 46.41; H, 7.51; N, 12.14. MS (ASAP) *m*/*z* calcd for [C_9_H_18_N_2_Se + H^+^]: 235.07. Found: 235.07.

#### 1-Methyltetrahydropyrimidine-2(1*H*)-selenone (***Se*-Pym(H,Me)**)

This synthesis was performed on a 32 mmol scale. Yield: 0.62 g (11%). ^1^H NMR (400 MHz, CDCl_3_): *δ* = 2.07 (m, 2H, –CH_2_), 3.23 (m, 2H, –CH_2_), 3.36 (t, 2H, –CH_2_), 3.50 (s, 3H, –CH_3_), 6.70 (br, 1H, NH); ^13^C{^1^H} (101 MHz, CDCl_3_): *δ* = 20.71 (–CH_2_), 40.57 (–CH_2_), 45.08 (–CH_2_), 48.04 (–CH_3_), 173.35 (C(Se)); ^77^Se {^1^H} (76 MHz, C_6_D_6_): *δ* = 205.43; Anal. calcd for C_5_H_10_N_2_Se: C, 33.91; H, 5.69; N, 15.82. Found: C, 3.99; H, 5.48; N, 15.80. MS (ASAP) *m*/*z* calcd for [C_5_H_10_N_2_Se + H^+^]: 179.01. Found: 179.01.

#### 1,3-Dimethyltetrahydropyrimidine-2(1*H*)-selenone (***Se*-Pym(Me_2_)**)

This synthesis was performed on a 45 mmol scale. Yield: 0.69 g (8%). ^1^H NMR (400 MHz, CDCl_3_): *δ* = 2.05 (m, 2H, –CH_2_), 3.36 (t, 4H, –CH_2_), 3.55 (s, 6H, –CH_3_); ^13^C{^1^H} (101 MHz, CDCl_3_): *δ* = 20.92 (–CH_2_), 46.94 (–CH_2_), 48.46 (–CH_3_), 177.76 (C(Se)); ^77^Se {^1^H} (76 MHz, C_6_D_6_): *δ* = 221.22; Anal. calcd for C_6_H_12_N_2_Se: C, 37.70; H, 6.33; N, 14.66. Found: C, 37.72; H, 6.16; N, 14.63. MS (ASAP) *m*/*z* calcd for [C_6_H_12_N_2_Se + H^+^]: 193.02. Found: 193.02.

#### 1,3-Diethyltetrahydropyrimidine-2(1*H*)-selenone (***Se*-Pym(Et_2_)**)

This synthesis was performed on a 52 mmol scale. Yield: 0.23 g (2%). ^1^H NMR (400 MHz, CDCl_3_): *δ* = 1.24 (t, 6H, –CH_3_), 2.00 (m, 2H, –CH_2_), 3.31 (t, 4H, –CH_2_), 4.07 (q, 4H, –CH_2_); ^13^C{^1^H} (101 MHz, CDCl_3_): *δ* = 12.31 (–CH_3_), 21.06 (–CH_2_), 45.68 (–CH_2_), 53.26 (–CH_2_), 175.65 (C(Se)); ^77^Se {^1^H} (76 MHz, C_6_D_6_): *δ* = 175.18; Anal. calcd for C_8_H_16_N_2_S: C, 43.84; H, 7.36; N, 12.78. Found: C, 43.88; H, 7.31; N, 12.74. MS (ASAP) *m*/*z* calcd for [C_8_H_16_N_2_S + H^+^]: 221.06. Found: 221.06.

#### 1,3-Diisopropyltetrahydropyrimidine-2(1*H*)-selenone (***Se*-Pym(iPr_2_)**)

This synthesis was performed on a 20 mmol scale. Yield: 0.15 g (3%) ^1^H NMR (400 MHz, CDCl_3_): *δ* = 1.21 (d, 12H, –CH_3_), 1.92 (m, 2H, –CH_2_), 3.18 (t, 4H, –CH_2_), 6.08 (m, 2H, –CH); ^13^C{^1^H} (101 MHz, CDCl_3_): *δ* = 19.25 (–CH_3_), 21.41 (–CH_2_), 39.77 (–CH_2_), 56.20 (–CH), 176.12 (C(Se)); ^77^Se {^1^H} (76 MHz, C_6_D_6_): *δ* = 175.96; Anal. calcd for C_10_H_20_N_2_Se: C, 48.58; H, 8.15; N, 11.33. Found: C, 48.98; H, 7.93; N, 11.40. MS (ASAP) *m*/*z* calcd for [C_10_H_20_N_2_Se + H^+^]: 249.09. Found: 249.09.

### Synthesis of CdS, CdSe, and CdSe_1–*x*_S_*x*_ nanocrystals

In a nitrogen-filled glove box, a three-neck round bottom flask is loaded with cadmium oleate (0.18 mmol, 0.122 g), 1-octadecene (14.25 mL, 11.2 g, 44.4 mmol), and oleic acid (0.102 g, 0.114 mL, 0.36 mmol). A 4 mL vial was filled with the desired sulfur and/or selenium precursor (0.15 mmol) and diphenyl ether (0.78 mL, 0.80 g) or tetraglyme (0.75 mL, 0.75 g) and sealed with a rubber septum. Most compounds are more soluble in tetraglyme, however diphenyl ether is necessary to monitor reactions by ^1^H NMR spectroscopy in the 3–4 ppm range. The three-neck round bottom flask is transferred to a Schlenk line and heated to 240 °C under Ar. Occasionally, precursor solutions were heated or sonicated to ensure a homogenous solution prior to injection. The sulfur and/or selenium precursor solution is then injected into the cadmium oleate solution, and the reaction mixture is stirred for the appropriate time. The resulting nanocrystals were isolated from the reaction mixture by precipitation with acetone and centrifugation. The colored residue is redispersed in hexane (10 mL). Acetone (5–10 mL) is added in 0.5 mL portions to precipitate cadmium oleate as a white solid, without precipitating the nanocrystals. The suspension was centrifuged, the supernatant collected, and the nanocrystals precipitated by adding 25 mL of acetone. The nanocrystals were washed three additional times by redispersion in toluene and precipitation with methyl acetate. For specific syntheses of core/shell and alloyed nanocrystals, please refer to the ESI.†

### Nanocrystal formation kinetics *via* absorption spectra

Aliquots of approximately 0.1 mL were taken from a CdS or CdSe nanocrystal reaction and deposited into a previously weighed vial. A mass of toluene equal to 2.5× the weight of the aliquot was added to the vial to standardize aliquot concentration. UV-vis absorption spectra were taken of each aliquot and the concentration of cadmium chalcogenide in the aliquot was calculated from the size-dependent extinction coefficient at the first excitonic absorption maximum for CdS^7^ or the size-independent absorption coefficient of CdSe at 350 nm.^62^ The kinetics collected from each reaction were fit to a single exponential whose value is reported in Table 1.

### Precursor conversion kinetics *via*^1^H NMR

Quantitative aliquots of 200 μL were taken from a CdS, CdSe, or mixed precursor reaction and diluted with 300 μL of CD_2_Cl_2_ and 100 μL of a 22.4 mM solution of dimethyl terephthalate dissolved in CD_2_Cl_2_. Quantitative ^1^H NMR spectra were collected with a relaxation delay time of 30 s. Diphenyl ether must be used as the injection solvent instead of tetraglyme in order to monitor precursor disappearance kinetics between 3.0 and 4.5 ppm. Precursor disappearance was measured *versus* the dimethyl terephthalate internal standard and compared with the appearance of nanocrystals as measured using UV-vis absorbance spectroscopy.

## Conflicts of interest

There are no conflicts to declare.

## Supplementary Material

Supplementary information
