# Supplementary material for: Precursor reaction kinetics control compositional grading and size of CdSe1–xSx nanocrystal heterostructures
Source: Chem Sci. 2019 Jun 5;10(26):6539–52. doi: 10.1039/c9sc00989b (PMC6615248; doi:10.1039/c9sc00989b)
Supplement: Supplementary file 1 [file SC-010-C9SC00989B-s001.pdf]

Electronic Supplementary Material (ESI) for Chemical Science.
This journal is © The Royal Society of Chemistry 2019

Supplementary Information for:

# Precursor Reaction Kinetics Control Compositional Grading in $CdSe_{1-x}S_x$ Nanocrystal Heterostructures

*Leslie S. Hamachi,*[†] *Haoran Yang,*[‡] *Ilan Jen-La Plante,*[†] *Natalie Saenz,*[†] *Kevin Qian,*[†] *Michael P. Campos,*[†] *Gregory T. Cleveland,*[†] *Iva Rreza,*[†] *Aisha Oza,*[‖] *Willem Walravens,*[§] *Emory M. Chan,*[‡] *Zeger Hens,*[§⊥] *Andrew C. Crowther,*[*‖] *Jonathan S. Owen*[*†]

[†]Department of Chemistry, Columbia University, New York, New York 10027, United States

[‡]The Molecular Foundry, Lawrence Berkeley National Laboratory, Berkeley, CA 94720, United States

[§]Physics and Chemistry of Nanostructures Group (PCN), Ghent University, B-9000 Ghent, Belgium

[⊥]Center of Nano and Biophotonics, Ghent University, B-9000 Ghent, Belgium

[‖]Department of Chemistry, Barnard College, New York, New York 10027, United States

*Email: jso2115@columbia.edu
* Email: acrowthe@barnard.edu

**Sections:**

1) Nanocrystal Syntheses (Pages S2-S5)
2) Supplementary Figures (Pages S6-S22)
3) Raman Spectroscopy (Pages S23-S28)
4) Kinetics Modeling (Pages S29-S33)
5) NMR Spectra of Precursors (Pages S34-S55)
6) References (Pages S56)

## 1) Nanocrystal Synthesis

### CdSe/CdS Nanocrystal Synthesis

***Emission = 514 nm; FWHM: 34 nm ; PLQY: 76%***

A three-neck round bottom flask is loaded with cadmium oleate (0.9 mmol, 0.608 g), 1-octadecene (148 mL), and oleic acid (0.508 g, 1.14 mL, 1.8 mmol) and degassed on a Schlenk line. In a nitrogen-filled glovebox, a 20 mL vial was filled with *N*-butyl-*N'*-pyrrolidine selenourea (0.075 mmol, 0.0174 g), tetramethylthiourea (0.3 mmol, 0.030 g) and diphenyl ether (2.0 mL, 2.0 g) and sealed with a rubber septum. The three-neck round bottom flask is transferred to a Schlenk line and heated to 240 °C under Ar. The chalcogen precursor solution is injected into the cadmium oleate solution and allowed to stir for 2 hours. Upon completion of the reaction, 1-octadecene may be distilled off to reduce the volume and facilitate the purification. The resulting nanocrystals were isolated from the reaction mixture by precipitation and centrifugation as described above. This reaction is run at a lower concentration than standard conditions to mitigate otherwise mixing-limited reactivity of the selenourea.

***Emission = 622 nm; FWHM: 30 nm ; PLQY: 60% (Figure 2C)***

In a nitrogen-filled glove box, a three-neck round bottom flask is loaded with cadmium oleate (0.18 mmol, 0.122 g), 1-octadecene (14.25 mL), and oleic acid (0.102 g, 0.114 mL, 0.36 mmol). A 4 mL vial was filled with ***Se*-Im(*t*-Bu$_2$)** (0.05 mmol, 0.013 g), ***S*-Im(Me$_2$)** (0.1 mmol, 0.013 g) and diphenyl ether (0.75 mL, 0.75 g) and sealed with a rubber septum. The three-neck round bottom flask is transferred to a Schlenk line and heated to 240 °C under Ar. The chalcogen precursor solution is injected into the cadmium oleate solution and stirred for 8 hours. The resulting nanocrystals were isolated from the reaction mixture by precipitation and centrifugation.

### CdS/CdSe Nanocrystal Synthesis

***Emission = 472 nm;***

In a nitrogen-filled glove box, a three-neck round bottom flask is loaded with cadmium oleate (0.18 mmol, 0.122 g), 1-octadecene (14.25 mL), and oleic acid (0.102 g, 0.114 mL, 0.36 mmol). A 4 mL vial was filled with *N*-hexyl-*N'*-dodecyl thiourea (0.075 mmol, 0.025 g), ***Se*-Im(Et$_2$)** (0.075 mmol, 0.015 g) and tetraglyme (0.75 mL, 0.75 g) and sealed with a rubber septum. The three-neck round bottom flask is transferred to a Schlenk line and heated to 240 °C under Ar. The chalcogen precursor solution is injected into the cadmium

oleate solution and stirred for 1 hour. The resulting nanocrystals were isolated from the reaction mixture by precipitation and centrifugation.

***Emission = 513 nm;***
In a nitrogen-filled glove box, a three-neck round bottom flask is loaded with cadmium oleate (0.12 mmol, 0.081 g), 1-octadecene (9.5 mL), and oleic acid (0.068 g, 0.076 mL, 0.24 mmol). A 4 mL vial was filled with *N*-hexyl-*N'*-dodecyl thiourea (0.02 mmol, 0.0066 g), ***Se*-Im($Et_2$)** (0.08 mmol, 0.0164 g) and tetraglyme (0.5 mL, 0.5 g) and sealed with a rubber septum. The three-neck round bottom flask is transferred to a Schlenk line and heated to 240 °C under Ar. The chalcogen precursor solution is injected into the cadmium oleate solution and stirred for 1 hour. The resulting nanocrystals were isolated from the reaction mixture by precipitation and centrifugation.

***Emission = 554 nm;***
In a nitrogen-filled glove box, a three-neck round bottom flask is loaded with cadmium oleate (0.12 mmol, 0.081 g), 1-octadecene (9.5 mL), and oleic acid (0.068 g, 0.076 mL, 0.24 mmol). A 4 mL vial was filled with *N*-hexyl-*N'*-dodecyl thiourea (0.02 mmol, 0.0066 g), ***Se*-Im($iPr_2$)** (0.09 mmol, 0.021 g) and tetraglyme (0.5 mL, 0.5 g) and sealed with a rubber septum. The three-neck round bottom flask is transferred to a Schlenk line and heated to 240 °C under Ar. The chalcogen precursor solution is injected into the cadmium oleate solution and stirred for 9.5 hours. The resulting nanocrystals were isolated from the reaction mixture by precipitation and centrifugation.

**Alloy Nanocrystal Synthesis**

**d = 2.5 nm CdSxSe1-x Alloy Nanocrystal Synthesis**

In a nitrogen-filled glove box, a three-neck round bottom flask is loaded with cadmium oleate (0.18 mmol, 0.122 g), 1-octadecene (14.25 mL), and oleic acid (0.102 g, 0.114 mL, 0.36 mmol). A 4 mL vial was filled with *N*-methyl-*N,N'*-diphenyl thiourea (0.0375-0.1125 mmol, 0.009-0.027 g), ***Se*-Im($Ph_2$)** (0.0375-0.1125 mmol, 0.011-0.034 g) and diphenyl ether (0.75 mL, 0.75 g) and sealed with a rubber septum. The three-neck round bottom flask is transferred to a Schlenk line and heated to 240 °C under Ar. The chalcogen precursor solution is injected into the cadmium oleate solution and stirred for 10 minutes. The resulting nanocrystals were isolated from the reaction mixture by precipitation and centrifugation.

**Syringe Pump Shelling Method 1 (for products with d < 6 nm)**

**Shelling Solution Preparation:** A solution of cadmium oleate (1.4 mmol, 0.945 g), and 1-octadecene (17.75 mL) was prepared in a three-neck round bottom flask in a nitrogen-filled glovebox. The solution was transferred to a Schlenk line and heated to 100 °C to dissolve the cadmium oleate. After full dissolution, the cadmium oleate solution is cooled to room temperature. In a 20 mL vial in a nitrogen filled glovebox, tetramethylthiourea (1.4 mmol, 0.185 g) is dissolved in tetraglyme (3.51 mL, 3.51 g) and sealed with a rubber septum. The tetramethylthiourea solution is added to the cooled cadmium oleate solution and mixed well to dissolve. This mixture is indefinitely stable under argon at room temperature.

**Shelling Procedure:** A nanocrystal reaction such as CdS/CdSe with Emission = 472 nm is prepared as described above. When the reaction is nearly complete, the shelling solution is added at a rate of 1.7 mL/hr using a syringe pump. Once the desired volume of shell solution has been injected, the reaction is held at 240 °C for 30 additional minutes before cooling to room temperature.

**Syringe Pump Shelling Method 2 (for products with d > 6 nm)**

**Shelling Solution Preparation:** A solution of cadmium oleate (4.49 mmol, 3.03 g), 2-hexyldecanoic acid (4.5 mmol, 1.32 mL, 1.155 g), trioctylphosphine (2.24 mmol, 1.0 mL, 0.83 g), and 1-octadecene (41.25 mL) was prepared in a three-neck round bottom flask in a nitrogen-filled glovebox. The solution was transferred to a Schlenk line and heated to 150 °C in an oil bath to dissolve the cadmium oleate. After full dissolution, the cadmium oleate solution is cooled to room temperature. In a 20 mL vial in a nitrogen filled glovebox, tetramethylthiourea (1.5 mmol, 0.198 g) and tetraglyme (2.175 mL, 2.175 g) are combined and the vial is sealed with a rubber septum. The tetramethylthiourea solution is added to the cooled cadmium oleate solution and mixed well to dissolve.

**Shelling Procedure:** A nanocrystal reaction such as CdS/CdSe with Emission = 513 nm is prepared as described above. When the reaction is nearly complete, the shelling solution is added at a rate of 7.5 mL/hr using a syringe pump. Once the first few drops of shelling solution hit the reaction solution, the temperature is raised to 290 °C and held there for the remainder of the reaction. Note, that the syringe pump addition rate is dependent on the number of cores present in the reaction solution to avoid secondary nucleation.

**CdS/CdSe/CdS Emission: 543 nm; FWHM: 42 nm; PLQY: 53%**

**CdS/CdSe with Emission = 472** nm was prepared as described in the Supporting Information. 21.5 mL of shell precursor solution was prepared and injected via method 1 at a rate of 1.7 mL/hr keeping the temperature constant at 240 °C. Once the injection finished, the reaction was held at the reaction temperature for an additional 30 minutes before cooling. The resulting nanocrystals were isolated from the reaction mixture by precipitation and centrifugation as described above.

**CdS/CdSe/CdS Emission: 583 nm; FWHM: 46 nm; PLQY: 68%**

**CdS/CdSe with Emission = 513** nm was prepared as described in the Supporting Information but on a 2x scale. 46 mL of shell precursor solution was prepared and injected via method 2 at a rate of 7.5 mL/hr. Once the injection finished, the reaction was held at the reaction temperature for an additional 30 minutes before cooling. The resulting nanocrystals were isolated from the reaction mixture by precipitation and centrifugation as described above.

**CdS/CdSe/CdS Emission: 611 nm; FWHM: 42 nm ; PLQY: 81%**

**CdS/CdSe with Emission = 554 nm** was prepared as described in the Supporting Information. 57.5 mL of shell precursor solution was prepared and injected via method 2 at a rate of 5 mL/hr. Once the injection finished, the reaction was held at the reaction temperature for an additional 30 minutes before cooling. The resulting nanocrystals were isolated from the reaction mixture by precipitation and centrifugation as described above.

**CdS/CdSe/CdS Emission: 625 nm; FWHM: 32 nm ; PLQY: 93%**

In a nitrogen-filled glove box, a three-neck round bottom flask is loaded with cadmium oleate (0.60 mmol, 0.405 g), 1-octadecene (47.5 mL), and 2-hexyldecanoic acid (1.20 mmol, 0.308 g). A 20 mL vial was filled with *N*-hexyl-*N'*-dodecyl thiourea (0.05 mmol, 0.0164 g), ***Se*-Im(iPr$_2$)** (0.45 mmol, 0.105 g) and tetraglyme (2.5 mL, 2.5 g) and sealed with a rubber septum. The three-neck round bottom flask is transferred to a Schlenk line and heated to 240 °C under Ar. The chalcogen precursor solution is injected into the cadmium oleate solution and stirred for 9.5 hours. 10 mL of this CdS/CdSe core/shell solution was transferred into a new three-neck round bottom flask and shelled via Syringe Pump Shelling Method 2. The injection solution consisted of cadmium oleate (3.6 mmol, 2.43 g), 2-hexyldecanoic acid (3.6 mmol, 0.923 g), trioctylphosphine (1.8 mmol, 0.637 g), tetramethylthiourea (1.2 mmol, 0.159 g), octadecene (33 mL), and tetraglyme (3 mL). The solution is added at a rate of 2.0-8.0 mL/hr. Note: quartz cuvettes used for spectroscopy of aliquots must be cleaned with aqua regia between uses.

## Supplementary Figures

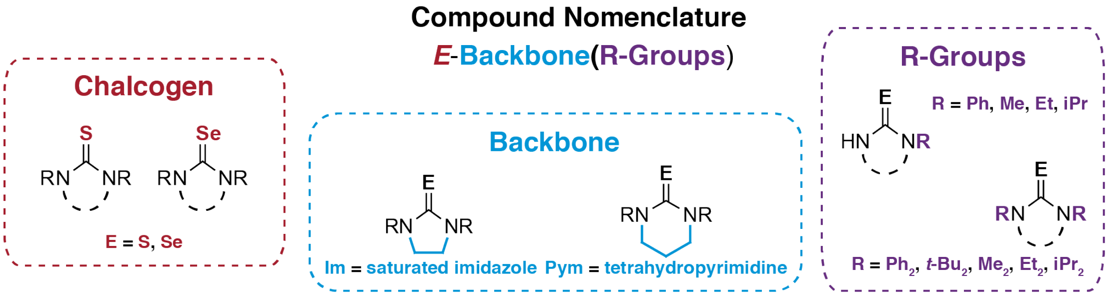


**Figure S1**. Explanation of the naming scheme for cyclic thione and selone compounds.

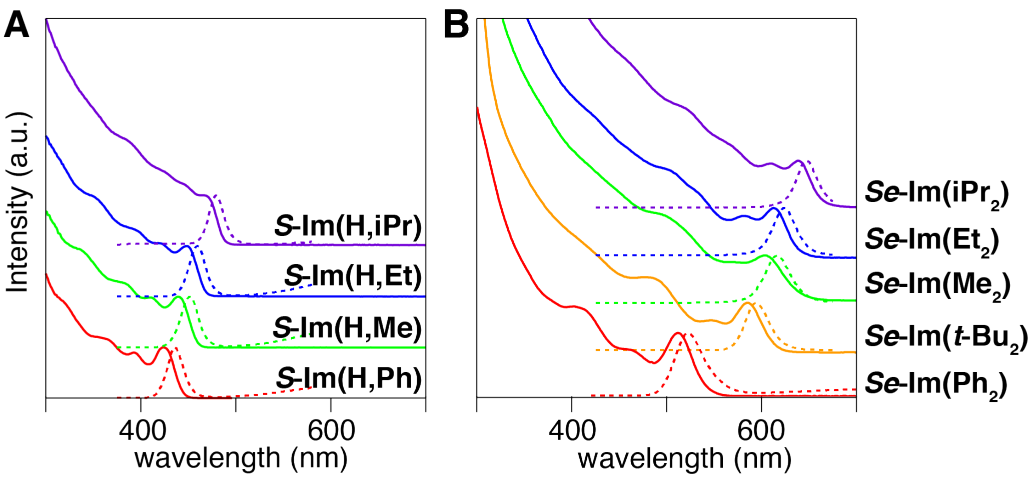


**Figure S2**. Final absorption and fluorescence spectra from reactions with several cyclic thiones and selones to produce (a) CdS nanocrystals and (b) CdSe nanocrystals.

Among the classes of new precursors, some cyclic thiones proved relatively unreactive and produce few nanocrystals that quickly reach a size that precipitates from the reaction solution. In those cases, the conversion was monitored in the presence of CdS seeds at a concentration that maintains a small nanocrystal size and a homogeneous dispersion. Classes ***S*-Im(H,R)**, ***Se*-Im(H,R), *Se*-Im($R_2$), *Se*-Pym(H,R), and *Se*-Pym($R_2$)** however, can be used to nucleate nanocrystals at desirable concentrations.

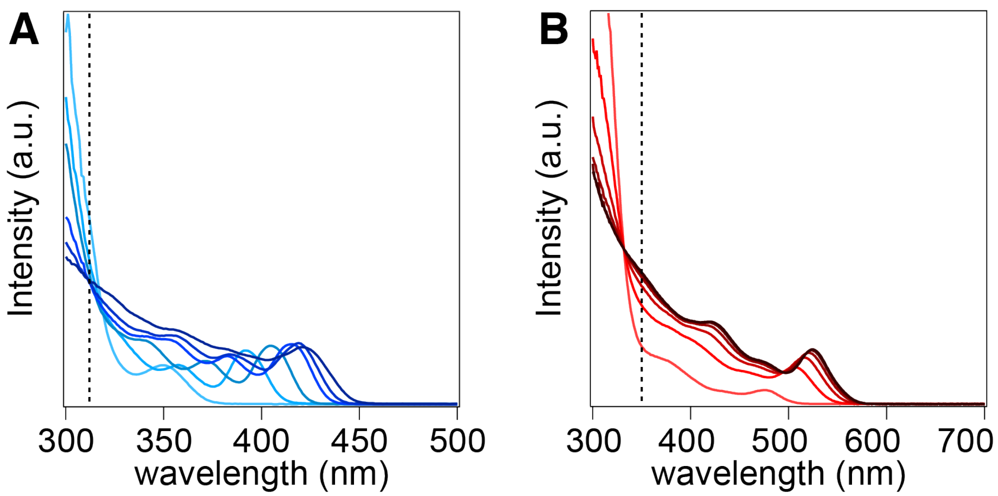


Figure S3. (a) UV-vis spectra of CdS reaction aliquots in Fig. 1B. The dashed line is at 312 nm where CdS magic sized clusters would appear. (b) UV-vis spectra of CdSe reaction aliquots in Fig. 1C. The dashed line is at 350 nm, where reaction yield was measured.

Here, we have plotted all of the UV-vis spectra from the reactions described in Figure 1B and 1C. It was suggested by a reviewer that the slight deviation above 100% total sulfur/selenium could be due to the presence of magic sized cluster intermediates. While we cannot definitively rule out the presence of a minute amount of cluster intermediates, we cannot observe any cluster formation via analysis of the UV-vis spectra and believe that their affect in these reactions is negligible.

These CdS spectra (on the left), use the first excitonic transition to determine yield. These spectra do not exhibit a sharp absorption peak at 312 nm where CdS clusters would appear.[1] The high energy absorbance above 300 nm is due to absorbance of the precursor ***S*-Im(Ph$_2$)**. It is worth noting that kinetics for this compound were run with *N*-hexyl, *N*'-dodecythiourea comprising 25% of the injected sulfur precursor that instantaneously nucleates CdS. On its own, ***S*-Im(Ph$_2$)** exhibits similar initial kinetics, but due to the relationship between slow reaction rate and large particle size, the particles eventually crash out of solution rendering analysis difficult.

The CdSe spectra (on the right) use the absorbance at 350 nm to determine yield. These spectra do not exhibit sharp CdSe cluster peaks at 393 nm, 463 nm, or 513 nm.[2] The high energy absorbance above 300 nm comes from the absorbance of the precursor ***Se*-Im(Ph$_2$)**. Yield was determined at 350 nm which does not appear to overlap with this precursor compound nor cluster peaks. Additionally, for both reactions, the urea reaction coproduct appearance via $^1$H NMR (open circles in Fig. 1B and 1C) track well with the appearance of CdE as determined via UV-vis so we believe it is unlikely that in this case, the CdE yield is affected by cluster formation.

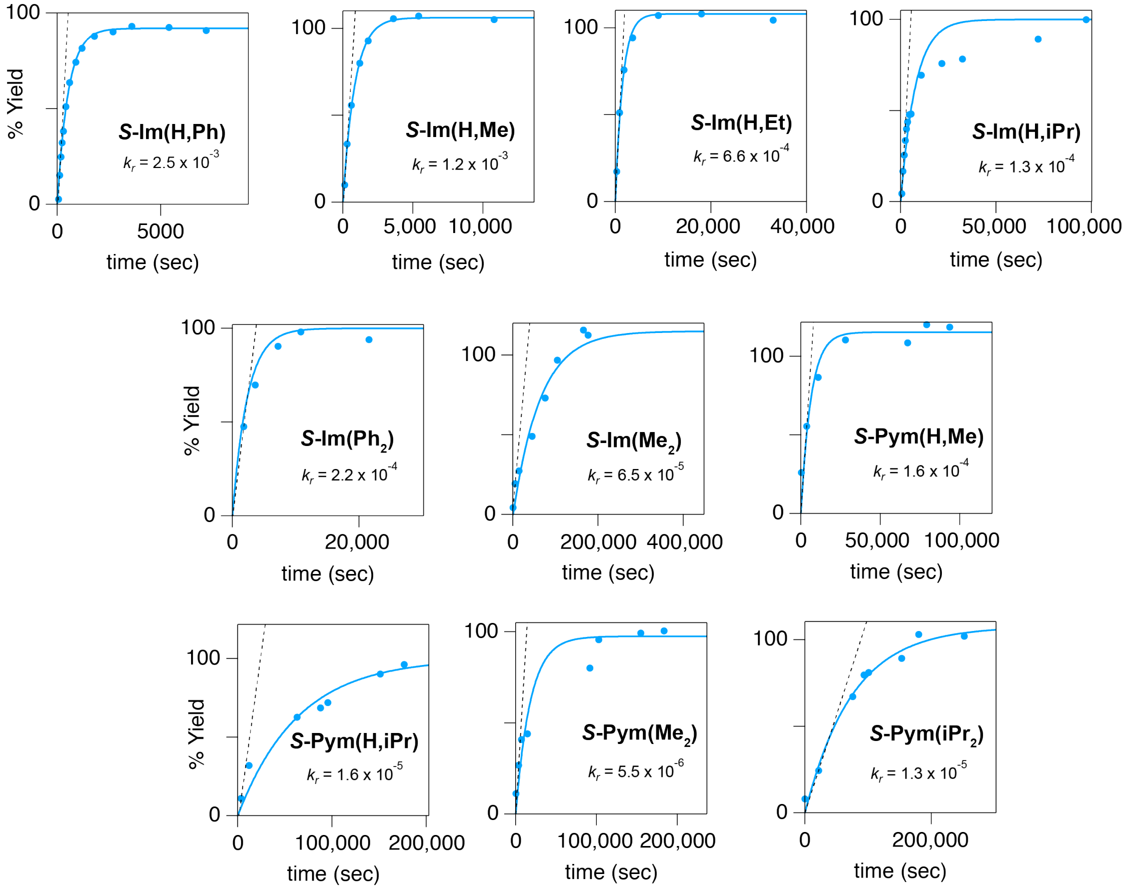


**Figure S4.** Kinetics data points for the reaction of cyclic thione precursors measured via yield of CdS nanocrystals are plotted and fit to a first order fit (light blue) and an initial rate (dashed line). Both of these values are reported in Table 1.

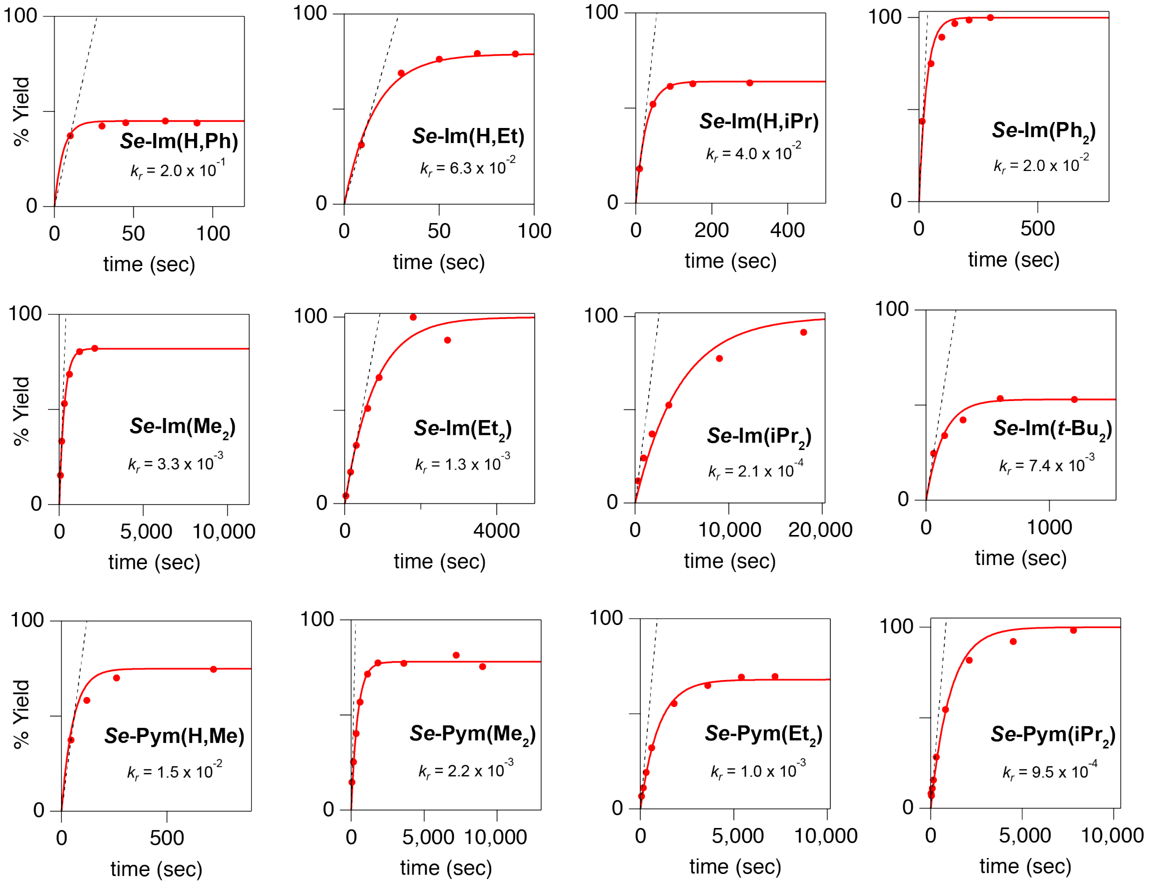


**Figure S5**. Kinetics data points for the reaction of cyclic selenone precursors measured via yield of CdSe nanocrystals are plotted and fit to a first order fit (red) and an initial rate (dashed line). Both of these values are reported in Table 1.

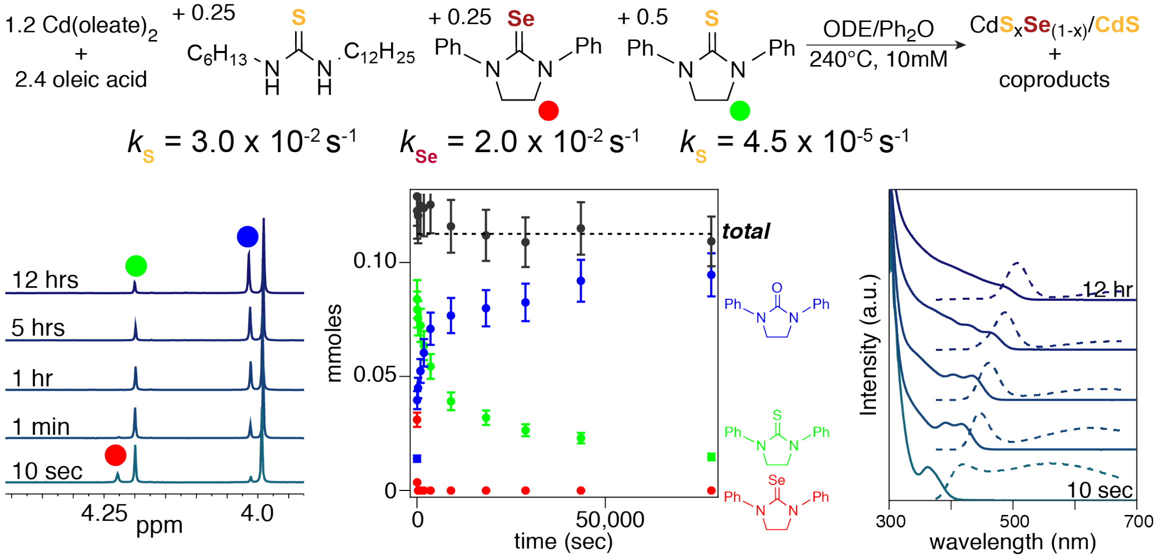


**Figure S6**. Reaction to form $CdSe_{1-x}S_x$/CdS with a single injection of three precursors, $^1$H NMR kinetics of precursor conversion, quantification of conversion via $^1$H NMR, and UV-vis and fluorescence spectra of reaction aliquots. Observed rate constants as determined via $^1$H NMR match well with single component precursor conversion rates.

### $CdS_{0.5}Se_{0.5}$/CdS Alloy/Shell Nanocrystal Synthesis

In a nitrogen-filled glove box, a three-neck round bottom flask is loaded with cadmium oleate (0.18 mmol, 0.122 g), 1-octadecene (14.25 mL), and oleic acid (0.102 g, 0.114 mL, 0.36 mmol). A 4 mL vial was filled with *N*-hexyl-*N'*-dodecyl thiourea (0.0375 mmol, 0.009 g), ***Se*-Im(Ph₂)** (0.0375 mmol, 0.011 g), ***S*-Im(Ph₂)** (0.075 mmol, 0.019 g) and diphenyl ether (0.75 mL, 0.75 g) and sealed with a rubber septum. The three-neck round bottom flask is transferred to a Schlenk line and heated to 240 °C under Ar. The chalcogen precursor solution is injected into the cadmium oleate solution and reacted for 12 hours. The resulting nanocrystals were isolated from the reaction mixture by precipitation and centrifugation and the product is described in Figure S6.

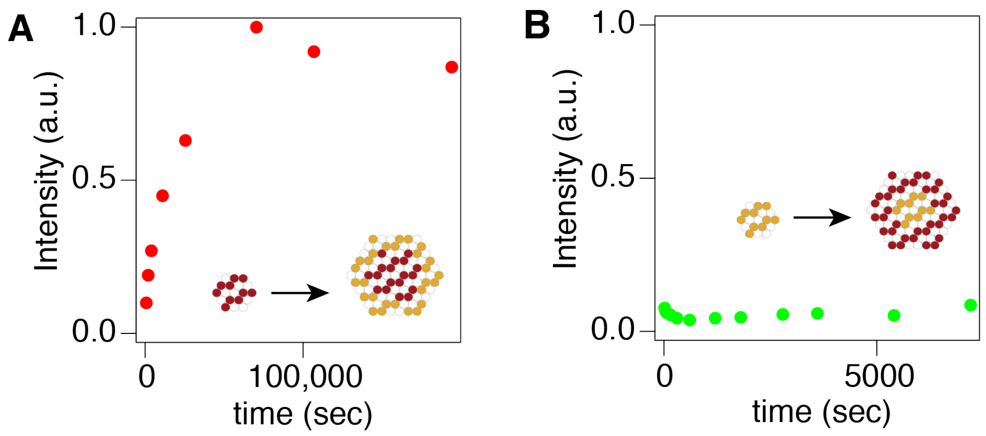



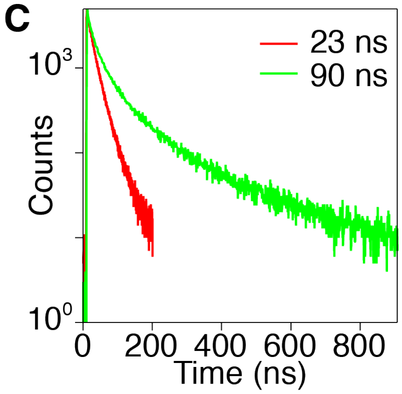



D

| Precursors | | $\tau_{1/2}$ | PLQY |
|---|---|---|---|
| 0.33 eq.<br>0.66 eq. | *Se*-Im(*t*-Bu$_2$)<br>*S*-Im(Me$_2$) | 23 ns | 60% |
| 0.5 eq.<br>0.5 eq. | *N*-hexyl-*N'*-dodecyl thiourea<br>*Se*-Im(Et$_2$) | 90 ns | 18% |

**Figure S7**. Normalized photoluminescence intensity of (a) a CdSe/CdS core/shell reaction and (b) CdS/CdSe core/shell reaction. (c) Photoluminescence lifetimes and (d) PLQY of CdSe/CdS and CdS/CdSe core/shell nanoparticles are indicative of the predicted structures.

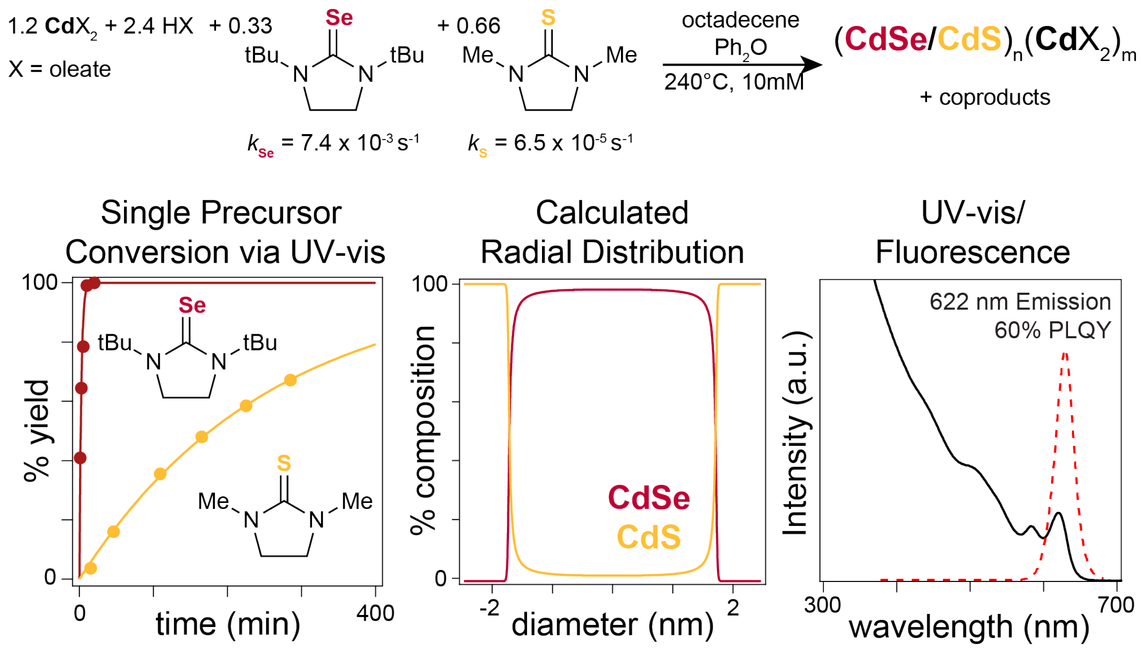


**Figure S8**. Scheme for the synthesis of red emitting CdSe/CdS nanocrystals from Figure 2C, their individual precursor conversion rates as monitored via UV-vis absorbance spectra and predicted radial distribution.

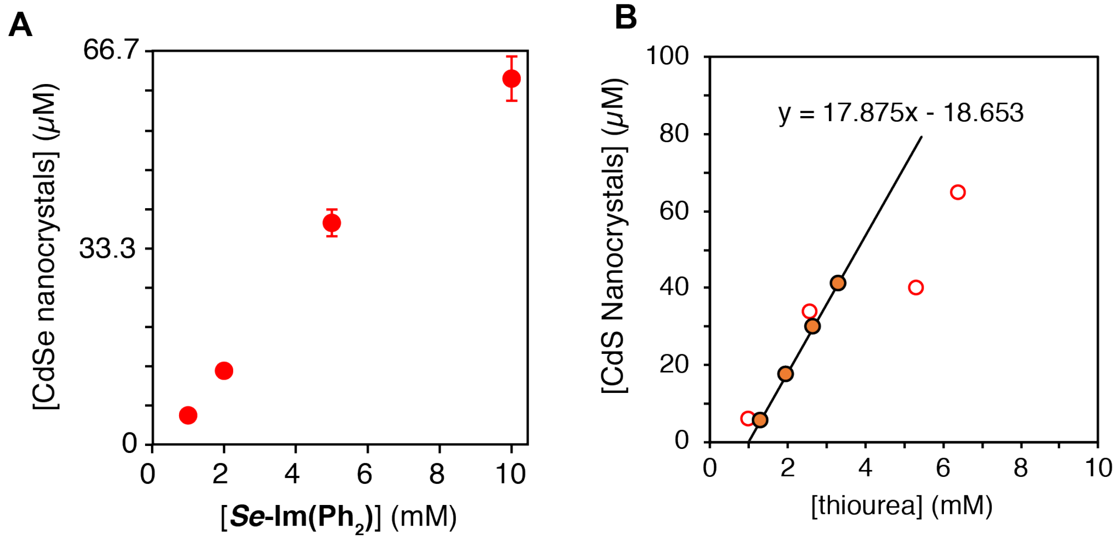


**Figure S9**. Precursor concentration dependence on concentration of nanocrystals for (a) ***Se*-Im(Ph₂)** and (b) *N*-hexyl-*N'*-dodecylthiourea (open red circles) and *N*-hexyl-*N',N'*-dibutylthiourea (orange circles). Reactions using *N*-hexyl-*N',N'*-dibutylthiourea were monitored over time and showed particle agglomeration at long reaction times, The nanoparticle concentration obtained at three minutes was near the maximum in all cases and is plotted in B above.

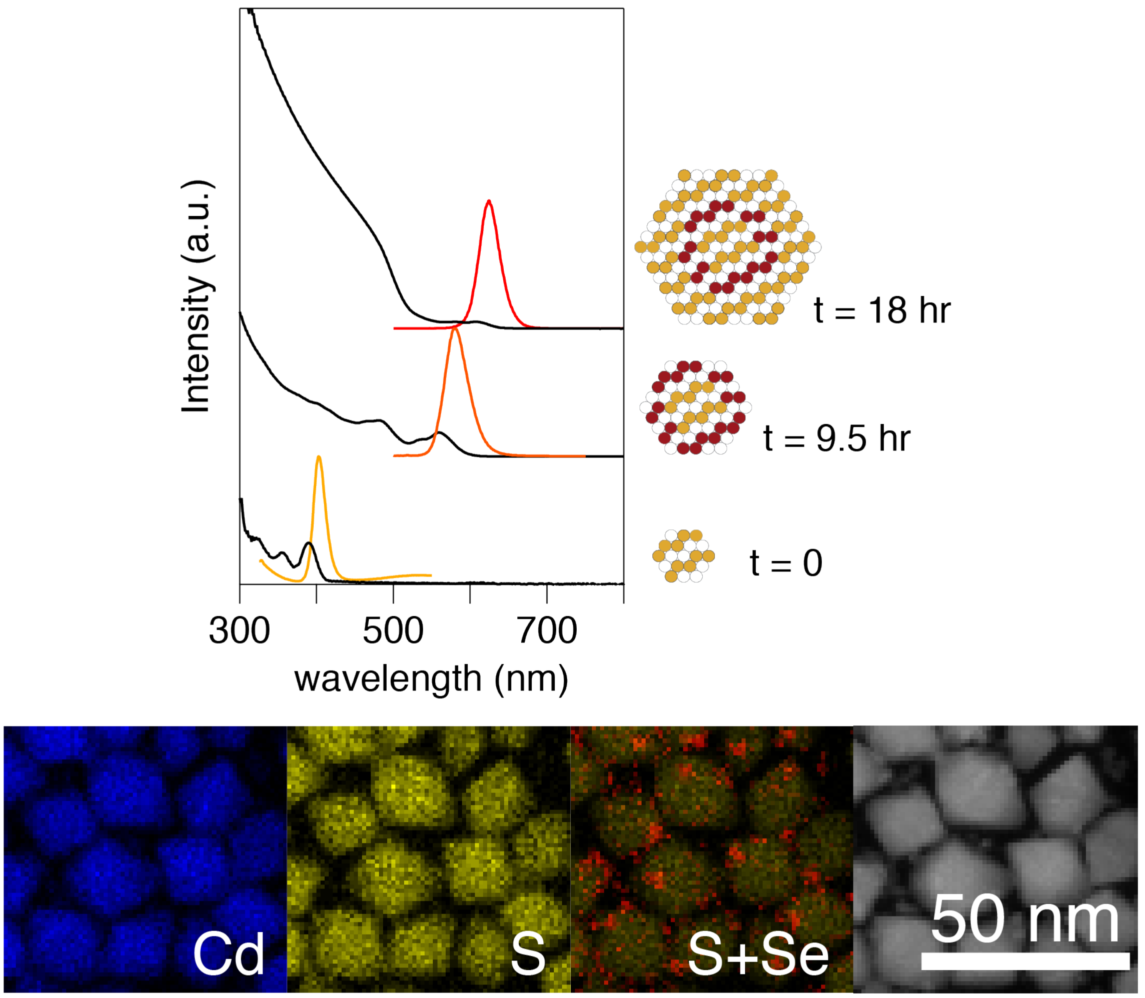

**Figure S10**. Reaction aliquots from the synthesis of a CdS/CdSe/CdS SQW reported in Figure 3 and STEM EDX maps of Cd, S and Se distributions in the particle.

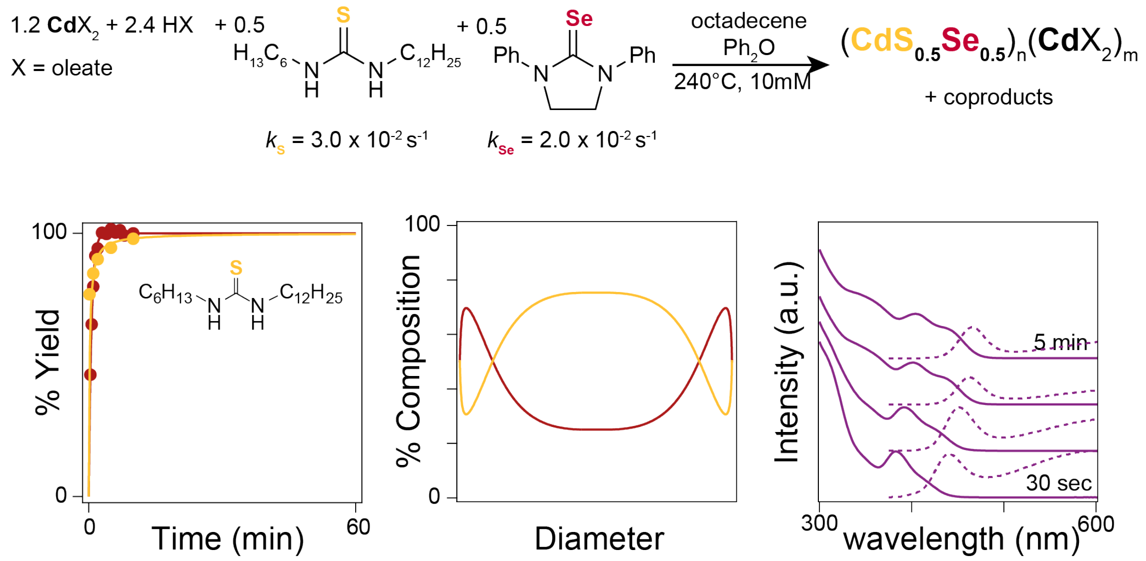


**Figure S11**. Scheme for the synthesis of blue emitting $CdS_xSe_{1-x}$ nanocrystals described in the Alloyed Nanocrystal Synthesis section, their individual precursor conversion rates as monitored via UV-vis absorbance spectra, predicted radial distribution, and UV-vis and fluorescence spectra of reaction aliquots

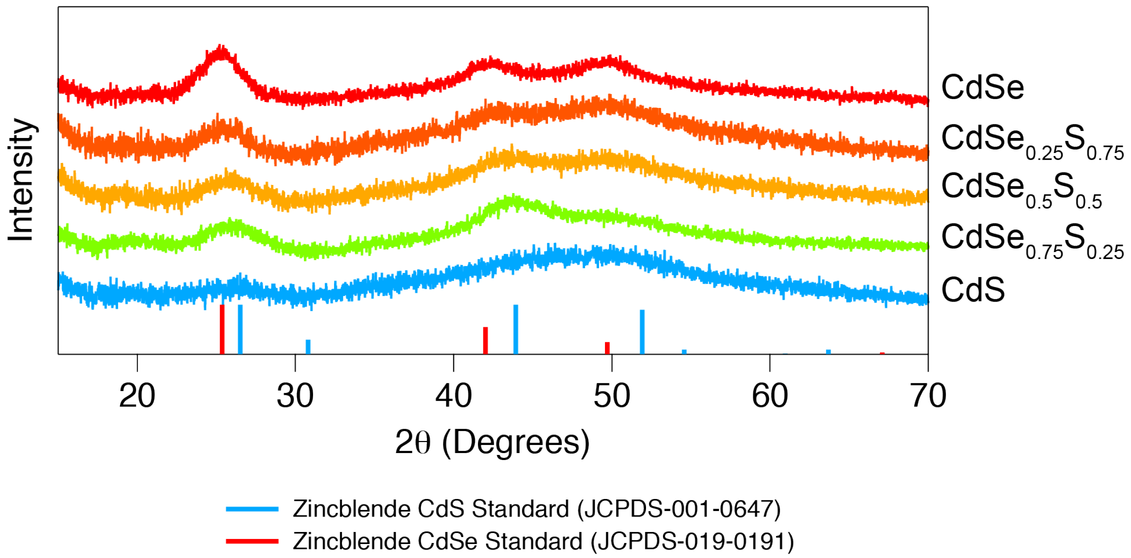


**Figure S12**. Powder X-Ray diffraction of $CdSe_{1-x}S_x$ nanocrystals synthesized as described in the Alloyed Nanocrystal Synthesis section and Figure S9.

**Table S1**. STEM EDX elemental characterization of $CdSe_{1-x}S_x$ nanocrystals synthesized as described in the Alloyed Nanocrystal Synthesis section and Figure S11. Errors bars are the relative standard deviation as estimated by Esprit 2 MicroAnalysis software.

| Sample | S (predicted atomic %) | Se (predicted atomic %) | Cd (expt. atomic %) | S (expt. atomic %) | Se (expt. atomic %) |
|---|---|---|---|---|---|
| $CdSe_{0.25}S_{0.75}$ | 30 | 10 | 61.9 ± 10.1 | 27.9 ±3.6 | 10.2 ± 4.7 |
| $CdSe_{0.5}S_{0.5}$ | 20 | 20 | 62.0 ± 10.1 | 21.5 ± 3.6 | 16.5 ± 3.9 |
| $CdSe_{0.75}S_{0.25}$ | 10 | 30 | 62.9 ± 10.0 | 10.0 ± 3.8 | 27.2 ± 3.4 |

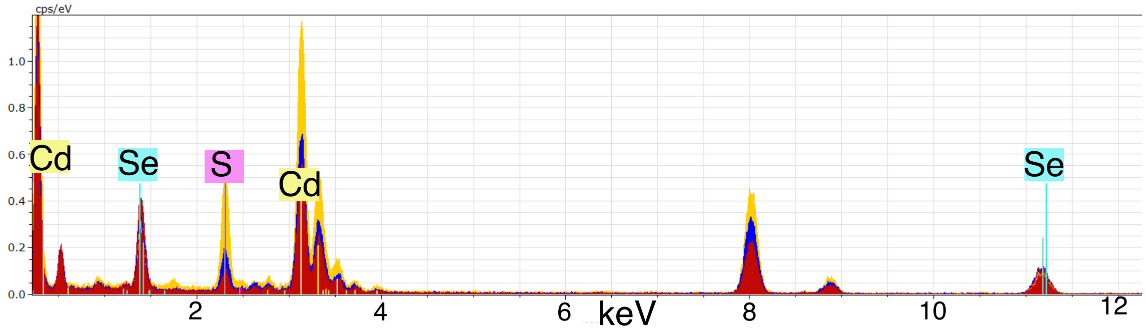


**Figure S13.** STEM-EDX of $CdSe_{1-x}S_x$ nanocrystals synthesized as described in the Alloyed Nanocrystal Synthesis section and Figure S11. (yellow) $CdSe_{0.25}S_{0.75,}$ (blue) $CdSe_{0.5}S_{0.5}$ , (red) $Se_{0.75}S_{0.25}$. TEM grids were loaded with concentrated solutions and EDX were acquired for 3 minutes.

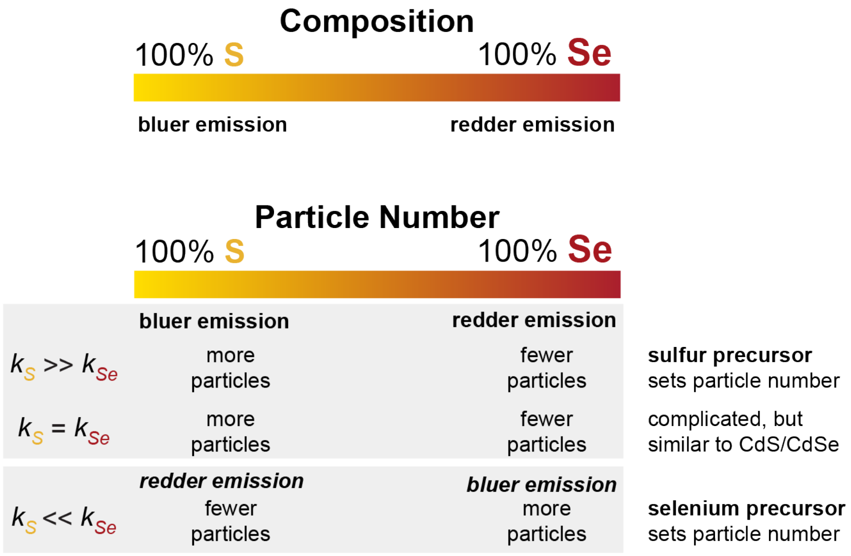


**Figure S14**. Effect of S:Se injection ratio.

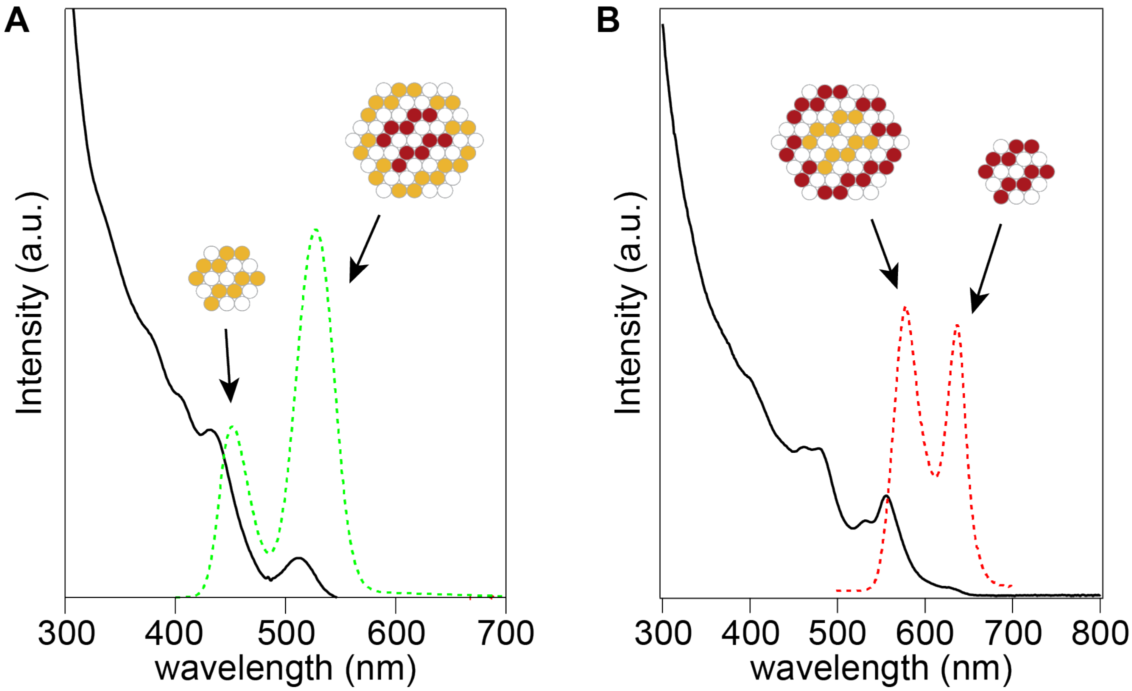


**Figure S15**. Secondary nucleation is sometimes observed. Example UV-vis and fluorescence spectra of observed secondary nucleation of shell material in (a) CdSe/CdS and (b) CdS/CdSe one-pot core/shell reactions.

**Example CdSe/CdS Second Nucleation Conditions (Figure S15A)**

In a nitrogen-filled glove box, a three-neck round bottom flask is loaded with cadmium oleate (0.09 mmol, 0.061 g), octadecene (14.25 mL), and oleic acid (0.050 g, 0.057 mL, 0.18 mmol). A 4 mL vial was filled with *N*-butyl-*N'*-pyrrolidine selenourea (0.0075 mmol, 0.0017 g), tetramethylthiourea or ***S*-Im(Me$_2$)** (0.03-0.0675 mmol) and diphenyl ether (0.75 mL, 0.75 g) and sealed with a rubber septum. The three-neck round bottom flask is transferred to a Schlenk line and heated to 240 °C under Ar. The chalcogen precursor solution is injected into the cadmium oleate solution and reacted for 2-5 hours. The resulting nanocrystals were isolated from the reaction mixture by precipitation and centrifugation as described in the experimental.

**Example CdS/CdSe Second Nucleation Conditions (Figure S15B)**

In a nitrogen-filled glove box, a three-neck round bottom flask is loaded with cadmium oleate (0.06 mmol, 0.041 g), octadecene (4.75 mL), and oleic acid (0.034 g, 0.038 mL, 0.12 mmol). A 4 mL vial was filled with *N*-hexyl-*N'*-dodecyl thiourea (0.005 mmol, 0.0016 g), ***Se*-Im(iPr$_2$)** (0.045 mmol, 0.0105 g) and diphenyl ether (2.5 mL, 2.5 g) and sealed with a rubber septum. The three-neck round bottom flask is transferred to a Schlenk line and heated to 240 °C under Ar. The chalcogen precursor solution is injected into the cadmium oleate solution and reacted for 9.5 hours. The resulting nanocrystals were isolated from the reaction mixture by precipitation and centrifugation as described above.

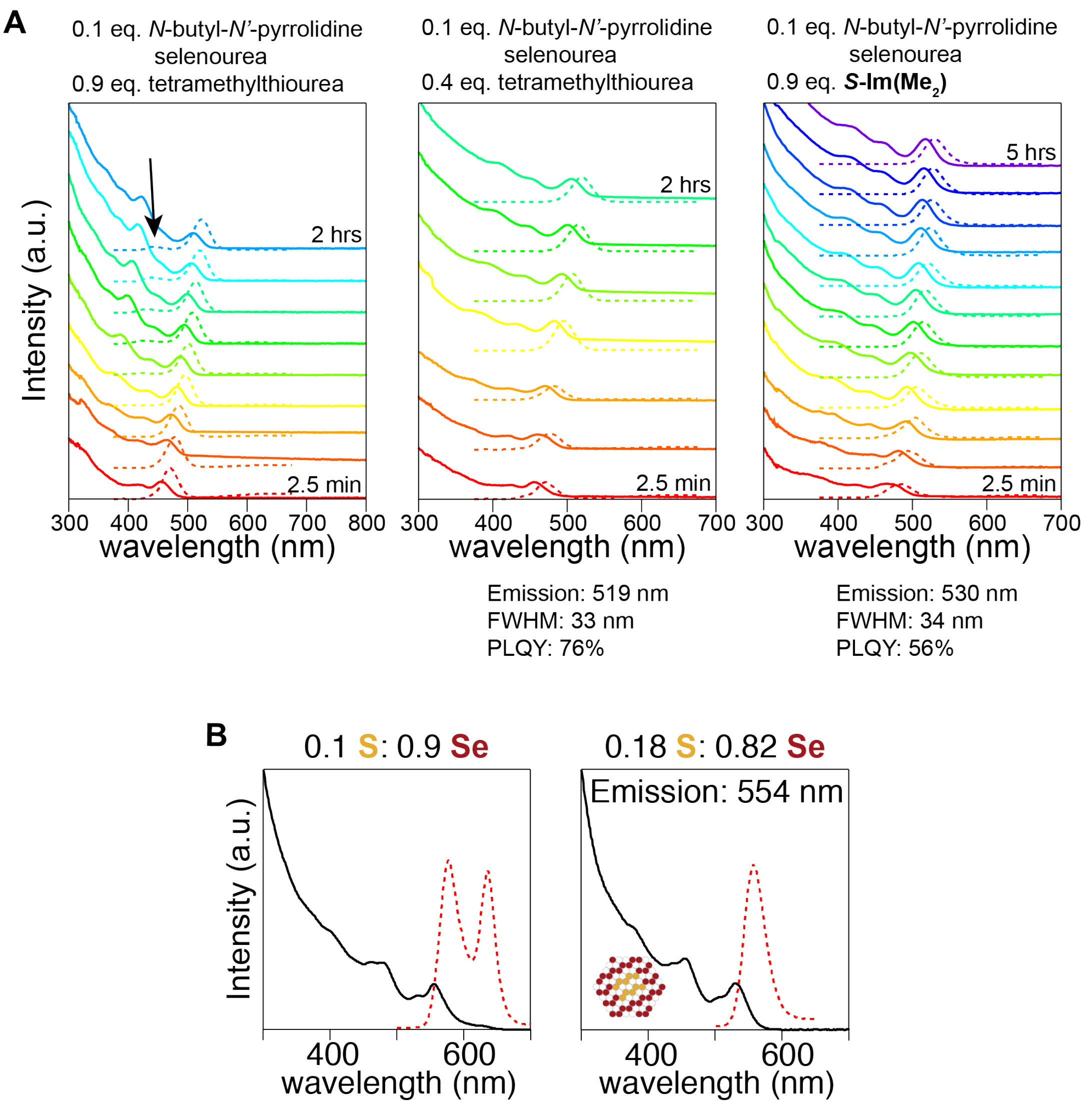


**Figure S16**. Secondary nucleation can be avoided. UV-vis and fluorescence data of (a) growing a CdS shell on CdSe nuclei results in a second population of CdS nanocrystals for 0.9 eq. of tetramethylthiourea. To reduce the appearance of the second population, add less of tetramethyl thiourea (0.4 eq.) or swap to a slower precursor ***S*-Im(Me$_2$)**. (b) UV-vis and fluorescence data of a reaction where CdSe nucleates when sulfur:selenium ratios are 0.1:0.9. By changing to a higher ratio, secondary nucleation is eliminated.

We demonstrate suppression of CdS nucleation in a typical CdSe/CdS core/shell system. When 0.1 eq. of *N*-butyl-*N'*-pyrrolidine selenourea is combined with 0.9 eq. of tetramethylthiourea, there is nucleation of a second population of CdS nanocrystals (Figure S16A). Because solute supply rate depends on [thiourea]*$k_r$, lowering the solute supply rate can be accomplished by decreasing the concentration of tetramethylthiourea to 0.4 eq. OR switching to a precursor with a slower $k_r$ such as ***S*-Im(Me$_2$)**. Both of these methods are successful in eliminating the nucleation of a second population (Figure S16A). Both of these changes do impact the final nanocrystal product; we observe that adding less eq. of tetramethylthiourea leads to a blue shifted spectrum because the CdS shell is thinner. Additionally, the reaction with ***S*-Im(Me$_2$)** also takes longer to run to

completion due to slower kinetics. Nevertheless, this example successfully illustrates how to avoid nucleation of shell precursors by considering solute supply rate.

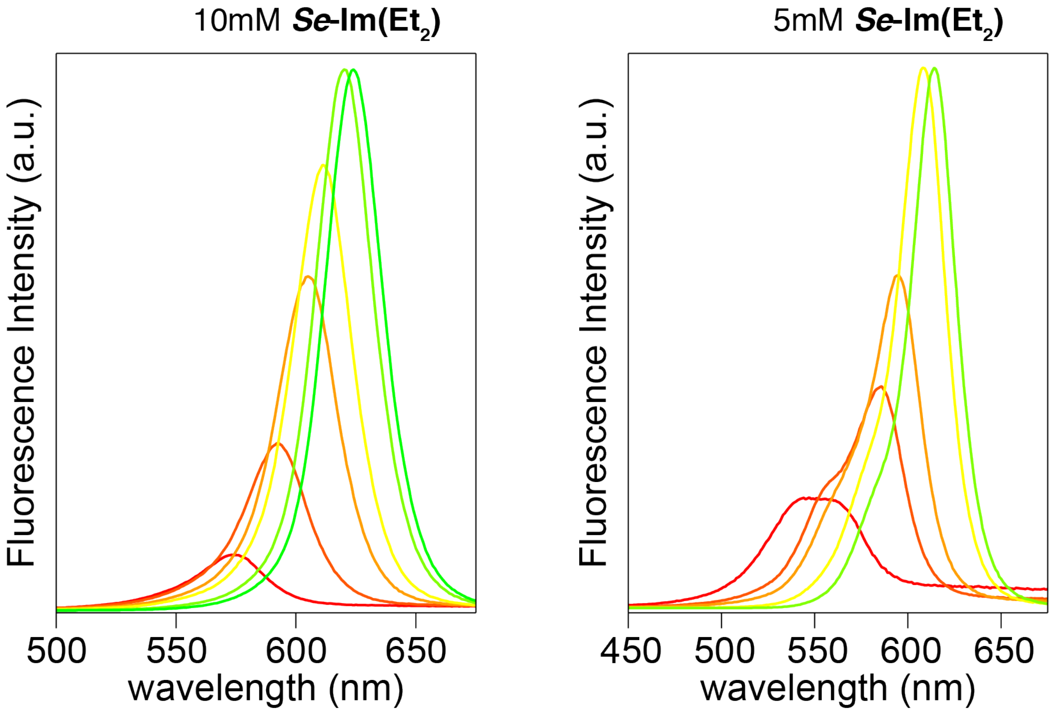


**Figure S17**. Fluorescence spectra of a CdSe nanocrystal reaction shows one population of emitters for a selone run at 10mM, whereas 5mM in selone results in the presence of two emitting populations.

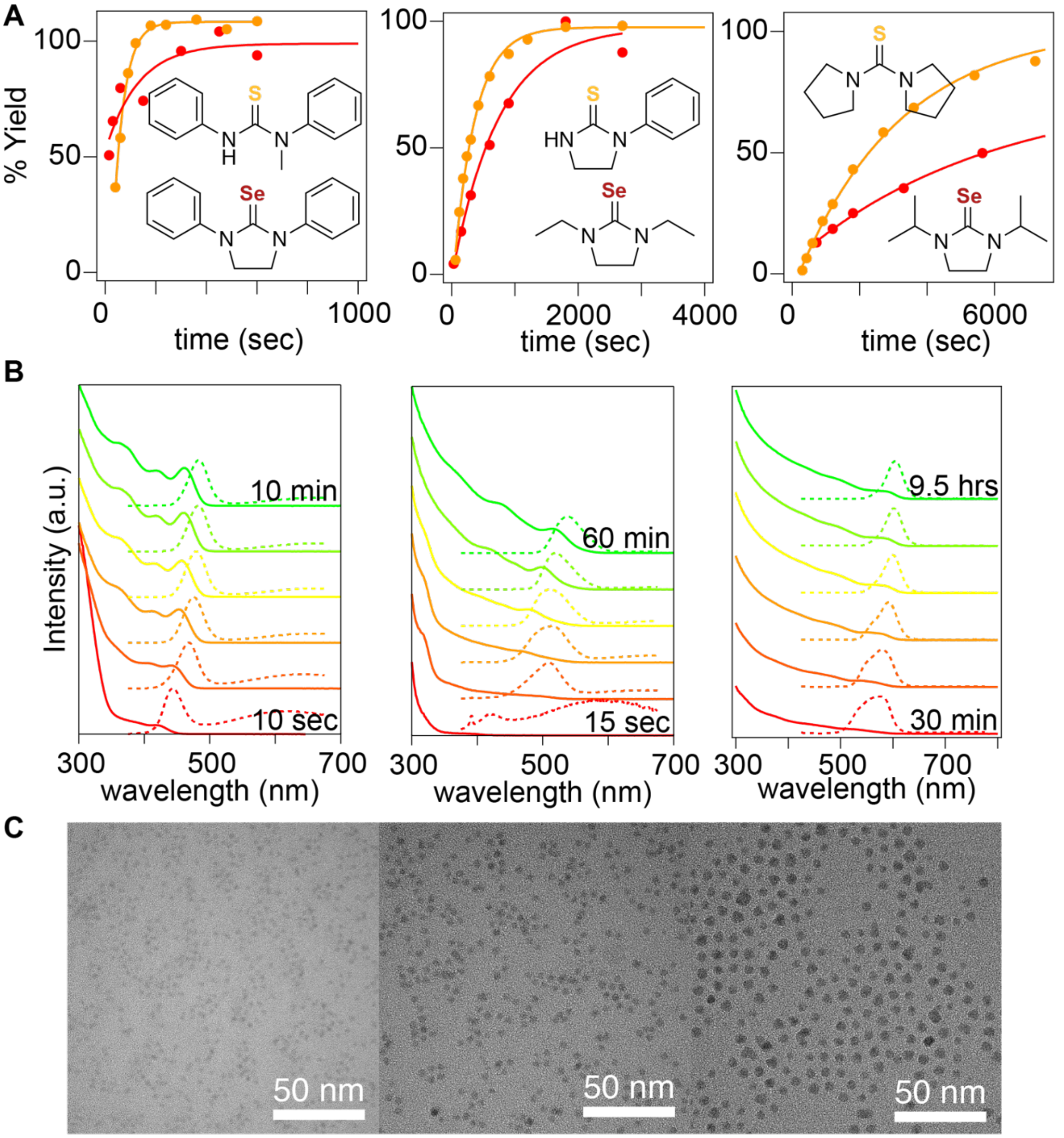


**Figure S18**. Monodisperse alloys become hard to grow with slower precursors. (a) Precursor conversion rates for three matched pairs of precursors from fast to slow as you go from left to right. (b) Resulting absorbance and fluorescence spectra of reaction aliquots. (c) Size control of $CdS_{0.5}Se_{0.5}$ alloyed nanocrystals shows increasing anisotropic growth for slower matched pairs of sulfide and selenide precursors as you move from left to right. It was suggested by a reviewer that the reason for this increase in size distribution might be due to a transition from a diffusion-limited to reaction-limited nanocrystal growth regime.

**$d$ = 3.7 nm $CdS_{0.5}Se_{0.5}$ Alloy Synthesis (Figure S18)**
In a nitrogen-filled glove box, a three-neck round bottom flask is loaded with cadmium oleate (0.18 mmol, 0.122 g), octadecene (14.25 mL), and oleic acid (0.102 g, 0.114 mL, 0.36 mmol). A 4 mL vial was filled with ***S*-Im(H,Ph)** (0.075 mmol, 0.013 g), ***Se*-Im($Et_2$)** (0.075 mmol, 0.015 g) and diphenyl ether (0.75 mL, 0.75 g) and sealed with a rubber septum. The three-neck round bottom flask is transferred to a Schlenk line and heated to 240 °C under Ar. The chalcogen precursor solution is injected into the cadmium oleate solution and reacted for 1 hour. The resulting nanocrystals were isolated from the reaction mixture by precipitation and centrifugation as described in the experimental.

**$d$ = 5.7 nm $CdS_{0.5}Se_{0.5}$ Alloy Synthesis (Figure S18)**
In a nitrogen-filled glove box, a three-neck round bottom flask is loaded with cadmium oleate (0.18 mmol, 0.122 g), octadecene (14.25 mL), and oleic acid (0.102 g, 0.114 mL, 0.36 mmol). A 4 mL vial was filled with dipyrrolinethiourea (0.075 mmol, 0.014 g), ***Se*-Im(i$Pr_2$)** (0.075 mmol, 0.017 g) and diphenyl ether (0.75 mL, 0.75 g) and sealed with a rubber septum. The three-neck round bottom flask is transferred to a Schlenk line and heated to 240 °C under Ar. The chalcogen precursor solution is injected into the cadmium oleate solution and reacted for 9.5 hours. The resulting nanocrystals were isolated from the reaction mixture by precipitation and centrifugation as described in the experimental.

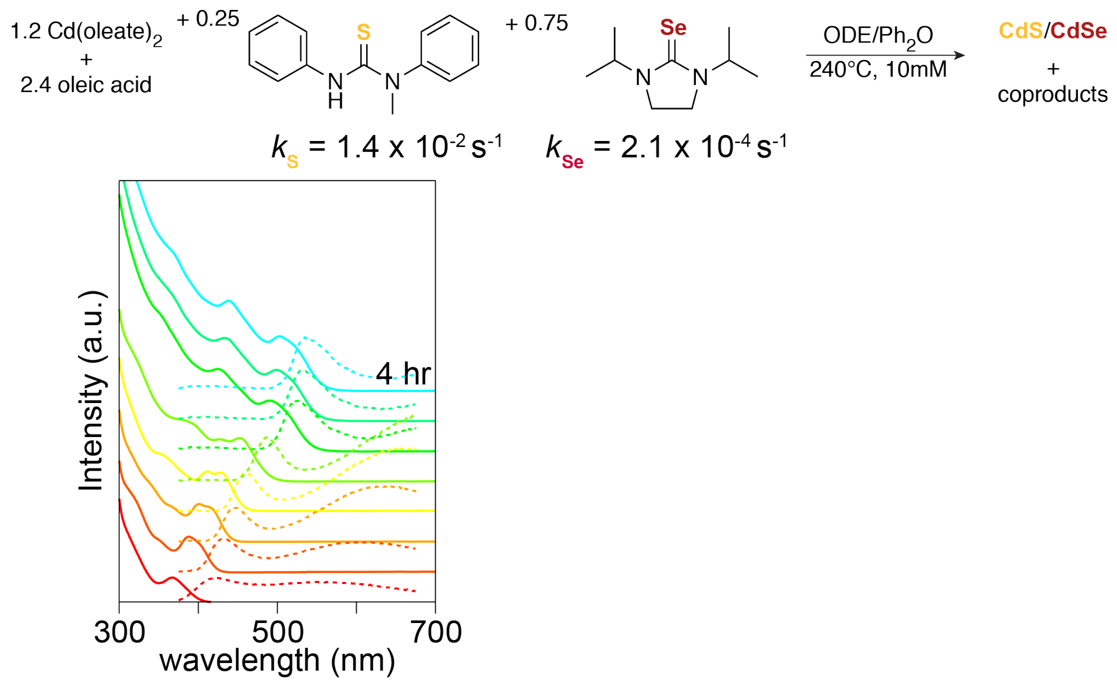


**Figure S19**. Monodisperse CdS/CdSe become hard to grow with slower precursors. Reaction scheme and UV-vis/fluorescence spectra of a reaction to make medium sized CdS/CdSe core/shell nanocrystals shows broad absorbance and fluorescence spectra.

**CdS/CdSe With Larger CdS Core (Figure S19)**

In a nitrogen-filled glove box, a three-neck round bottom flask is loaded with cadmium oleate (0.18 mmol, 0.122 g), octadecene (14.25 mL), and oleic acid (0.102 g, 0.114 mL, 0.36 mmol). A 4 mL vial was filled with *N*-methyl-*N,N'*-diphenylthiourea (0.0375 mmol, 0.009 g), ***Se*-Im(iPr$_2$)** (0.1125 mmol, 0.026 g) and diphenyl ether (0.75 mL, 0.75 g) and sealed with a rubber septum. *N*-methyl-*N,N'*-diphenyl thiourea has slower conversion kinetics than *N*-hexyl-*N'*-dodecyl thiourea which is typically used for CdS/CdSe core/shell reactions and should nucleate a CdS larger core. The three-neck round bottom flask is transferred to a Schlenk line and heated to 240 °C under Ar. The chalcogen precursor solution is injected into the cadmium oleate solution and reacted for 9.5 hours. The resulting nanocrystals were isolated from the reaction mixture by precipitation and centrifugation as described in the experimental.

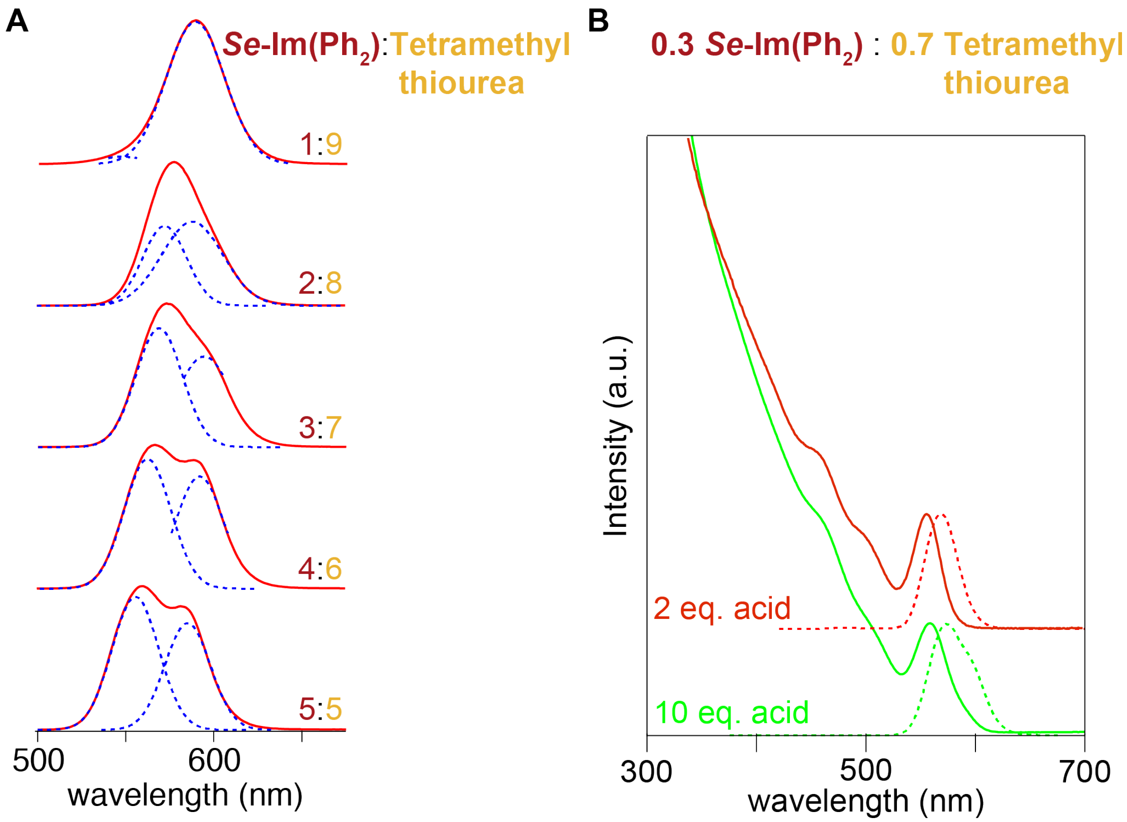


**Figure S20**. Reactions can be optimized to remove a second population of emitters. (a) Sulfur to selenium precursor ratio dependence on the appearance of a second population of emitters in the nanocrystal fluorescence spectra and (b) acid dependence on the appearance of the second population of emitters.

## Raman Spectroscopy

### *Experimental Approach*

We measured Raman spectra of the alloy series (CdSe, $CdSe_{0.75}S_{0.25}$, $CdSe_{0.50}S_{0.50}$, $CdSe_{0.25}S_{0.75}$, and CdS) and core shell samples (CdSe/CdS and CdS/CdSe) described within this paper as well as alloys synthesized via the method of Hens and coworkers ($CdSe_{0.75}S_{0.25}$, $CdSe_{0.50}S_{0.50}$, $CdSe_{0.25}S_{0.75}$).[3]

### *Raman Spectra of CdSe/S Alloys*

The spectra for the ~2.6 nm diameter alloy series are shown in Figure 6B and with their fits in Figure S21. We fit the main CdSe stretch peak at ~ 200 $cm^{-1}$ and the main CdS stretch peak at ~300 $cm^{-1}$ to two Lorentzian functions each. The lower energy peak and higher energy peaks are assigned to surface optical (SO) and longitudinal optical (LO) phonons, respectively. We do not fit the overtones visible in many spectra. The pure CdS nanocrystal requires a third, unassigned peak for a good fit. Fit results are in Tables S2-S6. We focus here on the LO phonon frequencies. The pure CdSe and CdS LO phonon peaks occur at 206 $cm^{-1}$ and 302 $cm^{-1}$, respectively. These frequencies are slightly smaller than the bulk values of 211 $cm^{-1}$ and 305 $cm^{-1}$ which is the expected trend for nanocrystals with confined phonons, although temperature-dependent anharmonic phonon-phonon coupling may slightly counteract this shift.[4-7] As shown in Figure 6C, the CdSe LO frequency increases and the CdS LO frequency decreases as the selenium content of the alloys increases. This peak frequency evolution is consistent with two-mode behavior observed in both bulk and nanocrystalline alloyed CdSe/S samples in which both the CdSe and CdS LO phonons have distinct peaks.[8-9] Our peak frequencies are in good agreement with a recent study by Mukherjee et al.[8] on alloyed CdSe/S nanocrystals of very similar size. This report also observed that the frequency difference between the CdS and CdSe LO phonon peaks decreases with greater selenium content, and can predict the composition of the nanocrystal. Our data is consistent with this relationship.

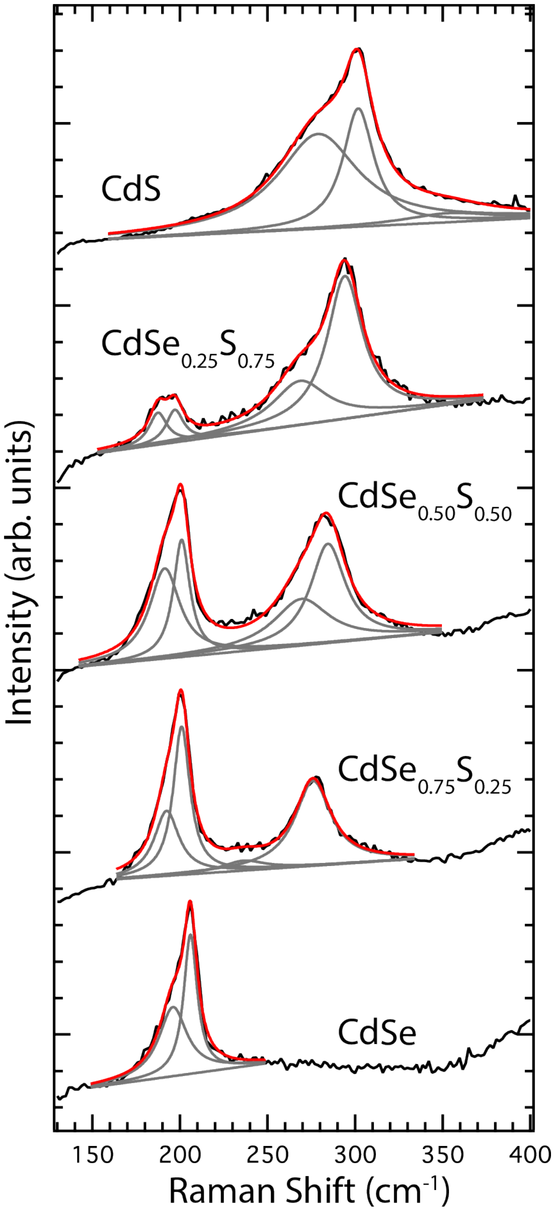


**Figure S21**. Raman spectra with corresponding fits of alloyed nanoparticles CdSe, $CdSe_{0.75}S_{0.25}$, $CdSe_{0.50}S_{0.50}$, $CdSe_{0.25}S_{0.75}$, and CdS synthesized as described in this work. The black, red, and gray curves are the data, overall fit, and individual peak fits, respectively.

***Raman Spectra of CdSeS Alloys Synthesized via Hens and Coworkers Method***

In contrast to the "two-mode" behavior observed in our alloyed nanocrystals, another study observed a single LO phonon peak for CdSe/S alloys, where the LO peak

frequency varies smoothly from the pure CdSe LO frequency to the pure CdS LO frequency as a function of composition.[3] Raman spectra of samples that we synthesized using the procedure from this report did not reproduce this trend. The measured spectra contain distinct LO peaks for both CdSe and CdS over a range of compositions (Figure S22), rather than a single LO peak as observed previously.

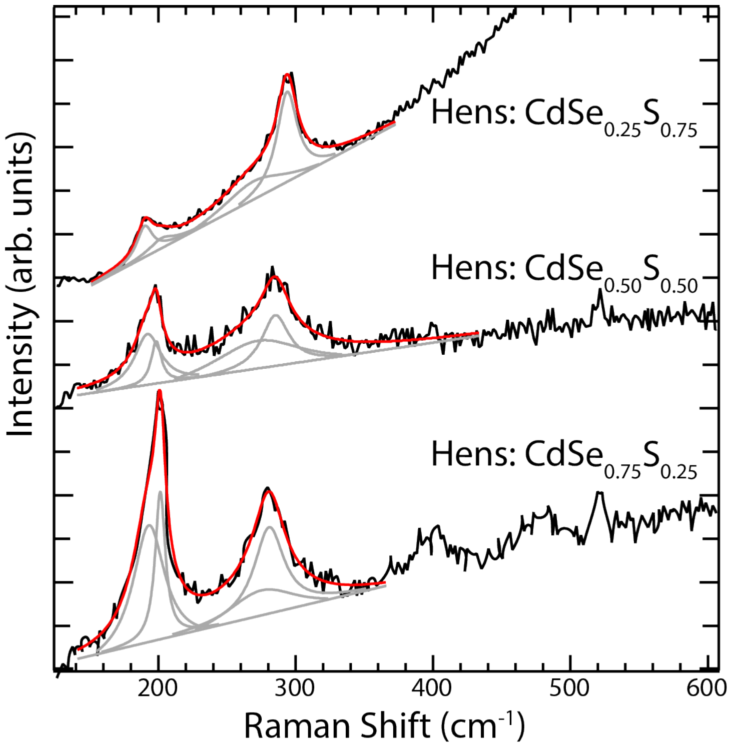


**Figure S22**. Raman spectra with corresponding fits of alloyed nanoparticles $CdSe_{0.75}S_{0.25}$, $CdSe_{0.50}S_{0.50}$, $CdSe_{0.25}S_{0.75}$ synthesized via the method of Hens and coworkers.[3] The black, red, and gray curves are the data, overall fit, and individual peak fits, respectively.

***A Comparison Between Alloyed and Core/Shell Nanocrystals***

Raman spectra for core/shell nanocrystals composed of CdSe and CdS also have distinct peaks for the CdSe and CdS LO phonons.[10] We compare Raman results from alloys to deliberately synthesized core/shell nanocrystals with sharp interfaces (Figure S23). Overall, comparisons of the alloys to core shell materials in the literature are difficult because of differences in diameter, temperature, shell thickness, and sulfur or selenium content. The 2.8 nm diameter CdS/CdSe core/shell nanocrystal synthesized here is ~50% S and ~50% Se and has CdSe and CdS LO phonon frequencies of 194.61 cm$^{-1}$ and 295.5 cm$^{-1}$, respectively. In contrast, for the $CdSe_{0.50}S_{0.50}$ alloy these peaks occur at 201 cm$^{-1}$

and 284.5 cm$^{-1}$, frequencies slightly different than core/shell nanocrystals with a comparable size and Se:S ratio. A comparable analysis of 3.5 nm diameter CdSe/CdS core/shell nanocrystals is less definitive because the two nanocrystals have different diameters. These core/shell samples are ~90% S, so we compare peak frequencies to $CdSe_{0.25}S_{0.75}$. For CdSe/CdS, the LO frequencies are 198.4 cm$^{-1}$ and 294.2 cm$^{-1}$, which is not very different from the $CdSe_{0.25}S_{0.75}$ frequencies (197.1 cm$^{-1}$ and 294.1 cm$^{-1}$). Frequency based arguments do not provide enough to be a measure of the degree of phase mixing in the alloys.

.

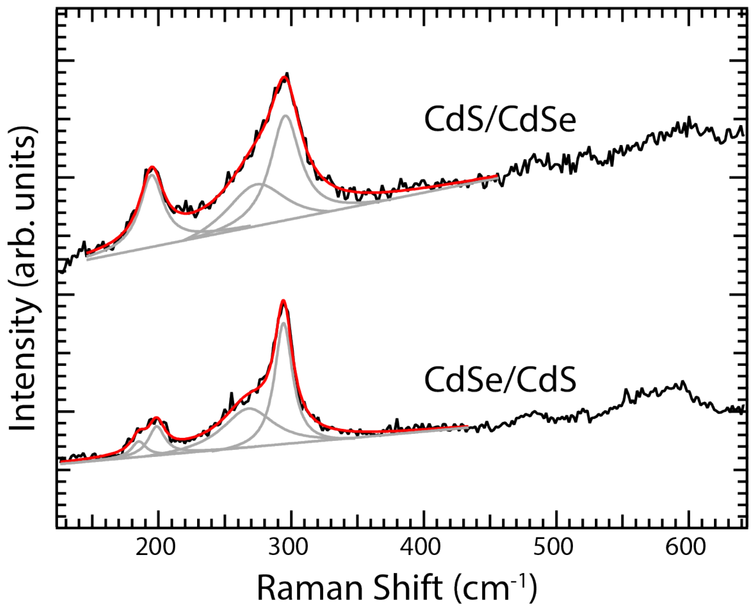


**Figure S23**. Raman spectra with corresponding fits of CdSe/CdS and CdS/CdSe core/shell nanoparticles synthesized as described in this work. The black, red, and gray curves are the data, overall fit, and individual peak fits, respectively.

***Fit Parameter Tables***

**Table S2**: CdSe Surface Optical Phonon Fit Parameters

| Sample | CdSe Surface Optical Phonon | | | |
|---|---|---|---|---|
| | ω (cm$^{-1}$) | FWHM (cm$^{-1}$) | Height | Area (cm$^{-1}$) |
| CdS | | | | |
| $CdSe_{0.25}S_{0.75}$ | 187.4 ± 1.3 | 12.5 ± 3.0 | 251 ± 50 | 4900 ± 1900 |

| | | | | |
|---|---|---|---|---|
| $CdSe_{0.50}S_{0.50}$ | 191.3 ± 1.2 | 21.1 ± 1.6 | 458 ± 55 | 15200 ± 2600 |
| $CdSe_{0.75}S_{0.25}$ | 192.4 ± 1.1 | 17.1 ± 1.6 | 356 ± 54 | 9600 ± 2100 |
| CdSe | 196.0 ± 1.0 | 19.4 ± 1.5 | 118 ± 10 | 3600 ± 500 |
| CdS/CdSe | | | | |
| CdSe/CdS | 184.9 ± 1.6 | 13.6 ± 4.7 | 27.1 ± 5.8 | 580 ± 260 |
| Hens $CdSe_{0.25}S_{0.75}$ | 193.1 ± 1.3 | 28.3 ± 1.9 | 298 ± 33 | 13200 ± 1900 |
| Hens $CdSe_{0.50}S_{0.50}$ | 192.2 ± 3.4 | 23.8 ± 3.3 | 45 ± 20 | 1680 ± 760 |
| Hens $CdSe_{0.75}S_{0.25}$ | 190.0 ± 1.2 | 14.0 ± 5.2 | 72 ± 25 | 1600 ± 1100 |

**Table S3**: CdSe Longitudinal Optical Phonon Fit Parameters

| | **CdSe Longitudinal Optical Phonon** | | | |
|---|---|---|---|---|
| Sample | ω ($cm^{-1}$) | FWHM ($cm^{-1}$) | Height | Area ($cm^{-1}$) |
| CdS | | | | |
| $CdSe_{0.25}S_{0.75}$ | 197.1 ± 1.2 | 12.0 ± 3.0 | 255 ± 51 | 4800 ±1900 |
| $CdSe_{0.50}S_{0.50}$ | 201.0 ± 0.3 | 11.5 ± 1.2 | 595 ± 67 | 10800 ± 2200 |
| $CdSe_{0.75}S_{0.25}$ | 200.9 ± 0.3 | 11.6 ± 0.8 | 813 ± 62 | 14800 ± 2000 |
| CdSe | 206.0 ± 0.2 | 9.4 ± 0.7 | 239 ± 13 | 3500 ± 400 |
| CdS/CdSe | 194.6 ± 0.3 | 21.5 ± 1.0 | 122.9 ± 3.8 | 4200 ± 200 |
| CdSe/CdS | 198.4 ± 1.0 | 15.1 ± 2.8 | 50.2 ± 5.3 | 1200 ± 300 |
| Hens $CdSe_{0.25}S_{0.75}$ | 201.4 ± 0.2 | 9.0 ± 1.3 | 341 ± 37 | 4800 ± 1100 |
| Hens $CdSe_{0.50}S_{0.50}$ | 198.5 ± 0.9 | 9.0 ± 5.4 | 36 ± 21 | 510 ± 590 |
| Hens $CdSe_{0.75}S_{0.25}$ | 202.3 ± 9.2 | 21.6 ± 11.2 | 23 ± 30 | 800 ± 1300 |

**Table S4**: CdS Surface Optical Phonon Fit Parameters

| | **CdS Surface Optical Phonon** | | | |
|---|---|---|---|---|
| Sample | ω ($cm^{-1}$) | FWHM ($cm^{-1}$) | Height | Area ($cm^{-1}$) |
| CdS | 278.9 ± 1.0 | 55.8 ± 1.4 | 2598 ±77 | 228000 ± 11000 |
| $CdSe_{0.25}S_{0.75}$ | 268.5 ± 1.7 | 37.7 ± 3.9 | 345 ± 24 | 20500 ± 2900 |
| $CdSe_{0.50}S_{0.50}$ | 269.3 ± 4.9 | 38.3 ± 4.9 | 237 ± 88 | 14200 ± 6700 |

| | | | | |
|---|---|---|---|---|
| $CdSe_{0.75}S_{0.25}$ | 236.4 ± 2.6 | 29.41 ± 10.7 | 54.4 ± 8.5 | 2514.5 ± 830 |
| CdSe | | | | |
| CdS/CdSe | 273.6 ± 3.3 | 58.9 ± 3.6 | 91 ± 13 | 8400 ± 1500 |
| CdSe/CdS | 267.6 ± 1.6 | 48.4 ± 4.2 | 72.2 ± 3.0 | 5480 ± 580 |
| Hens $CdSe_{0.25}S_{0.75}$ | 275.1 ± 7.5 | 68.4 ± 39.6 | 82 ± 89 | 8800 ± 5200 |
| Hens $CdSe_{0.50}S_{0.50}$ | 272.6 ± 6.5 | 84.7 ± 18.1 | 33 ± 11 | 4340 ± 690 |
| Hens $CdSe_{0.75}S_{0.25}$ | 269.3 ± 3.5 | 58.6 ± 6.9 | 55.2 ± 5.5 | 5080 ± 870 |

**Table S5**: CdS Longitudinal Optical Phonon Fit Parameters

| | **CdS Longitudinal Optical Phonon** | | | |
|---|---|---|---|---|
| Sample | ω ($cm^{-1}$) | FWHM ($cm^{-1}$) | Height | Area ($cm^{-1}$) |
| CdS | 301.9 ± 0.2 | 21.4 ± 0.9 | 3240 ± 110 | 109000 ± 7400 |
| $CdSe_{0.25}S_{0.75}$ | 294.1 ± 0.3 | 24.4 ± 1.0 | 1119 ± 31 | 42900 ± 2500 |
| $CdSe_{0.50}S_{0.50}$ | 284.5 ± 1.0 | 24.2 ± 3.1 | 504 ± 99 | 19200 ± 6100 |
| $CdSe_{0.75}S_{0.25}$ | 276.0 ± 0.3 | 24.9 ± 0.9 | 466.2 ± 9.2 | 18200 ± 600 |
| CdSe | | | | |
| CdS/CdSe | 295.5 ± 0.5 | 27.6 ± 2.3 | 185 ± 15 | 8000 ± 1300 |
| CdSe/CdS | 294.2 ± 0.2 | 16.5 ± 0.7 | 213.3 ± 5.3 | 5510 ± 320 |
| Hens $CdSe_{0.25}S_{0.75}$ | 280.6 ± 0.9 | 26.6 ± 7.5 | 201 ± 88 | 8400 ± 5900 |
| Hens $CdSe_{0.50}S_{0.50}$ | 285.1 ± 1.0 | 24.4 ± 6.5 | 45 ± 11 | 1730 ± 830 |
| Hens $CdSe_{0.75}S_{0.25}$ | 293.6 ± 0.2 | 18.2 ± 1.2 | 214.3 ± 8.0 | 6110 ± 550 |

**Table S6**: Third, higher frequency peak in CdS Raman spectrum

| | **CdS 3rd Peak** | | | |
|---|---|---|---|---|
| Sample | ω ($cm^{-1}$) | FWHM ($cm^{-1}$) | Height | Area ($cm^{-1}$) |
| CdS | 353.6 ± 3.5 | 59.8 ± 11.1 | 249 ± 27 | 23400 ± 4600 |

## Kinetics modeling

The modeling approach used here is based on a kinetic scheme where selenium and sulfur precursors co-react with cadmium precursor and form nuclei which grow larger through the co-deposition of CdSe and CdS onto the nuclei. According to the previous experimental observation, the growth of the nanocrystals follows the 1st reaction kinetics, where the reaction of the chalcogen precursors to form monomers is the rate-limiting step:

$$CdX_2 \quad + \quad \begin{matrix} Se - precursor & \overset{k_{Se}}{\longrightarrow} & CdSe\ monomer \\ S - precursor & \overset{k_{Se}}{\longrightarrow} & CdS\ monomer \end{matrix} \quad \rightarrow \quad CdS_xSe_{1-x}\ nanocrystal$$

The rate equations are written as:

$$\frac{d[CdSe]}{dt} = -\frac{d[Se]}{dt} = k_{Se} \cdot [Se] \qquad (1)$$

$$\frac{d[CdS]}{dt} = -\frac{d[S]}{dt} = k_S \cdot [S] \qquad (2)$$

where $[CdSe]$, $[CdS]$, $[Se]$ and $[S]$ are the concentrations of crystalized CdSe and CdS, selenium and sulfur precursors, respectively, and $k_{Se}$ and $k_S$ are the first order rate constant of selenium and sulfur precursors.

Based on the Sugimoto nucleation model, the number of nuclei in the end of the reaction ($n_\infty$) is proportional to the initial monomer generation rate, which is consistent with previous experiments. In our case, $n_\infty$ is given by:

$$n_\infty = A \cdot (k_{Se} \cdot [Se]_0 \ + \ k_S \cdot [S]_0) \qquad (3)$$

where $[Se]_0$ and $[S]_0$ are the initial precursor concentrations, and $A$ is a constant that can be experimentally determined.

Assuming the nucleation happens instantly in the beginning of the reaction and the number of nuclei stays constant throughout the reaction, we can calculate the size evolution of the particle based on:

$$\frac{dV_{QD}}{dt} = (\frac{d[CdSe]}{dt} \cdot V_{m,CdSe} + \frac{d[CdS]}{dt} \cdot V_{m,CdS}) \cdot V_{Reaction}/n_{\infty} \qquad (4)$$

where $V_{m,CdSe}$ and $V_{m,CdS}$ are the molar volume of CdSe and CdS, respectively, $V_{QD}$ is the volume of each QD, and $V_{Reaction}$ is the volume of the reaction solution. Solving this differential equation gives the time evolution of the volume of the QD, $V_{QD}(t)$. Assume the particle is spherical in shape, the radius evolution with time, $R_{QD}(t)$, can be calculated.

Meanwhile, the local composition (molar percentage of CdSe in CdE) at the surface of the particle at time t can be calculated by:

$$CdSe\%(t) = \frac{k_{Se} \cdot [CdSe](t)}{k_{Se} \cdot [CdSe](t) + k_{S} \cdot [CdS](t)} \cdot 100\% \qquad (5)$$

Now, the radial elemental profile within a particle can be presented by CdSe% versus $R_{QD}$.

**Simulated elemental profiles**

In a typical reaction with dissimilar chalcogen precursors (faster Se compound compared to S), the reaction of each precursor at the different stages of the synthesis gives rise to the radial compositional change in the nanocrystals, as shown in Figure S24. The faster Se compound react in the early stage in the synthesis and generates CdSe-rich cores. In the intermediate stage, the slower S compound starts to react while the faster Se compound has not been consumed yet, which results in the co-deposition of CdS and CdSe onto the particles to form a graded interfacial layer. In the final stage of the reaction, the faster Se compound has almost been depleted, leaving only the reaction of the remaining slower S compound, which produces a CdS-rich outer shell.

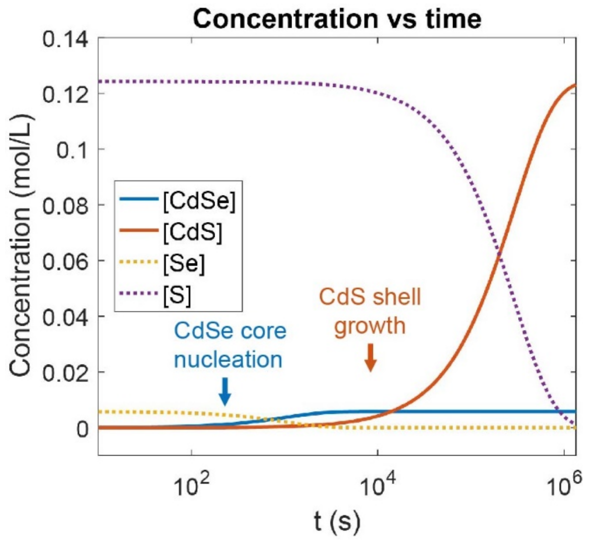


**Figure S24**. Calculated temporal evolution of the concentrations of crystallized CdSe and CdS, Se and S precursors in a typical heterostructure synthesis reaction.

The simulated elemental profile of a representative heterogeneous particle is shown in Figure S25. The composition change from the center to the surface of the particle follows a sigmoidal curve, in which the percentage of CdSe is highest at the center, and lowest on the surface. We divided the curve into three regions. The core region is defined as the sphere from the center outwards in which the compositional change is less than 5% of the total compositional change over the whole radius. Similarly, the outer shell region is defined as the shell from the surface inwards in which the compositional change is also less than 5% of the total compositional change. The inner layer between the core and the outer shell is defined as the graded alloyed interfacial region, inside which 90% of the total compositional change happens.

The six key features to describe a heterogeneous nanoparticle are the sizes and average compositions of the core, the interface and the shell region, which can all be calculated based the kinetic model.

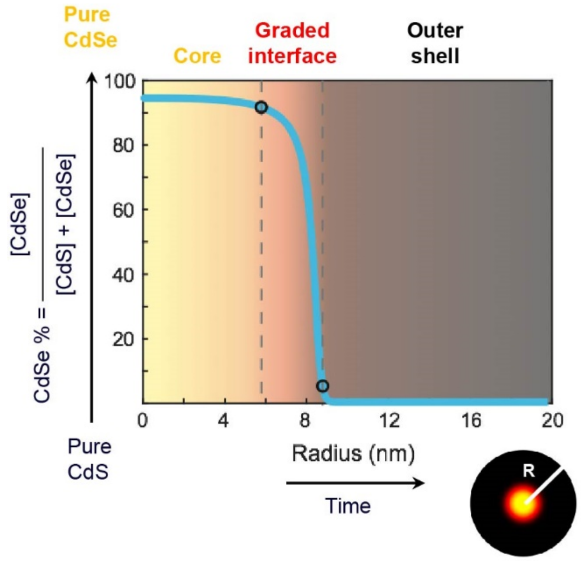


**Figure S25**. The simulated radial elemental distribution of a representative heterostructure nanoparticle. The circles and the dashed lines mark the arbitrary separation of the three regions.

## High-throughput screening

For a given set of four reaction parameters, including $[Se]_0$, $[S]_0$, $k_{Se}$ and $k_S$, the elemental profile of the particle synthesized can be calculated as described above. The question now is to what extent we can design the heterostructures by choosing the appropriate combination of reaction parameters. To answer this question, we performed high-throughput simulations over a large parameter space.

We first note that the quantities that defines the heterogeneity is the ratio between the initial concentrations and the ratio between the rate constants ($[Se]_0/[S]_0$ and $k_{Se}/k_S$). That means if the radial compositional change is normalized by the total radius of particle, we will obtain the same profile as long as the two ratios are the same. Meanwhile, the absolute values of these quantities determine the final particle size. Therefore, we choose to constrain the parameters to obtain particles of 5 nm in radius by:

$$V_{total\ CdE} = [Se]_0 \cdot V_{m,CdSe} + [S]_0 \cdot V_{m,CdS} = constant \qquad (6)$$

$$n_\infty = A \cdot (k_{Se} \cdot [Se]_0 + k_S \cdot [S]_0) = constant \qquad (7)$$

Under the two constraints, the number of independent variables ($[Se]_0/[S]_0$ and $k_{Se}/k_S$) is reduced to two.

We then independently vary these two ratios in a wide range ($10^{-4} < [Se]_0/[S]_0 < 10^4$ by $10^{-4} < k_{Se}/k_S < 10^4$) and perform the simulation to obtain the elemental profiles of the particles. Then, the structures of interest can be searched for from this database by imposing filters, such as core radius < 2 nm, shell CdSe% < 5%, etc.

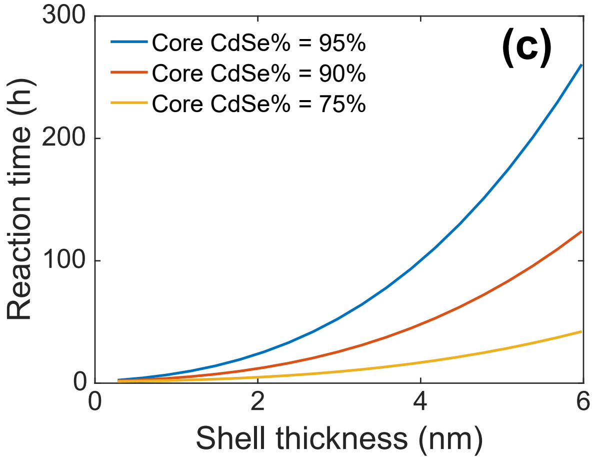


**Figure S26**. Reaction time needed for the growth of heterostructures as a function of shell thickness. Unpractically long reaction times are needed for thick-shell particles, especially when a high percentage of CdSe in the core is sought-after.

**Compound Characterization**

**Cadmium oleate.**

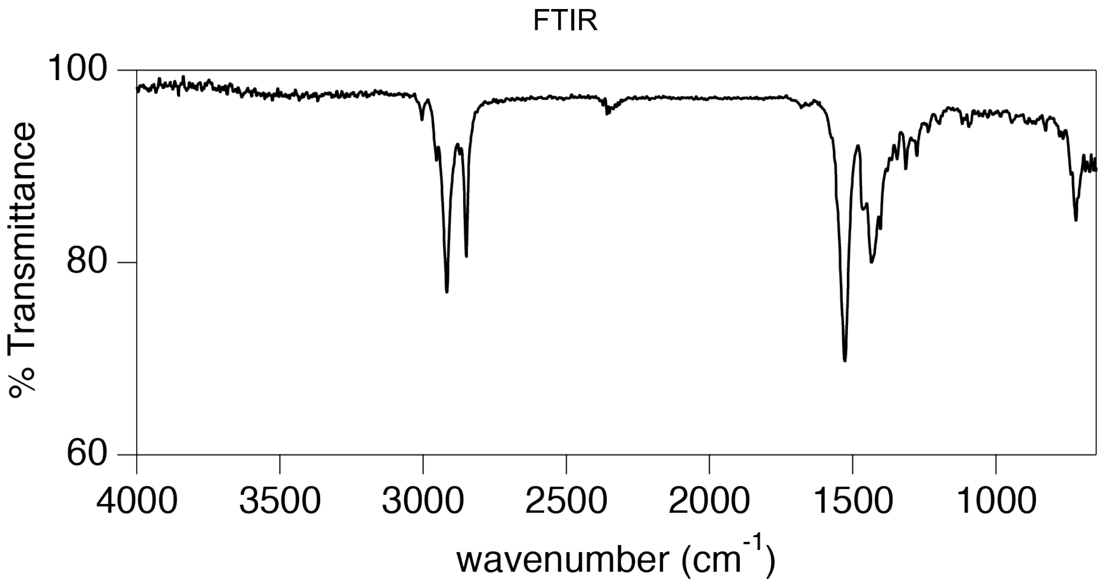


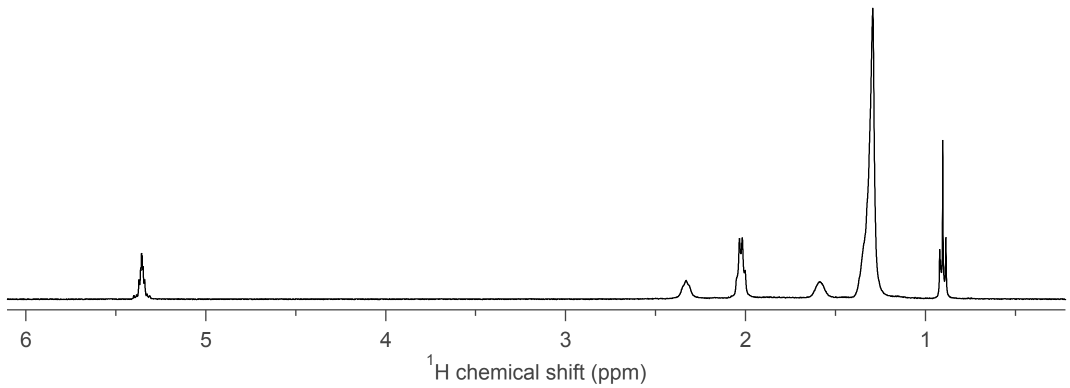


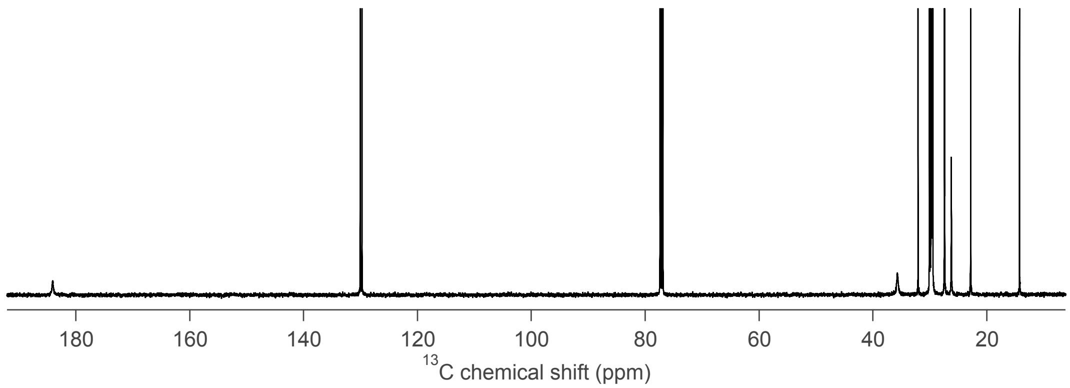

**1-phenylimidazolidine-2-thione (*S*-Im(H,Ph)).**

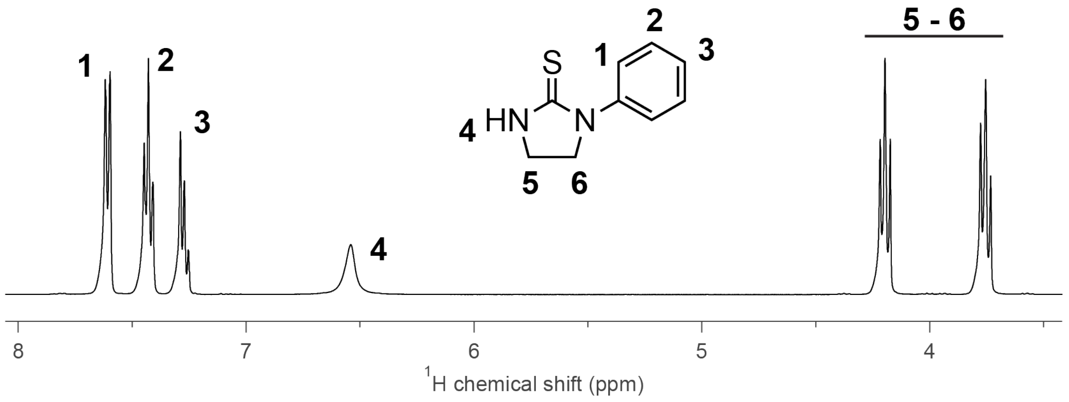


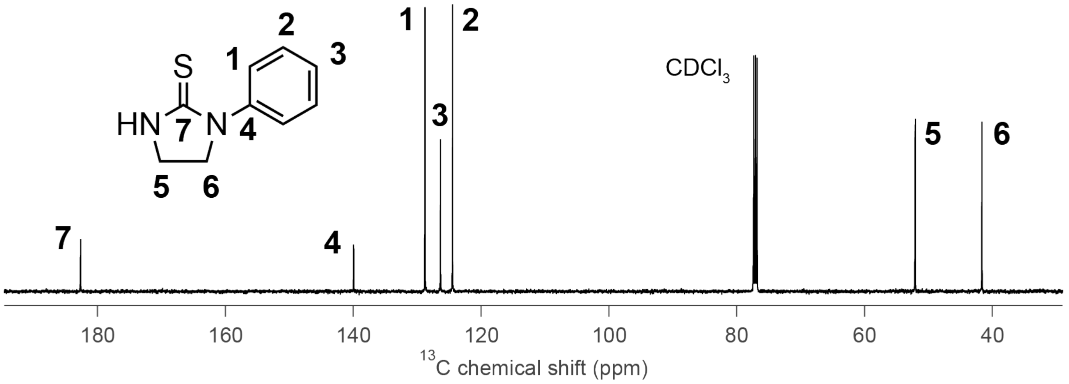


**1-methylimidazolidine-2-thione (*S*-Im(H,Me)).**

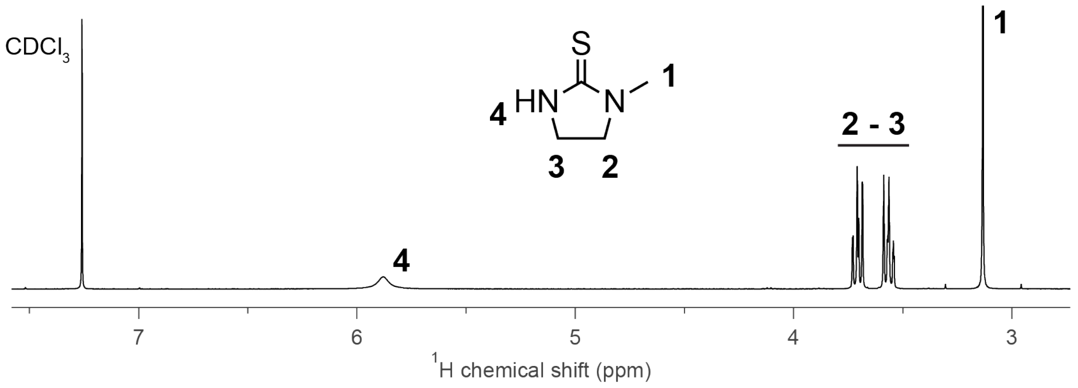

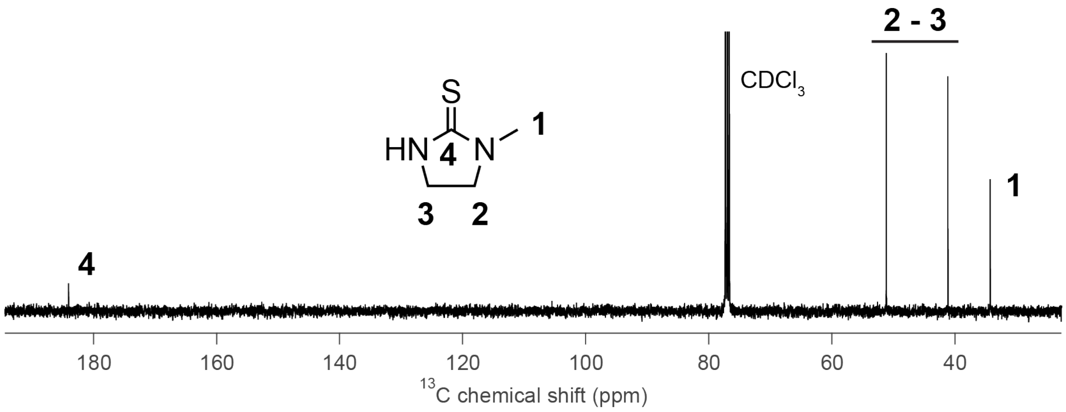


**1-ethylimidazolidine-2-thione (*S*-Im(H,Et)).**

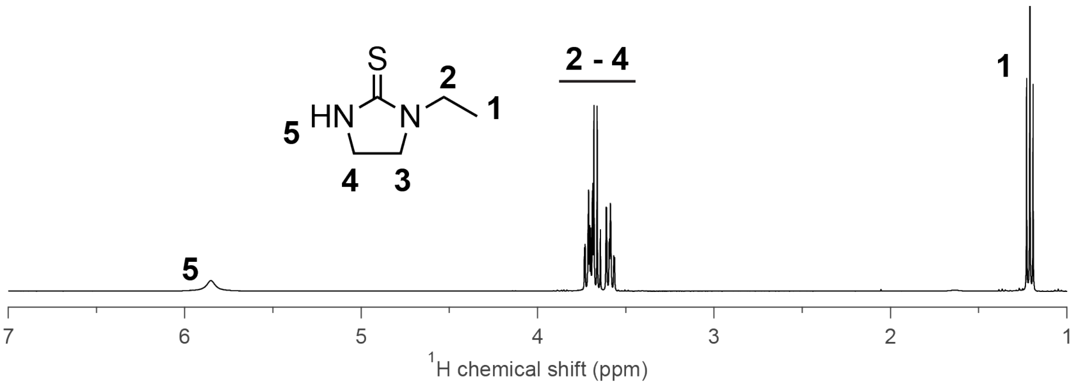


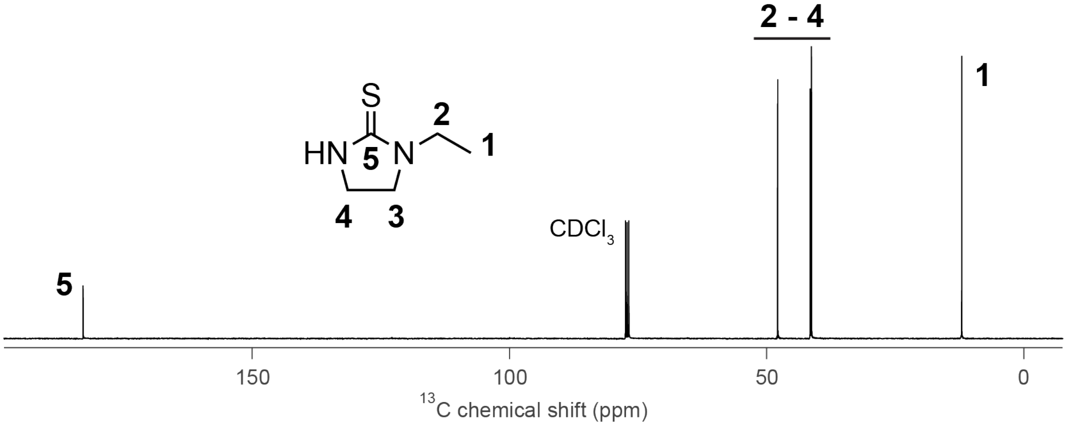

**1-isopropylimidazolidine-2-thione (*S*-Im(H,iPr)).**

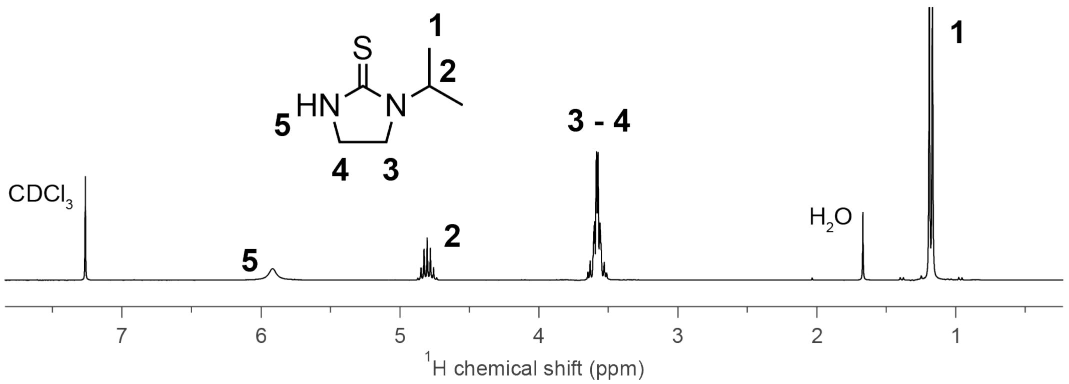


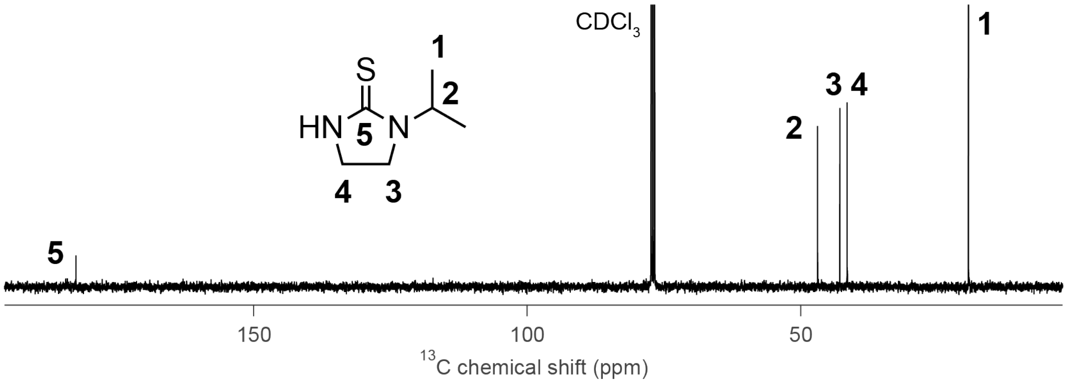


**1,3-diphenylimidazolidine-2-thione (*S*-Im(Ph₂)).**

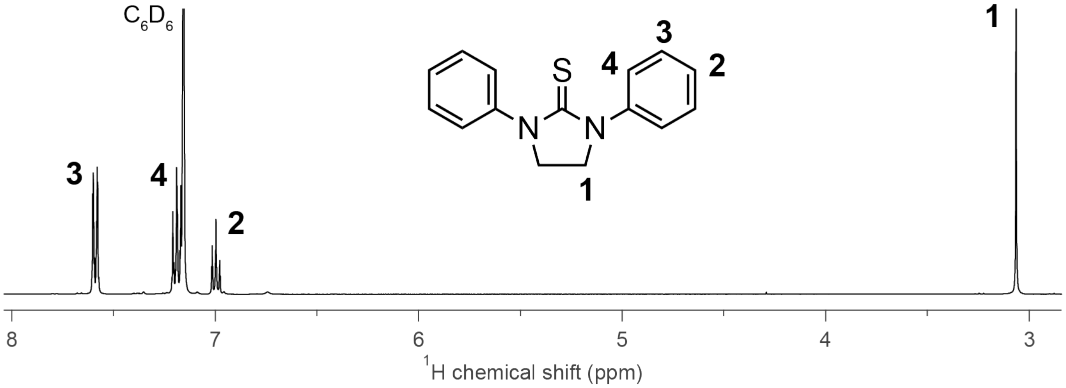

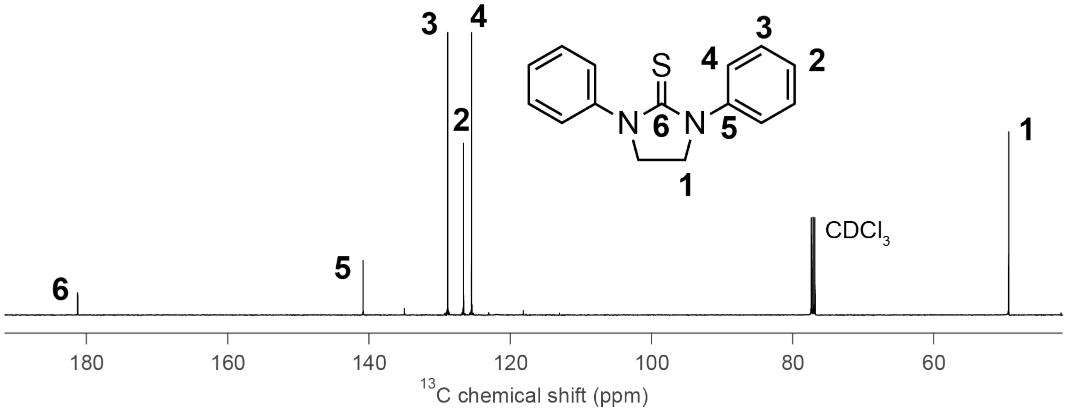


**1,3-dimethylimidazolidine-2-thione (*S*-Im(Me₂)).**

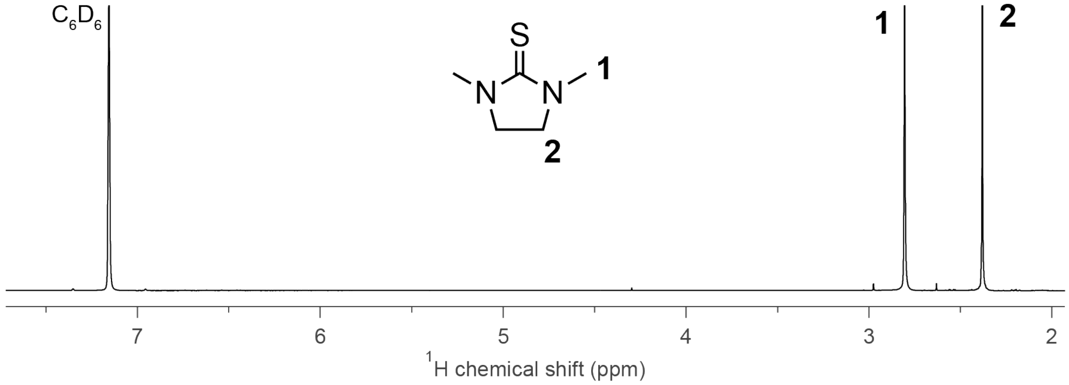


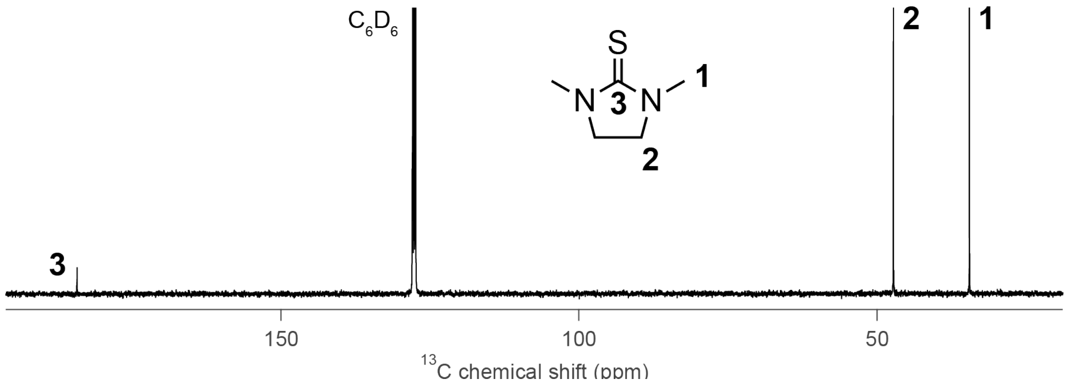

**1,3-diethylimidazolidine-2-thione (*S*-Im($Et_2$)).**

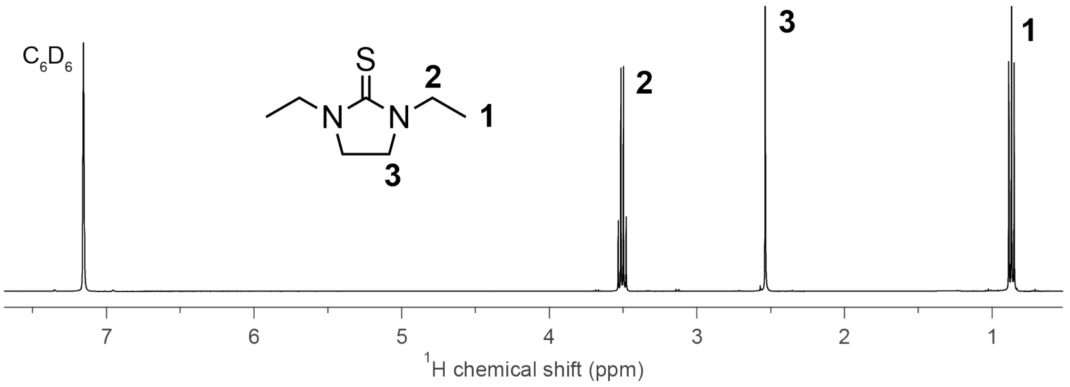


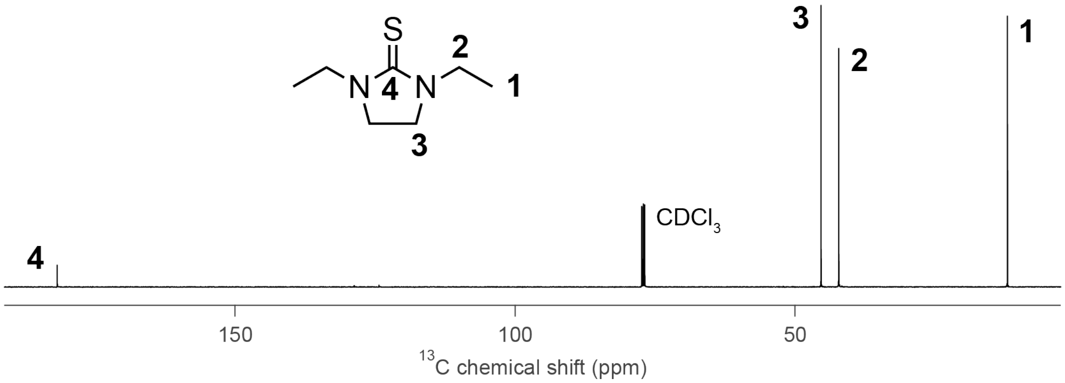


**1,3-diisopropylimidazolidine-2-thione (*S*-Im($iPr_2$)).**

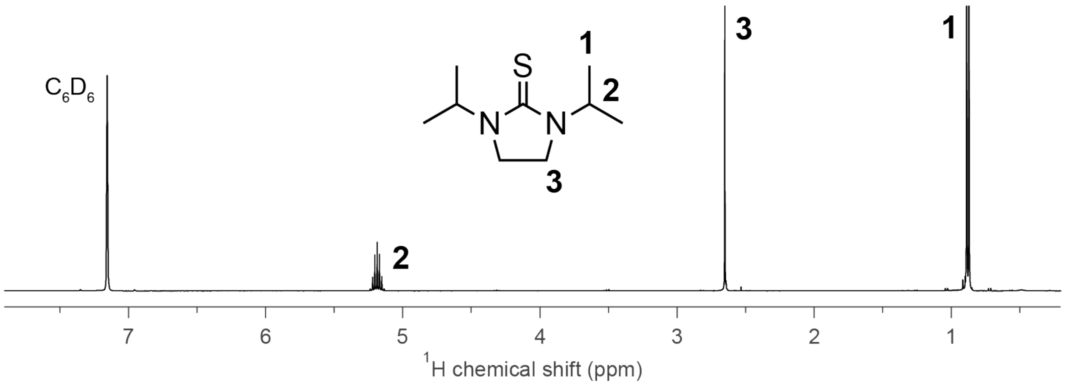

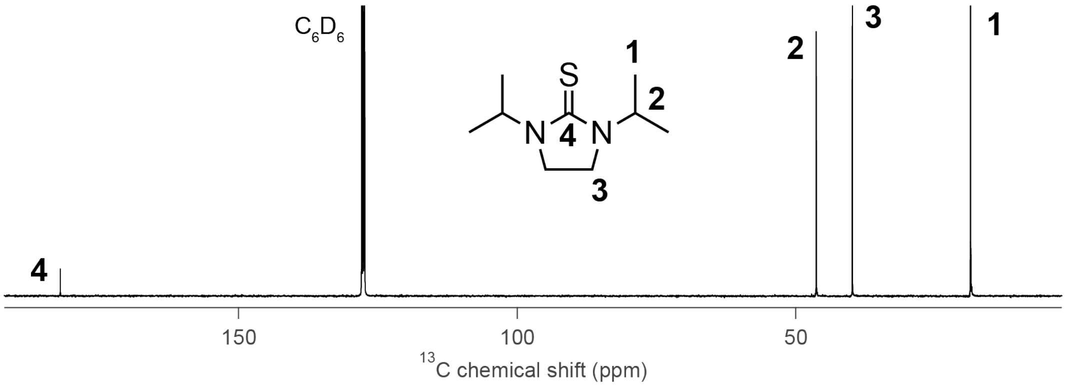


**1-methyltetrahydropyrimidine-2(1*H*)-thione (*S*-Pym(H,Me)).**

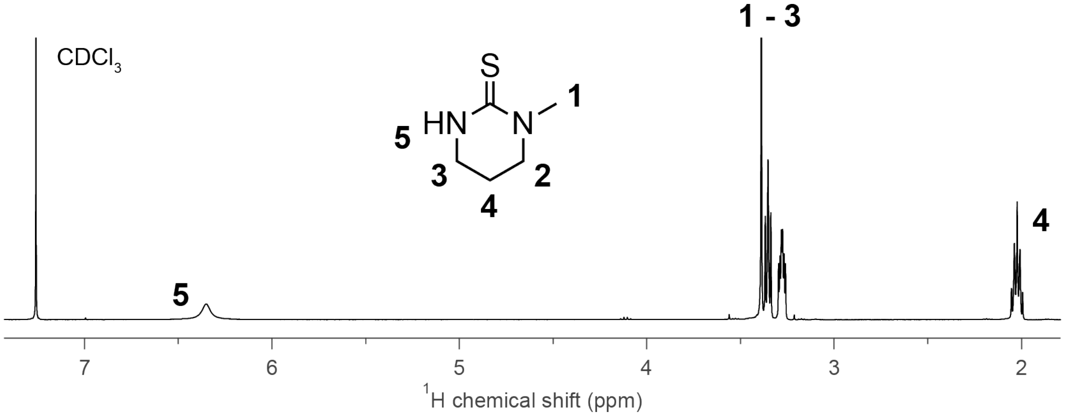


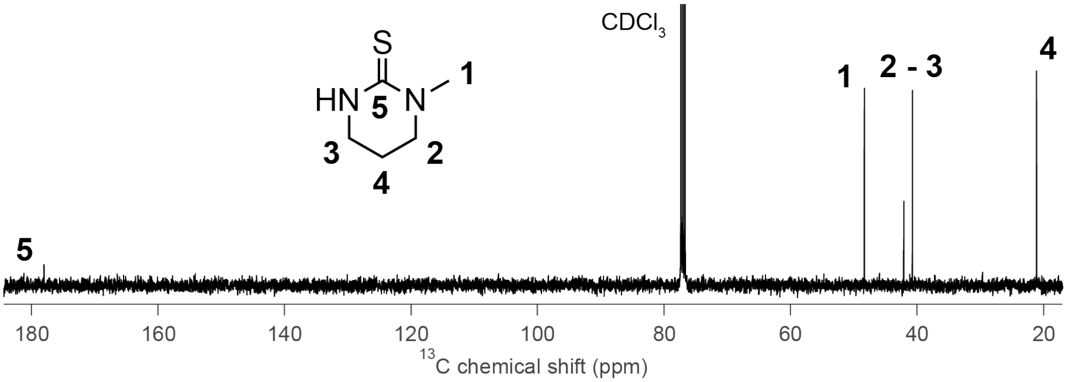

**1-isopropyltetrahydropyrimidine-2(1*H*)-thione (*S*-Pym(H,iPr)).**

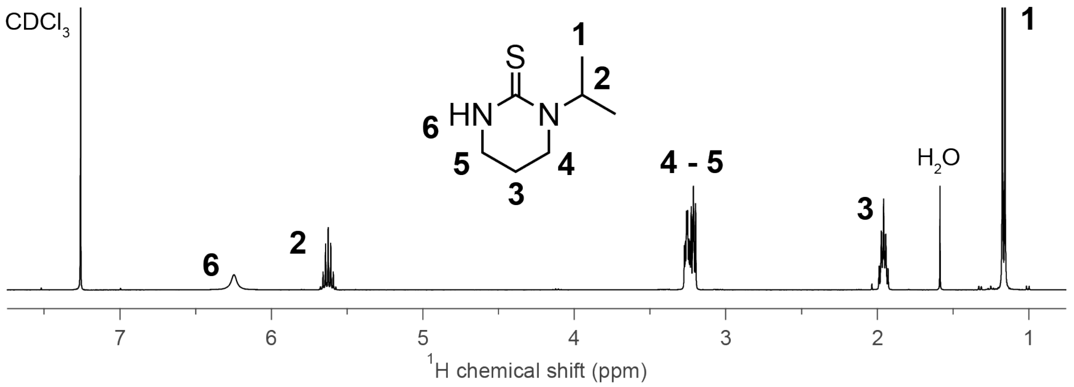


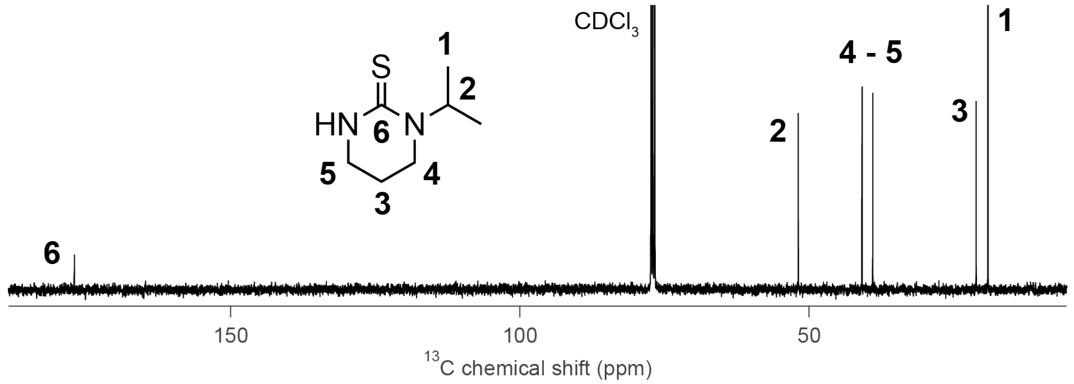


**1,3-dimethyltetrahydropyrimidine-2(1*H*)-thione (*S*-Pym(Me₂)).**

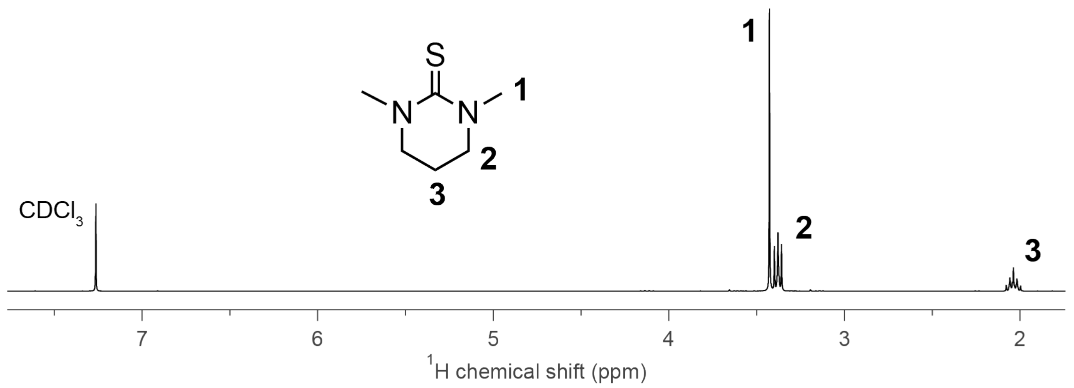

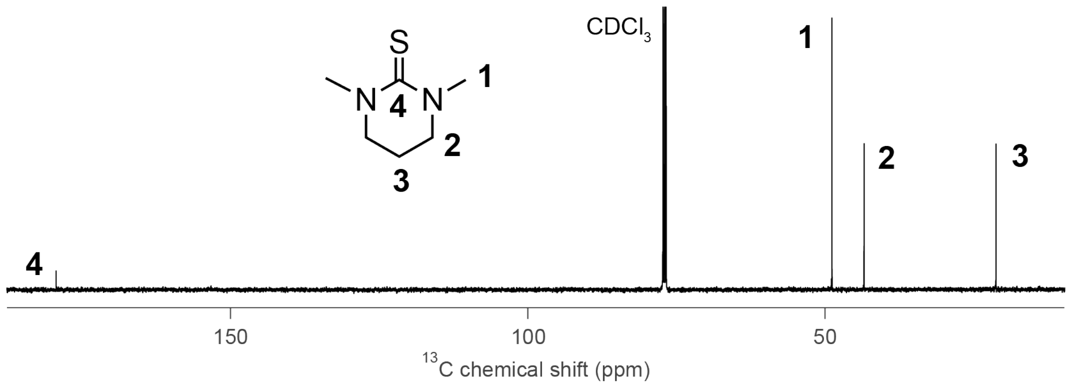


**1,3-diethyltetrahydropyrimidine-2(1*H*)-thione (*S*-Pym(Et$_2$)).**

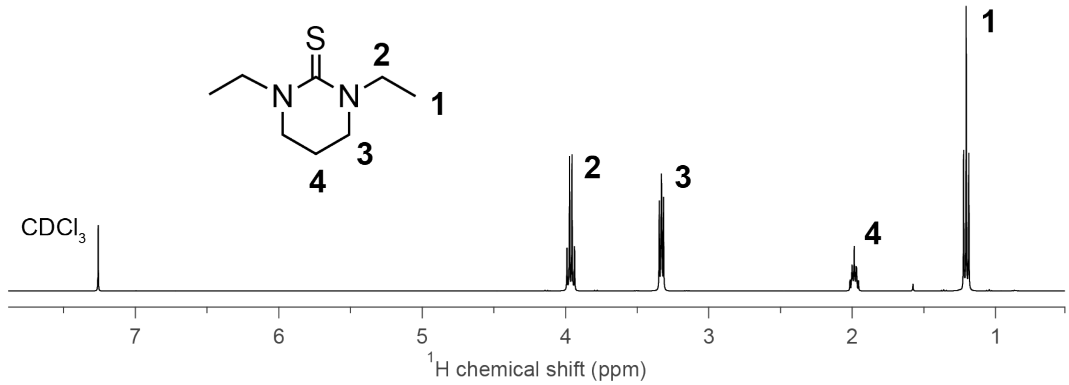


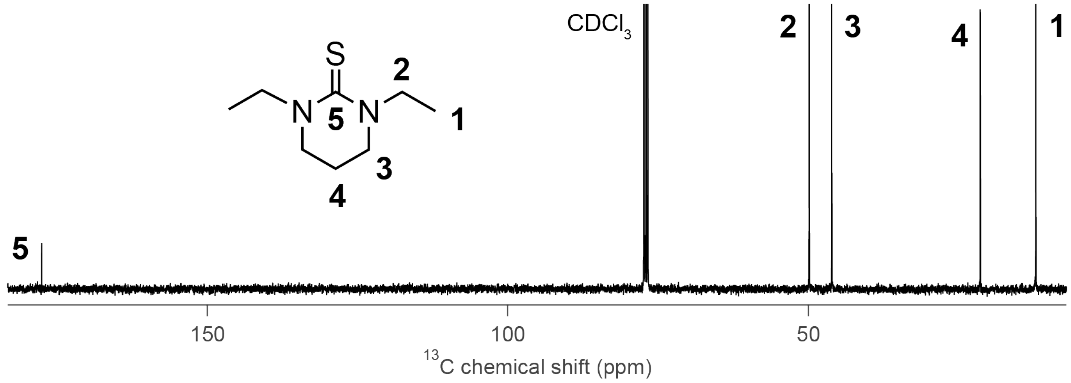

1,3-diisopropyltetrahydropyrimidine-2(1*H*)-thione (*S*-Pym(iPr₂)).

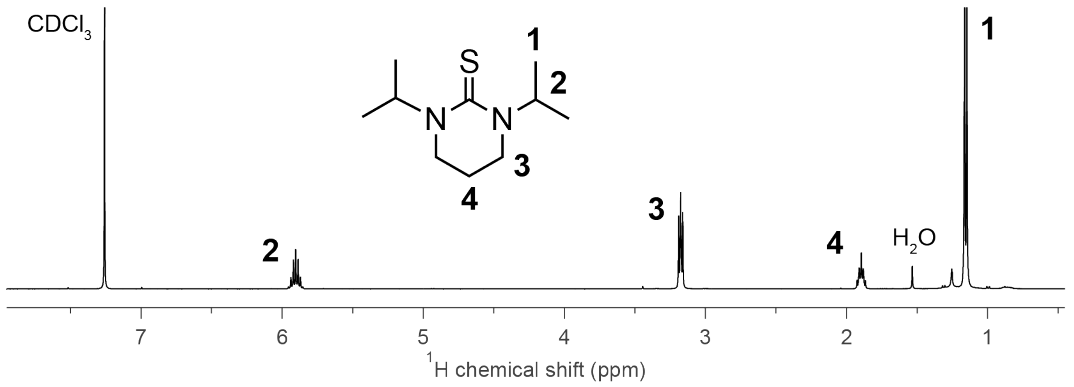


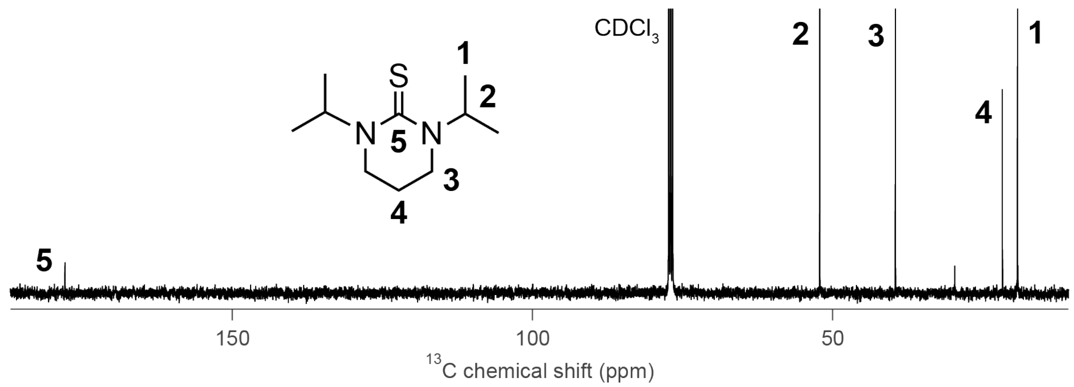


1-phenylimidazolidine-2-selenone (*Se*-Im(H,Ph)).

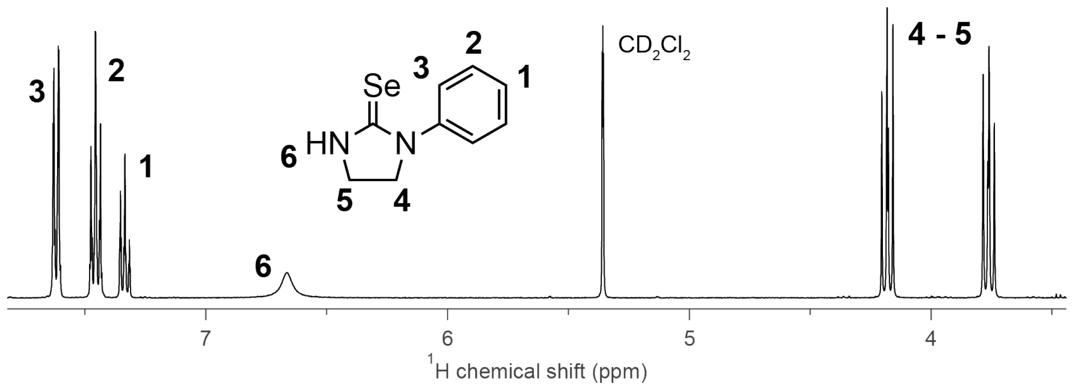

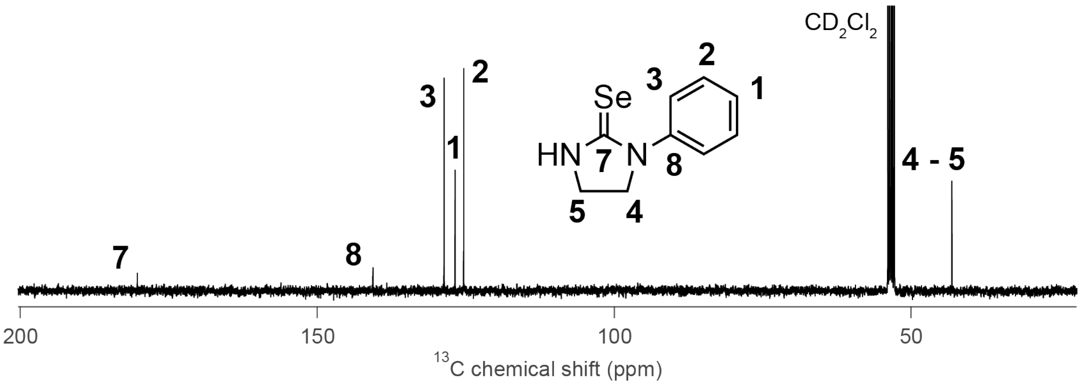


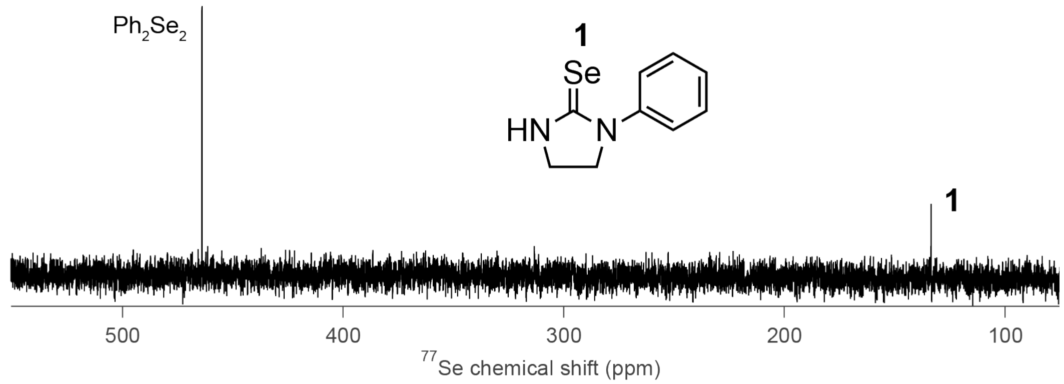


**1-ethylimidazolidine-2-selenone (*Se*-Im(H,Et)).**

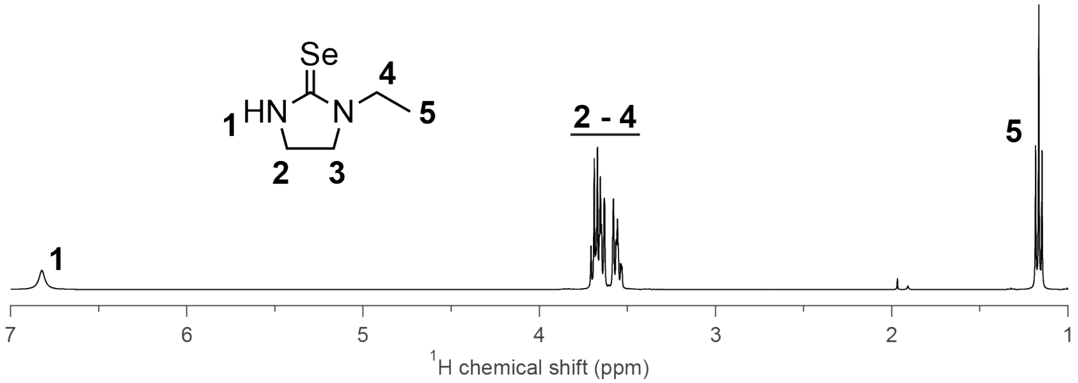

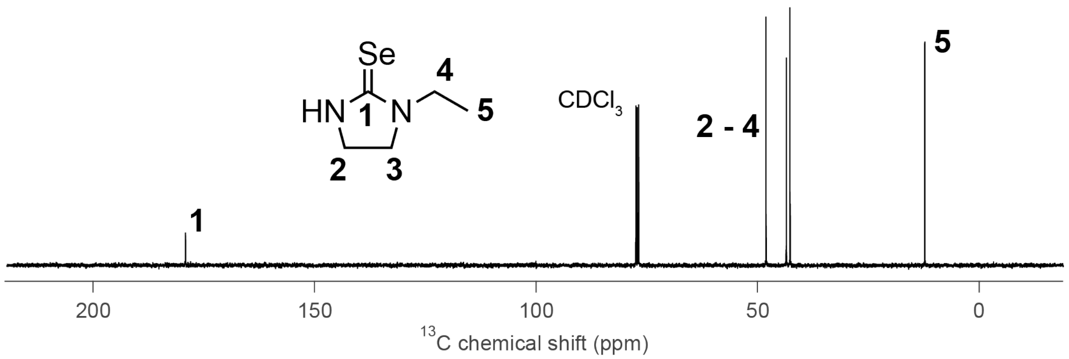


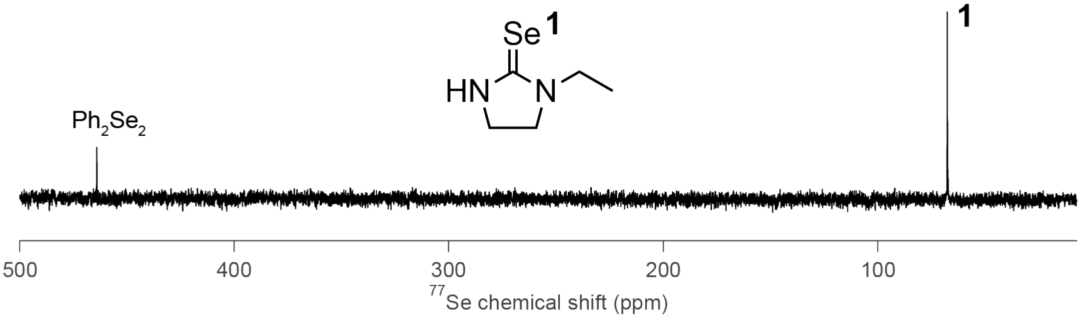


**1-isopropylimidazolidine-2-selenone (*Se*-Im(H,iPr)).**

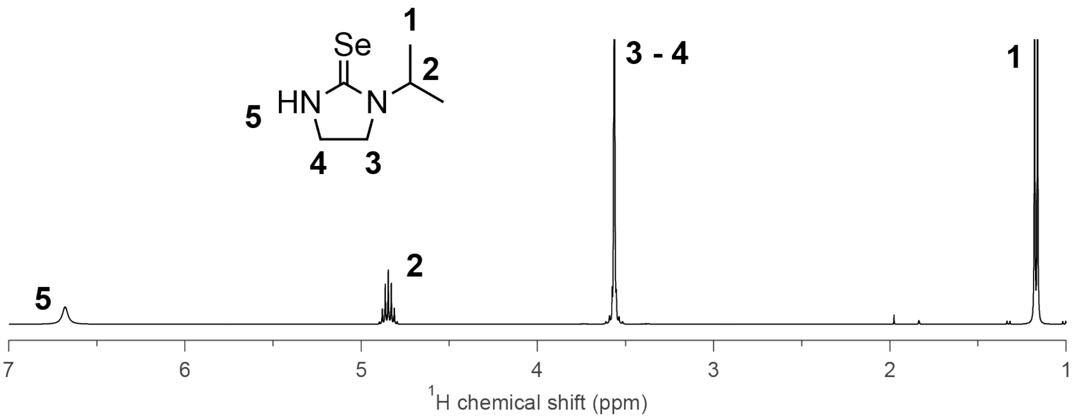

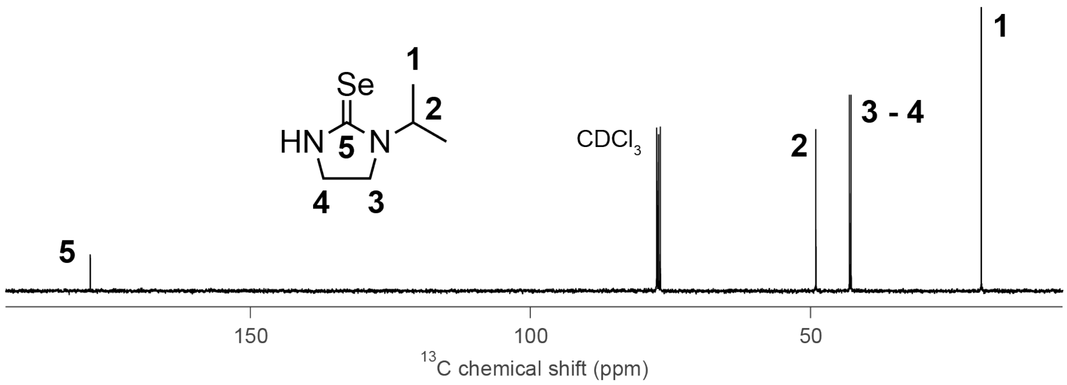


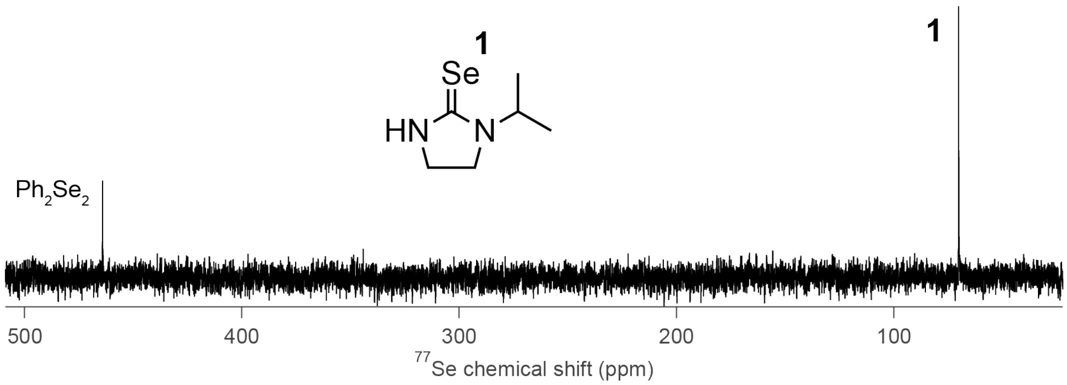


**1,3-diphenylimidazolidine-2-selenone (*Se*-Im(Ph$_2$))**

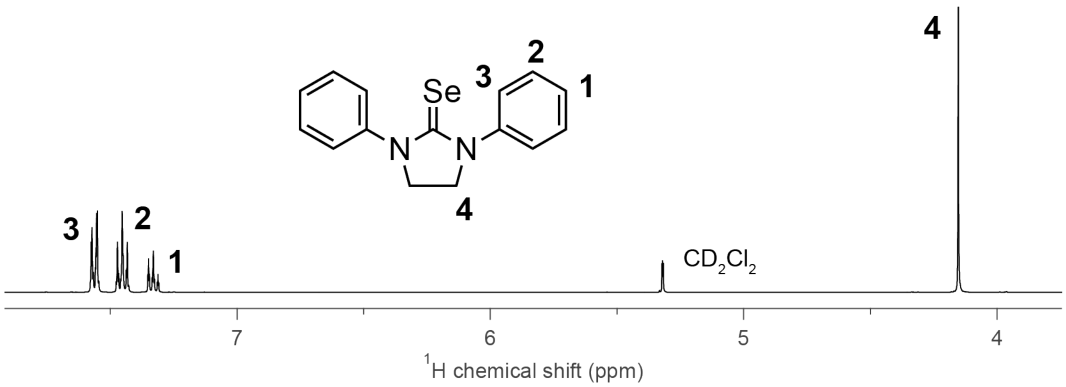

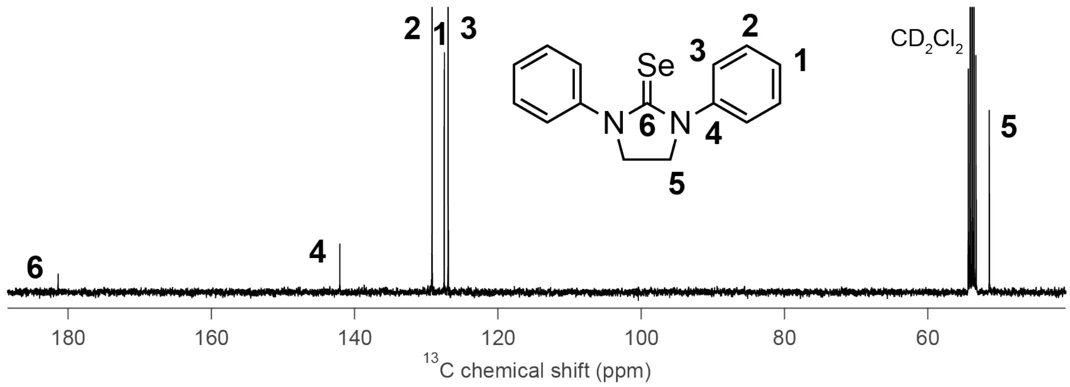


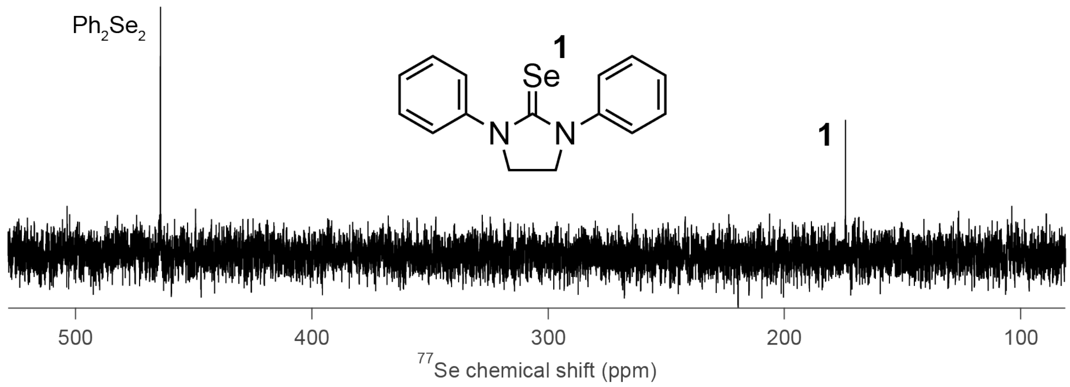


**1,3-ditertbutylimidazolidine-2-selenone (*Se*-Im(*t*-Bu$_2$))**

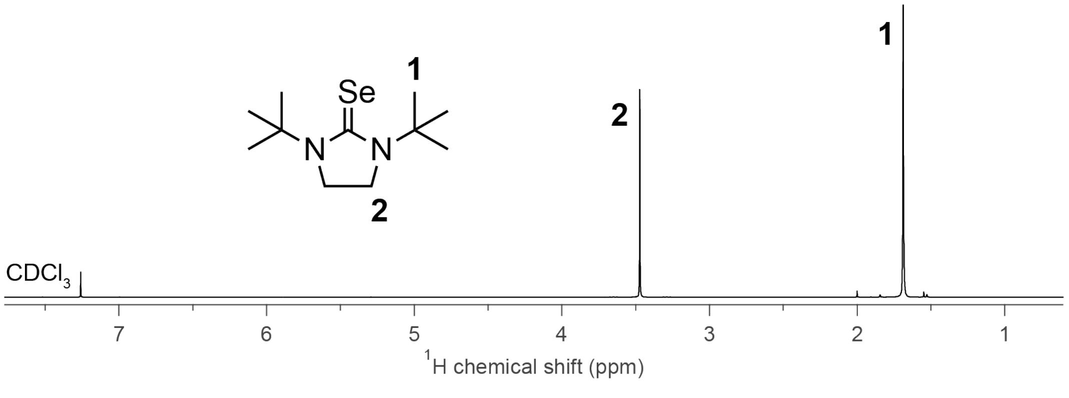

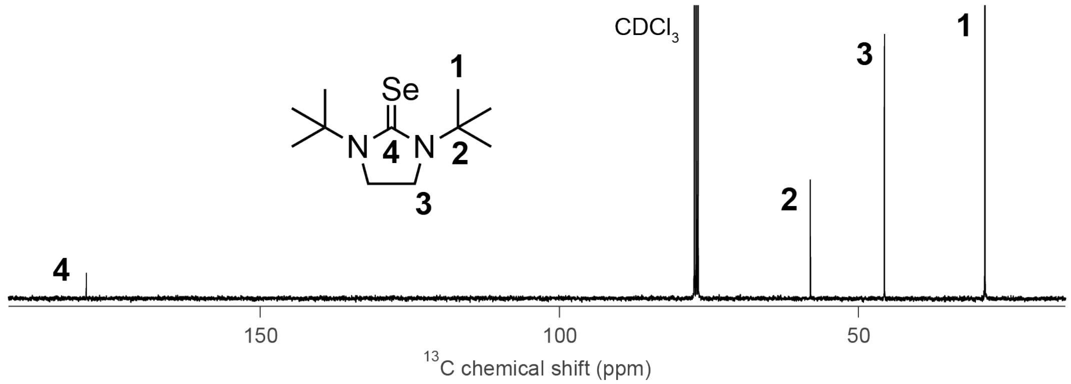


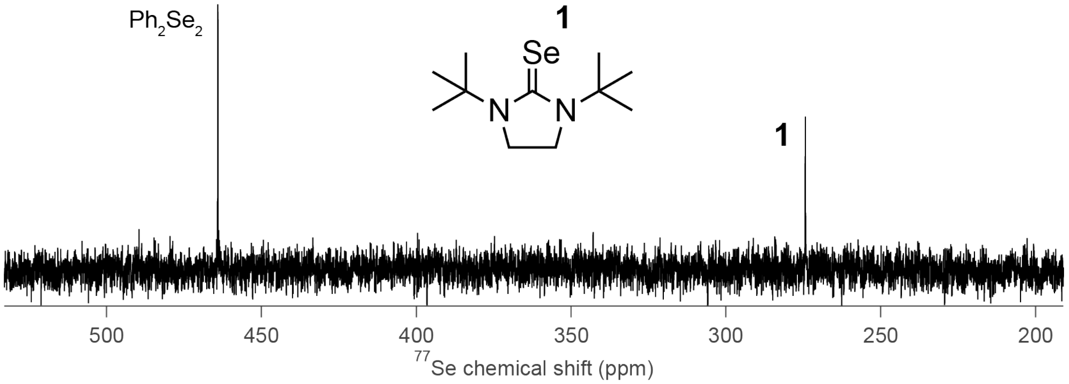


**1,3-dimethylimidazolidine-2-selenone (*Se*-Im(Me$_2$))**

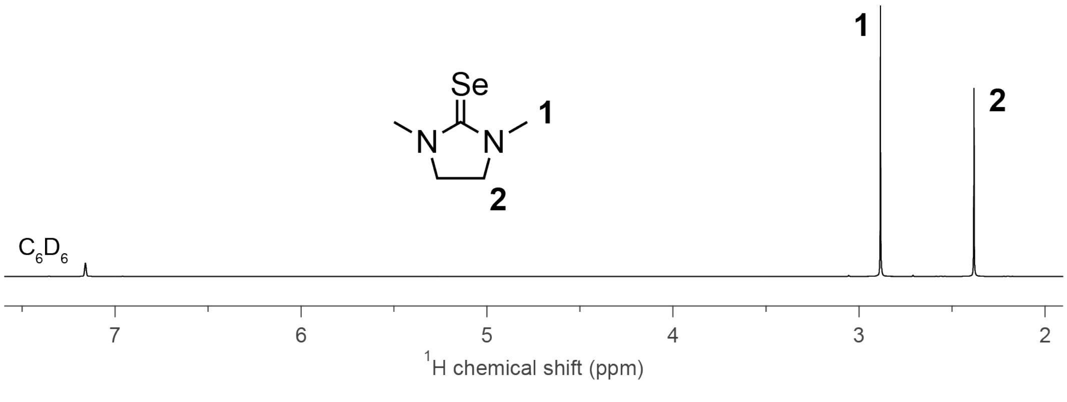

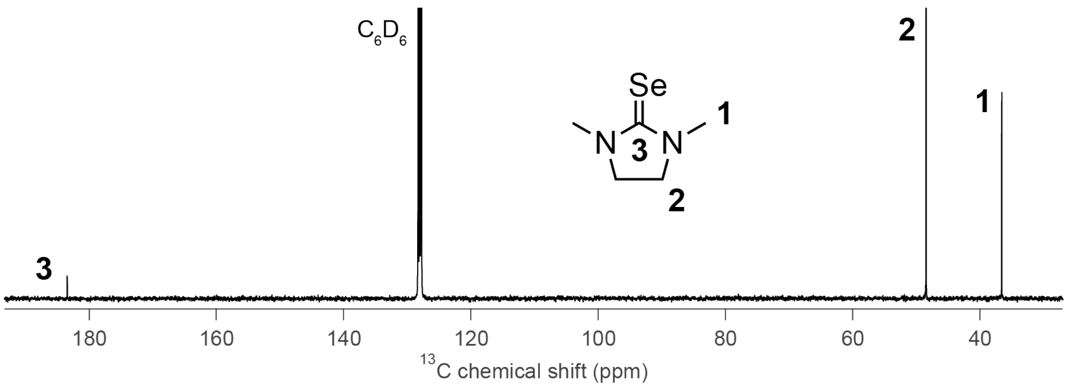


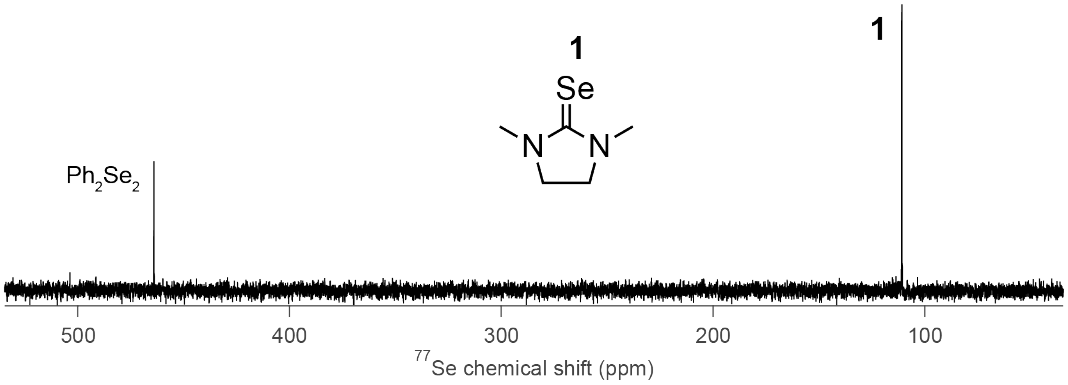


**1,3-diethylimidazolidine-2-selenone (*Se*-Im(Et$_2$))**

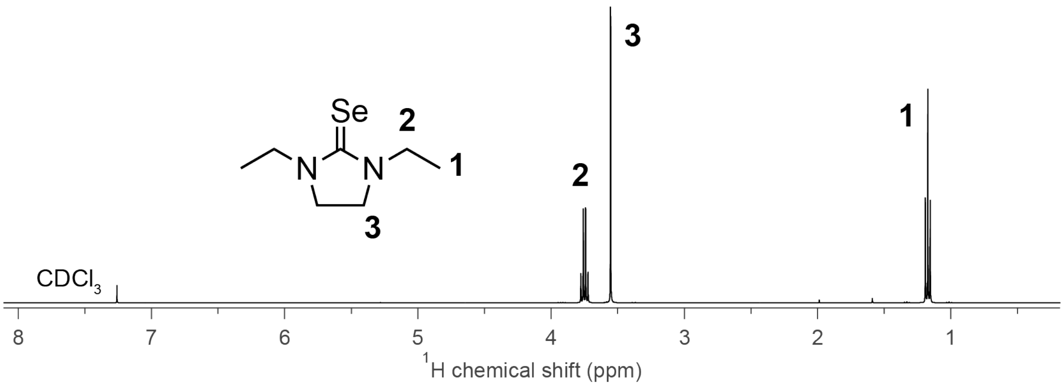

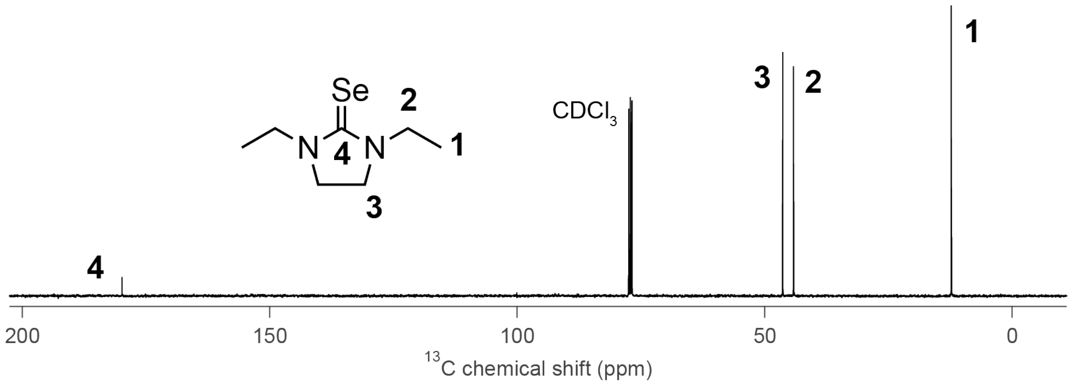


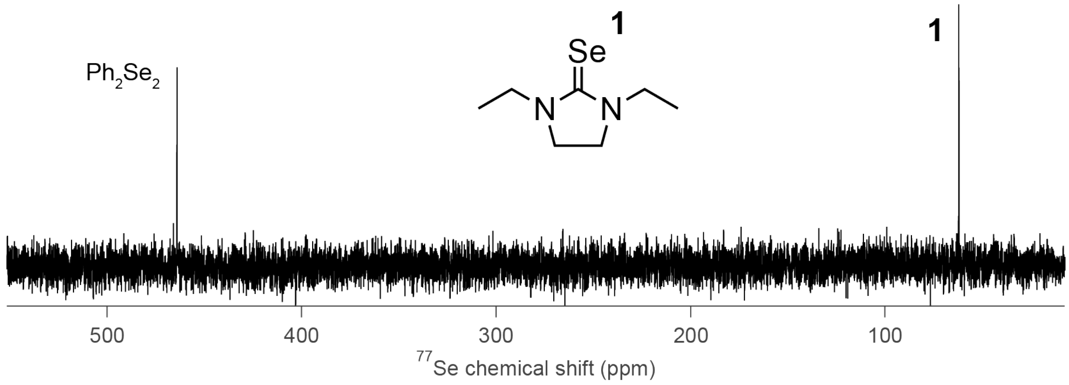


**1,3-diisopropylimidazolidine-2-selenone (*Se*-Im(iPr$_2$))**

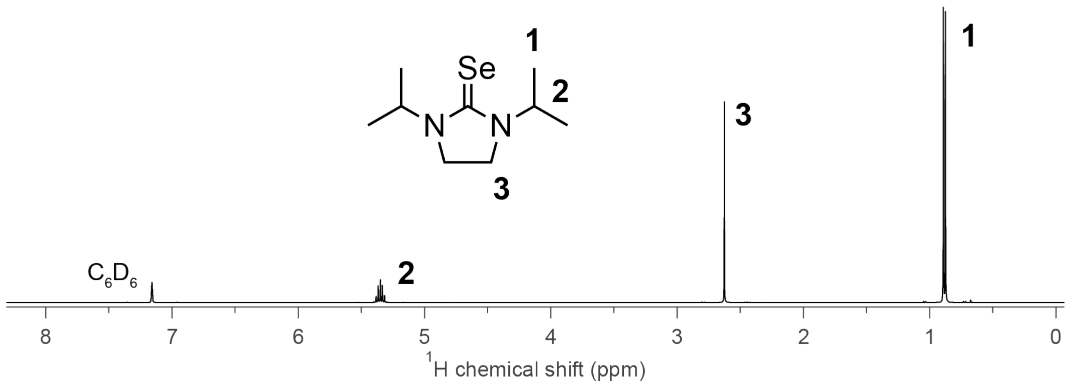

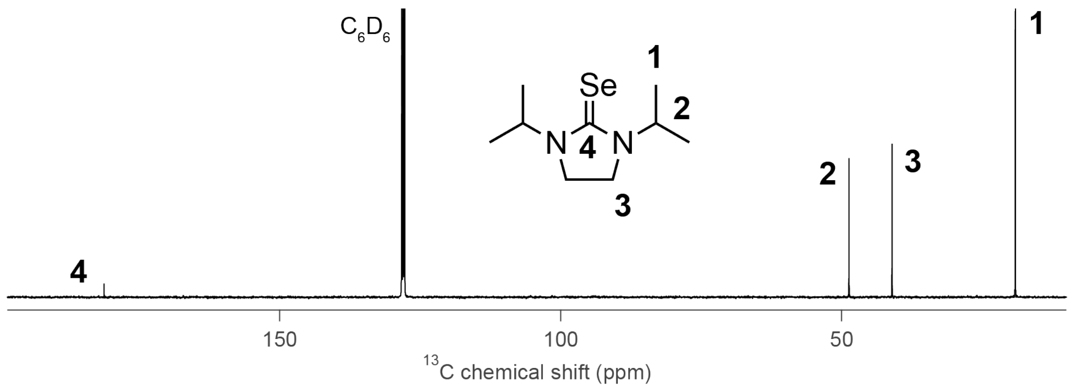


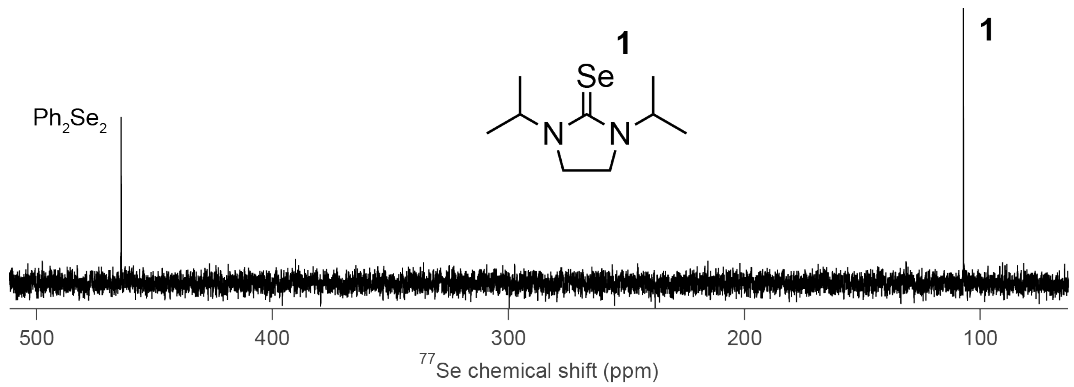


**1-methyltetrahydropyrimidine-2(1*H*)-selenone (*Se*-Pym(H,Me)).**

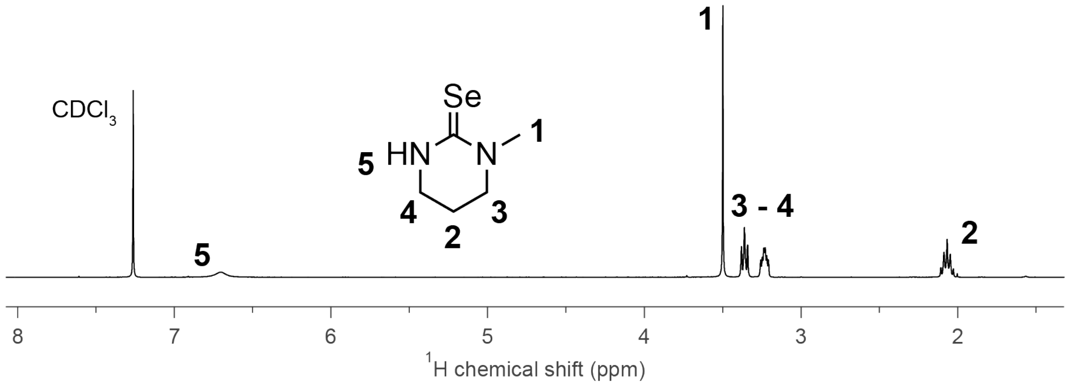

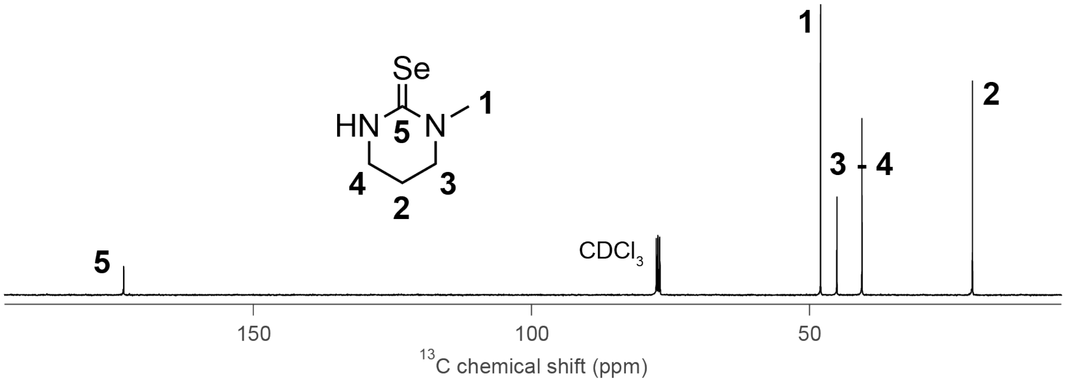


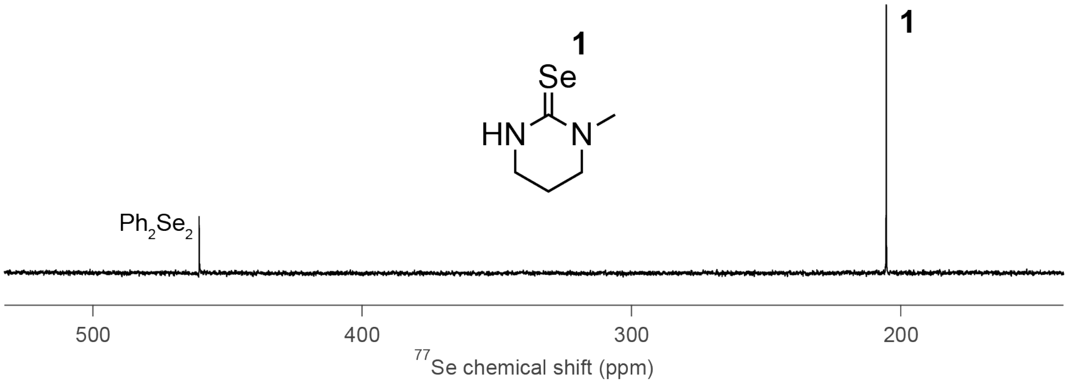


**1,3-dimethyltetrahydropyrimidine-2(1*H*)-selenone (*Se*-Pym(Me$_2$)).**

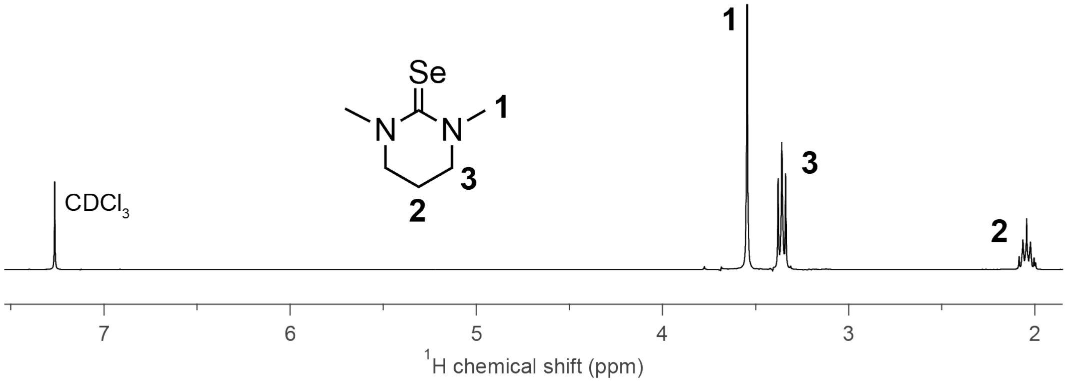

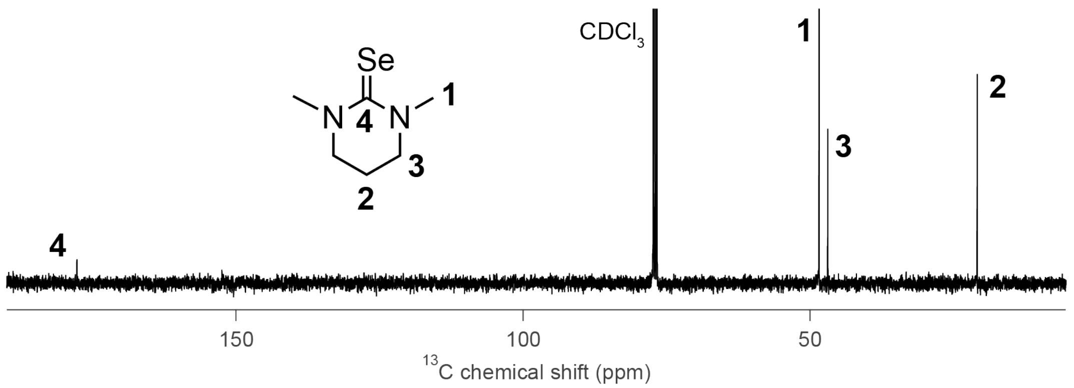


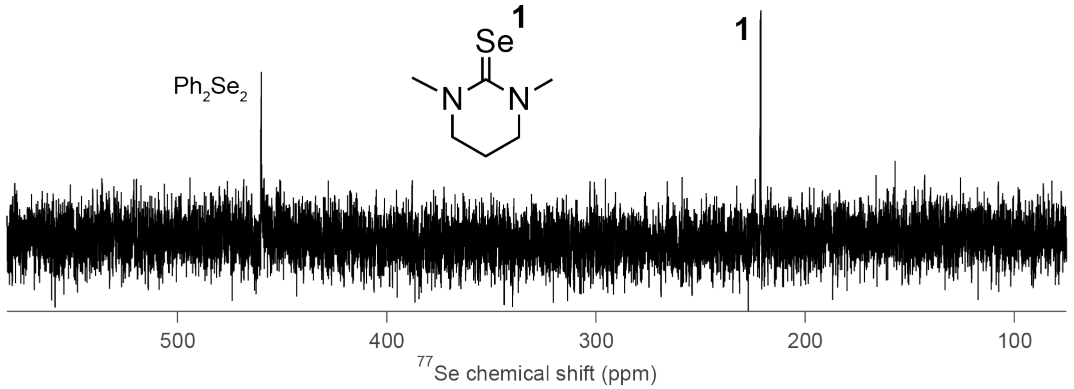


**1,3-diethyltetrahydropyrimidine-2(1*H*)-selenone (*Se*-Pym(Et$_2$)).**

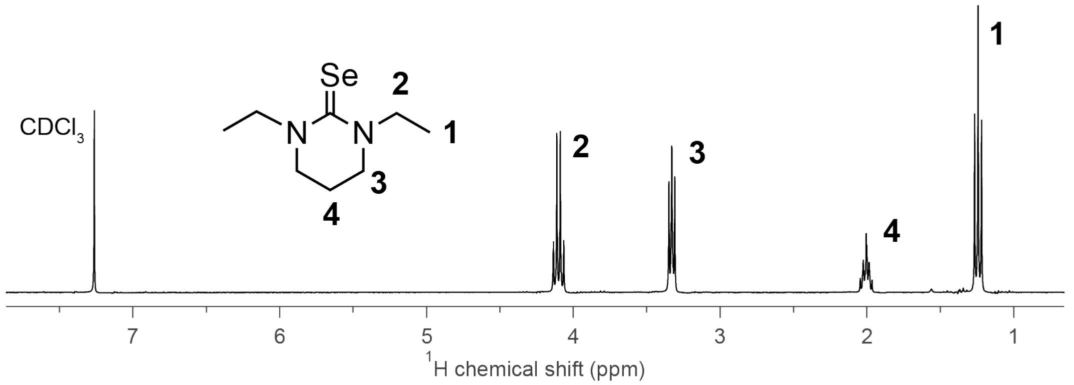

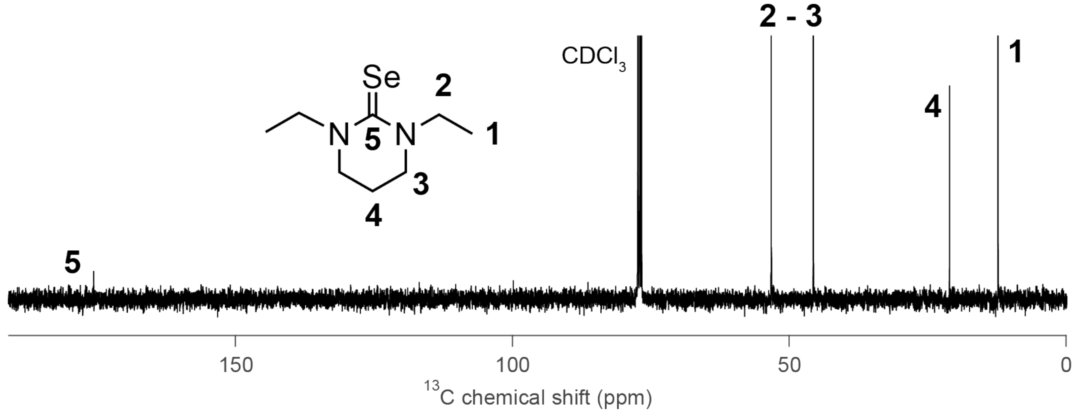


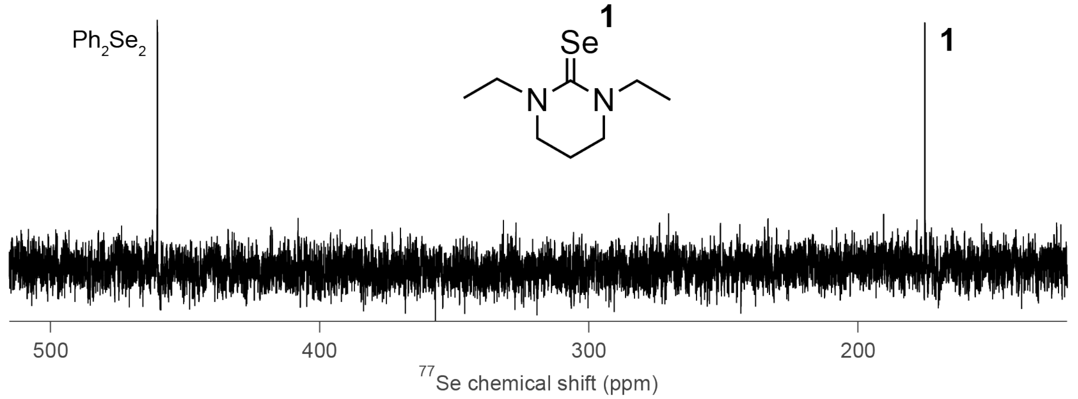


**1,3-diisopropyltetrahydropyrimidine-2(1*H*)-selenone (*Se*-Pym(iPr$_2$)).**

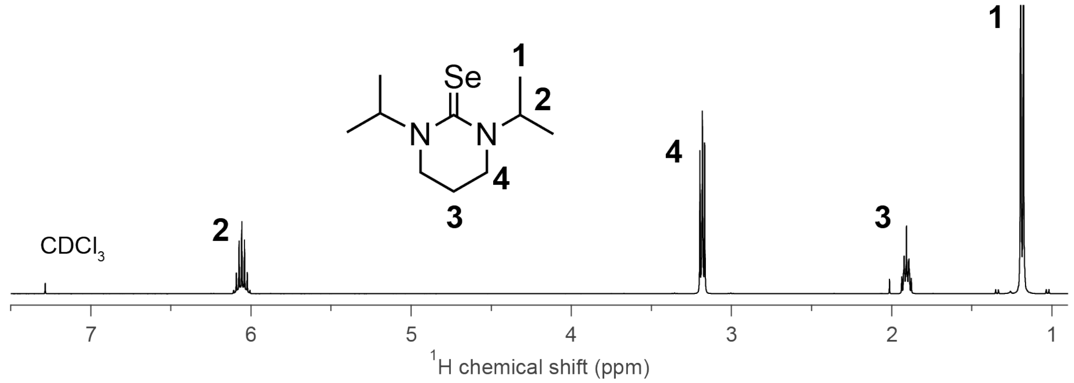

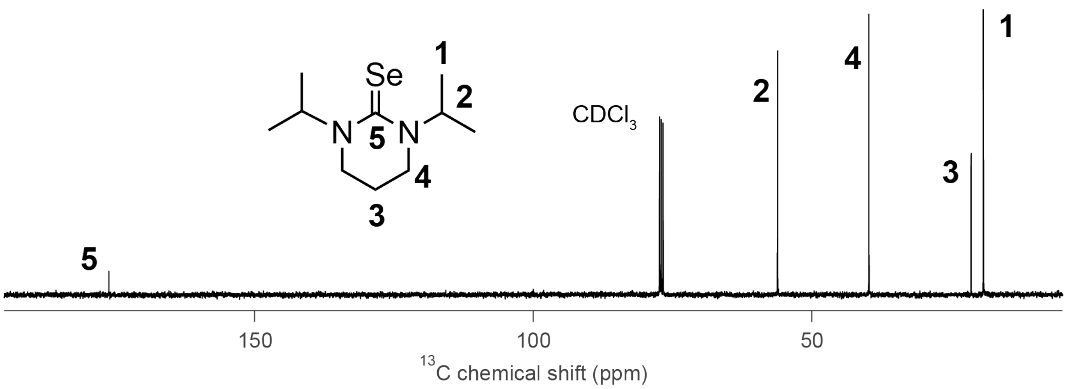

Se
1
2
5
4
3
CDCl3
2
4
3
1
5
150
100
50
13C chemical shift (ppm)


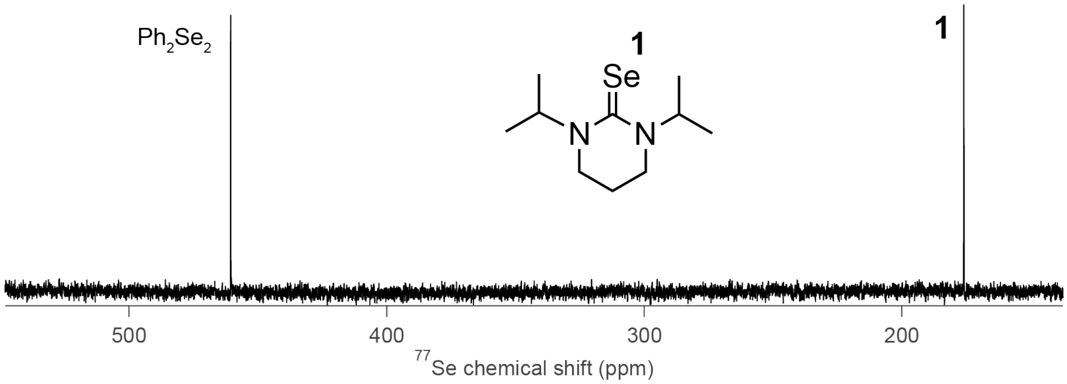

Ph2Se2
1
Se
1
500
400
300
200
77Se chemical shift (ppm)

## References


1. Li, J.; Wang, H.; Lin, L.; Fang, Q.; Peng, X., Quantitative Identification of Basic Growth Channels for Formation of Monodisperse Nanocrystals *J. Am. Chem. Soc.* **2018,** *140* (16), 5474.
2. Yu, K., CdSe Magic‐Sized Nuclei, Magic‐Sized Nanoclusters and Regular Nanocrystals: Monomer Effects on Nucleation and Growth *Adv. Mater.* **2012,** *24* (8), 1123.
3. Aubert, T.; Cirillo, M.; Flamee, S.; Van Deun, R.; Lange, H.; Thomsen, C.; Hens, Z., Homogeneously Alloyed $CdSe_{1-x}S_x$ Quantum Dots ($0 \leq x \leq 1$): An Efficient Synthesis for Full Optical Tunability *Chem. Mater.* **2013,** *25* (12), 2388.
4. Hermann, C.; Peter, Y. Y., Role of Elastic Exciton-Defect Scattering in Resonant Raman and Resonant Brillouin Scattering in CdSe *Phys. Rev. B* **1980,** *21* (8), 3675.
5. Tell, B.; Damen, T.; Porto, S., Raman Effect in Cadmium Sulfide *Phys. Rev.* **1966,** *144* (2), 771.
6. Kusch, P.; Lange, H.; Artemyev, M.; Thomsen, C., Size-Dependence of the Anharmonicities in the Vibrational Potential of Colloidal CdSe Nanocrystals *Solid State Commun.* **2011,** *151* (1), 67.
7. Chen, L.; Rickey, K.; Zhao, Q.; Robinson, C.; Ruan, X., Effects of Nanocrystal Shape and Size on the Temperature Sensitivity in Raman Thermometry *Appl. Phys. Lett.* **2013,** *103* (8), 083107.
8. Mukherjee, P.; Lim, S. J.; Wrobel, T. P.; Bhargava, R.; Smith, A. M., Measuring and Predicting the Internal Structure of Semiconductor Nanocrystals Through Raman Spectroscopy *J. Am. Chem. Soc.* **2016,** *138* (34), 10887.
9. Chang, I. F.; Mitra, S. S., Application of a Modified Random-Element-Isodisplacement Model to Long-Wavelength Optic Phonons of Mixed Crystals *Phys. Rev.* **1968,** *172* (3), 924.
10. Tschirner, N.; Lange, H.; Schliwa, A.; Biermann, A.; Thomsen, C.; Lambert, K.; Gomes, R.; Hens, Z., Interfacial Alloying in CdSe/CdS Heteronanocrystals: A Raman Spectroscopy Analysis *Chem. Mater.* **2012,** *24* (2), 311.
